# LTF induces senescence and degeneration in the meniscus via the NF-κB signaling pathway: A study based on integrated bioinformatics analysis and experimental validation

**DOI:** 10.3389/fmolb.2023.1134253

**Published:** 2023-04-24

**Authors:** Jun Zhang, Jiayong Zhu, Boming Zhao, Daibang Nie, Wang Wang, Yongjian Qi, Liaobin Chen, Bin Li, Biao Chen

**Affiliations:** ^1^ Division of Joint Surgery and Sports Medicine, Department of Orthopedic Surgery, Zhongnan Hospital of Wuhan University, Wuhan, Hubei, China; ^2^ Department of Immunology, College of Basic Medicine, Chongqing Medical University, Chongqing, China; ^3^ Chongqing Key Laboratory of Basic and Translational Research of Tumor Immunology, Chongqing Medical University, Chongqing, China; ^4^ Department of Spine Surgery and Musculoskeletal Tumor, Department of Orthopedic Surgery, Zhongnan Hospital of Wuhan University, Wuhan, Hubei, China

**Keywords:** LTF, osteoarthritis, bioinformatics, NF-κB, nuclear factor kappa B, meniscal senescence and degeneration

## Abstract

**Background:** The functional integrity of the meniscus continually decreases with age, leading to meniscal degeneration and gradually developing into osteoarthritis (OA). In this study, we identified diagnostic markers and potential mechanisms of action in aging-related meniscal degeneration through bioinformatics and experimental verification.

**Methods:** Based on the GSE98918 dataset, common differentially expressed genes (co-DEGs) were screened using differential expression analysis and the WGCNA algorithm, and enrichment analyses based on Gene Ontology (GO) terms and Kyoto Encyclopedia of Genes and Genomes (KEGG) pathways were further performed. Next, the co-DEGs were imported into the STRING database and Cytoscape to construct a protein‒protein interaction (PPI) network and further validated by three algorithms in cytoHubba, receiver operating characteristic (ROC) curve analysis and the external GSE45233 dataset. Moreover, the diagnostic marker lactotransferrin (LTF) was verified in rat models of senescence and replicative cellular senescence via RT‒qPCR, WB, immunohistochemistry and immunofluorescence, and then the potential molecular mechanism was explored by loss of function and overexpression of LTF.

**Results:** According to the analysis of the GSE98918 dataset, we identified 52 co-DEGs (42 upregulated genes and 10 downregulated genes) in the OA meniscus. LTF, screened out by Cytoscape, ROC curve analysis in the GSE98918 dataset and another external GSE45233 dataset, might have good predictive power in meniscal degeneration. Our experimental results showed that LTF expression was statistically increased in the meniscal tissue of aged rats (24 months) and senescent passage 5th (P5) meniscal cells. In P5 meniscal cells, LTF knockdown inhibited the NF-κB signaling pathway and alleviated senescence. LTF overexpression in passage 0 (P0) meniscal cells increased the expression of senescence-associated secretory phenotype (SASP) and induced senescence by activating the NF-κB signaling pathway. However, the senescence phenomenon caused by LTF overexpression could be reversed by the NF-κB inhibitor pyrrolidine dithiocarbamate (PDTC).

**Conclusion:** For the first time, we found that increased expression of LTF was observed in the aging meniscus and could induce meniscal senescence and degeneration by activating the NF-κB signaling pathway. These results revealed that LTF could be a potential diagnostic marker and therapeutic target for age-related meniscal degeneration.

## 1 Introduction

The meniscus is a pair of crescent-shaped fibrocartilage tissues located on the medial and lateral sides of the knee joint (Fox et al., 2012). The meniscus enhances joint stability between the rounded femoral condyle and the flat tibial plateau. It is essential in transferring loading, absorbing shocks, and providing nutrition and proprioception (Newman et al., 1989; Gee and Posner, 2021). Given the location and function of the meniscus in the knee, meniscal tears are one of the most common injuries to the knee joint (Fox et al., 2015). The meniscus loses elasticity with aging, becoming stiffer, and the function becomes impaired (Kulkarni and Chand, 1975). Therefore, the meniscus in middle-aged and elderly individuals undergoes degenerative changes, resulting in loss of meniscal function, increased contact pressure of the knee joint, excessive cartilage wear, and eventually osteoarthritis (OA) (Baratz et al., 1986).

Previous studies have shown that degenerative meniscal lesions are an essential feature of early OA in middle-aged and older patients (Bhattacharyya et al., 2003; Englund et al., 2012). Additionally, Goebel et al. (2017) found that degenerative meniscal tears are present early in the course of OA and are closely related to the pathological process of OA. Therefore, delaying the progression of meniscal degeneration with increasing age is of great importance for preventing meniscal injury and OA in middle-aged and elderly patients. However, the early warning signs and potential targets for therapeutic intervention of meniscal degeneration remain unclear.

Bioinformatics analysis provides methods to screen differentially expressed genes (DEGs), explore molecular mechanisms, construct a protein‒protein interaction (PPI) network, and confirm diagnostic biomarkers (Huang et al., 2018). Weighted gene co-expression network analysis (WGCNA) is one of the most commonly used bioinformatics methods, which contributes to the study of the correlation between modules and clinical features and improves the accuracy for identifying diagnostic biomarkers (Langfelder and Horvath, 2008). At present, WGCNA has been widely used for research in systemic lupus erythematosus (Zhao et al., 2021), osteonecrosis of the femoral head (Zhang J. et al., 2022) and acute myocardial infarction (Niu et al., 2019). However, to our knowledge, no studies utilizing WGCNA to identify diagnostic markers in meniscal degeneration have been reported thus far.

In this study, we downloaded the microarray datasets GSE98918 and GSE45233 from the Gene Expression Omnibus (GEO) database, screened the common differentially expressed genes (co-DEGs) of the aging meniscus in GSE98918 based on WGCNA and a series of algorithms, and analyzed the biological function of co-DEGs. Subsequently, the STRING database, Cytoscape and receiver operating characteristic (ROC) curves were analyzed to filter and identify hub genes in the aging meniscus. LTF was further identified as a potential diagnostic marker of the aging meniscus using another external GSE45233 dataset. In addition, we verified the high expression of LTF in rat models of senescence and replicative cellular senescence. Finally, we demonstrated that LTF contributed to meniscal aging and degeneration through the NF-κB pathway. Our study revealed that LTF could be a potential early warning marker and therapeutic target for meniscal aging and degeneration.

## 2 Materials and methods

### 2.1 Chemicals and reagents

Collagenase type Ⅱ, fetal bovine serum (FBS), 0.25% trypsin enzyme and penicillin/streptomycin were purchased from Gibco (Carlsbad, CA, United States). Dulbecco’s modified Eagle’s medium/F12 (DMEM/F12) and phosphate buffer saline (PBS) were obtained from HyClone Co. (Logan, United States). Primary antibodies against GLB1, LTF, P65 and GAPDH were purchased from Abclonal (Wuhan, China). The TRIzol reagent was purchased from Invitrogen Co. (Carlsbad, United States). The SYBR Green dye and reverse transcription kits were purchased from Servicebio Co., Ltd. (Wuhan, China). The SA-β-gal staining kits were obtained from Beyotime Co., Ltd (Shanghai, China). All primers were Tianyi Biotech Co., Ltd (Wuhan, China). The information of primary antibody is as follows: 3-phosphate dehydrogenase de glycéraldéhyde (GAPDH) (AC033) was purchased from Abclonal Biotech Co., Ltd. (Wuhan, China). The antibody for LTF (A12902) was purchased from Abclonal Biotech Co., Ltd. (Wuhan, China). The antibody for P65 (A2547) was purchased from Abclonal Biotech Co., Ltd. (Wuhan, China). The antibody for GLB1 (abs136187) was purchased from Absin Bioscience Inc (Shanghai, China).

### 2.2 Data source and data preprocessing

Supplementary Figure S1 illustrates the Workflow of the bioinformatics analysis We downloaded the GSE98918 and the GSE45233 microarray datasets from the GEO database (Barrett et al., 2013). The GSE98918 database contained 12 non-OA meniscal tissues and 12 OA meniscal tissues, and the details of the GSE98918 dataset are shown in Supplementary Table S1. Moreover, the GSE45233 database contained 7 non-OA meniscal tissues and 5 OA meniscal tissues. First, the GSE98918 microarray dataset was quantile normalized using the LIMMA package function “normalizeBetweenArrays” of R (Ritchie et al., 2015). Subsequently, Probes were annotated using an annotation platform file (GPL20844). Probes that did not map to any Ensembl Gene ID were removed, and if multiple probe sets mapped to the same Entrez Gene ID, only the probe set with the most highly expressed was retained. The GSE45233 microarray dataset was converted to log2 values and were quantile normalized using the LIMMA package function “normalizeBetweenArrays” of R (Ritchie et al., 2015). The probe sets were annotated using the same approach as described above.

### 2.3 Identification of differentially expressed genes (DEGs)

Based on the processed GSE98918 microarray expression dataset, we used the R (version 3.6.3) package LIMMA to screen DEGs between non-OA meniscus and OA meniscus. Raw *p*-value <0.05 and the absolute value of log fold change |log (FC)| >1.0 were used as screening thresholds, volcano plots and heatmaps were produced using the R package GGplot2 (Walter et al., 2015). To further understand the biological function of DEGs, enrichment analyses based on Gene Ontology (GO) and Kyoto Encyclopedia of Genes and Genomes (KEGG) pathways were performed using the KOBAS 3.0 (Bu et al., 2021).

### 2.4 Construction of the WGCNA network

We constructed weighted gene co-expression network analysis by the WGCNA package in the R software, then the selected top 5,000 median absolute deviation (MAD) genes to construct the representation matrix (Langfelder and Horvath, 2008). First, outlier samples were removed to guarantee a reliable network according to the sample cluster analysis of the function “hclust.” Next, Soft thresholding power was selected based on a scale-free topology criterion using the function “pickSoftThreshold.” The dynamic tree-cut algorithm was used to detect network modules with a minimum module size set to 30 and modules whose eigengenes were highly correlated (correlation above 0.75) were merged. We used the Rand index to assess the robustness of the derived clusters using the WGCNA package function “randIndex” of R and the default boostrap parameter was used (boostrap = 1000). Finally, 9 merged module eigengenes were obtained by dynamic tree cutting.

### 2.5 Identification of clinically significant modules

Key module was identified by calculating the relationship between module eigengenes and age by Pearson correlation coefficients and *p* values, and Glass Rank biserial correlation to assess the association between the gene modules and OA and sex using the rcompanion package function “wilcoxonRG” of R. Further genes information was extracted from key module. Finally, common differentially expressed genes (co-DEGs) were identified by taking the intersection of DEGs and key module genes.

### 2.6 Enrichment analysis

To further understand the biological function of DEGs and co-DEGs, enrichment analyses based on Gene Ontology (GO) and Kyoto Encyclopedia of Genes and Genomes (KEGG) pathways were performed using the KOBAS 3.0 (Bu et al., 2021). KOBAS database has been updated continually to provide more accurate and stable services for its scientific users. Overall, the current KOBAS database consists of two parts called the annotation module and the enrichment module, the enrichment module answers to which KEGG pathways and GO terms are statistically significantly associated with the input gene-list. In the KOBAS database, we need to upload the list of common differentially expressed genes using default parameters (Statistical method: hypergeometric test/Fisher’s exact test) and download the results of the enrichment analysis. The results included the GO terms and KEGG pathways and related *p* values. In the study, the GO terms and KEGG pathways with raw *p* < 0.05 were considered statistically significant, and results were visualized using the R package GGplot2 (Walter et al., 2015).

### 2.7 Construction of protein‒protein interaction (PPI) network and identification of hub gene

The Search Tool for Retrieving Interacting Genes (STRING), a database of known and predicted protein‐protein interaction (PPI), was used to construct a PPI network and identify the hub genes for the co-DEGs (Szklarczyk et al., 2019). The network was then imported into Cytoscape (version 3.9.0) for visualization (Shannon et al., 2003). Three analysis methods were used to screen out the top ten hub genes respectively, namely, Degree, Maximum Neighborhood Component (MNC) and Maximal Clique Centrality (MCC) in the Cytoscape plugin cytoHubba (Chin et al., 2014). In addition, shared hub genes were identified between three analysis methods. Enrichment analyses based on GO terms and KEGG pathways were performed as previously described for these shared hub genes using default parameters (Statistical method: hypergeometric test/Fisher’s exact test) in the KOBAS database and raw *p* < 0.05 were considered statistically significant.

### 2.8 Validation of hub genes

ROC curves are fundamental tools for diagnostic test evaluation and have been commonly used for disease screening, diagnosis, treatment, and prognosis (Obuchowski and Bullen, 2018). Based on GSE98918 dataset, we divided the samples into two groups: OA and non-OA, and according to the expression value of hub gene in each sample, ROC analysis was performed using the R package pROC, and the y-axis shows the true-positive rate (sensitivity) and the x-axis shows the false-positive rate (1−specificity). The area under the curve (AUC)is a widely used estimator of true-positive and false-positive prediction rates and based on the area under the curve (AUC) of greater than 0.9 as having excellent accuracy (Metz, 1978; Vermont et al., 1991), we evaluated the ROC curve of hub genes in the GSE98918 dataset. Moreover, these hub genes were further validated in the GSE45233 dataset.

### 2.9 Animal model

All animal experiments in this study were approved by the Committee of Laboratory Animal Experimentation of Zhongnan Hospital of Wuhan University (Wuhan, China) (permit number: ZN2021061). Rats were purchased Hubei Provincial Center for Disease Control and Prevention (Wuhan, China) (No. 42000600040610), namely, 2-month-old rats and 24-month-old rats. All animals were provided with food and water under standard environmental conditions (24°C–25 C, 12 h/12 h light/dark cycle). Rats were sacrificed by cervical dislocation after being injected intraperitoneally with pentobarbital (60 mg/kg). After disinfecting the surgical site through three rounds of 70% alcohol wrap, the knee joint was exposed and then the joint capsule opened to separate the medial and lateral meniscus. Knee joints were collected for histopathological analysis.

### 2.10 Primary meniscal cells culture

To culture primary rat meniscal cells, meniscal tissue was carefully dissected from 7-day-old male Wistar rats under sterile conditions. The synovial and the surrounding tissue were trimmed off and repeatedly flushed with PBS for 20 min. Then the meniscal tissue was cut into 1 mm^3^ size pieces with ophthalmic scissors, placed in DMEM/F12 medium containing 0.2% collagenase II, and digested 6–8 h at 37 C shakers. Afterward, centrifuged meniscal cells at 1200 rpm for 8 min. The supernatant was removed, and the cell pellet was resuspended in 70 μl of complete DMEM medium containing 10% heat-inactivated fetal bovine serum, penicillin G (100 U ml^−1^) and streptomycin (100 μg ml^−1^) at 37 C, 5% (vol/vol) CO2. The medium was refreshed at least every 2 days. Passage 0 (P0) and passage 5th (P5) cells were used for the subsequent experiments and analysis.

### 2.11 Meniscal cells process

To overexpress LTF in meniscal cells, the LTF coding sequence was obtained and cloned into the pcDNA3.1 vector (Invitrogen) to give the plasmid pcDNA-LTF. Primary meniscal cells were seeded at 0.6 × 106 cells per well in a six-well plate. According to the manufacturer’s instructions, overexpression plasmids were transfected into cells using Lipofectamine 2000 reagent (Invitrogen, Waltham, MA, United States) when cells reached about 70% confluency, and then cells were intervened in medium with or without 20 μM pyrrolidine dithiocarbamate (PDTC), an inhibitor of NF-κB. Cells were harvested 36 h post-transfection for subsequent analysis.

Specific short hairpin RNA (sh-RNA) targeting LTF mRNA were designed and cloned into pLentiLox3.7 (pLL3.7, Addgene, Cambridge, MA, United States) vectors. As previously described (Gao et al., 2012), according to the manufacturer’s instructions, lentiviral particles were produced by transfecting the psPAX2 and pMD2.G plasmids into HEK293T cells using Lipofectamine 2000 (Invitrogen, Waltham, MA, United States). After 48 h post transfection, lentiviruses were harvested and used to infect the P5 meniscal cells and stable cells were obtained by puromycin selection. The knockdown efficiency of sh-LTF was evaluated by real-time quantitative PCR (RT‒qPCR) using standard methodologies.

### 2.12 Senescence-associated β-galactosidase (SA-β gal) staining

SA-β-gal assays were performed using a cellular senescence assay kit (Servicebio, G1073), according to the manufacturer’s protocol. Meniscal cells were washed with PBS and fixed with 2% PFA and 0.2% glutaraldehyde for 15 min. After washing, fixed meniscal cells were incubated in SA-β-Gal staining overnight at (ph6.0) 37°C. Stained meniscal cells were imaged by the inverted microscope (Nikon, Tokyo, Japan). Total meniscal cells and SA-β-gal positive meniscal cells were counted in 3 random fields. Senescent cells exhibited blue staining.

### 2.13 Real-time quantitative PCR (RT‒qPCR)

Total RNAs were extracted from tissues or cells using Trizol Reagent according to manufacturer’s instruction. RNA quality and quantity were measured using the NanoDrop 1000 Spectrophotometer (Thermo Scientific, United States). cDNA was synthesized using cDNA Synthesis SuperMix Kit. RT‒qPCR was performed using a SYBR Green PCR kit with ABI StepOne instrument (Applied Biosystems, Foster City, CA, United States). Each 20 µl well reaction comprised of 10 µl of 2× SYBR qPCR Mix, 1 μl of forward primer, 1 μl of reverse primer, 6 μl of RNase-free H2O and 2 μl of cDNA templates. Reactions were run under the following conditions: 95 C for 10 min followed by 35 cycles of 15 s at 95 C, 20 s at 60°C and 15 s at 72 C. For normalization, relative gene expression was calculated for each gene by the 2^−ΔΔCT^ method with glyceraldehyde 3-phosphate dehydrogenase (GAPDH). The rat primer sequences for the genes used in this study are shown in Table 1.

**TABLE 1:** Primer sequences for RT-qPCR.

Gene	Forward primer	Reverse primer
LTF	CCG​ACG​CCA​TGA​CTC​TTT​CT	GTG​AAT​CCG​GGG​CTT​CTC​TT
Cdk2	AGC​TCT​GCT​TGC​GTT​CCA​T	ACG​TGC​CCT​CTC​CAA​TCT​TC
Cyclin D1	TCC​GGA​GAC​CGG​TCG​TAG​AG	CCG​GTC​GTT​GAG​GAG​ATT​GG
CDKN2A (P16)	GTA​GTA​CTG​CAC​CAG​GCA​GG	CCC​AGC​GGA​GGA​GAG​TAG​AT
CDKN1A (P21)	TGT​GAT​ATG​TAC​CAG​CCA​CAG​G	GCG​AAG​TCA​AAG​TTC​CAC​CG
IL-6	AGA​GAC​TTC​CAG​CCA​GTT​GC	TGC​CAT​TGC​ACA​ACT​CTT​TTC
MMP3	GCT​GTC​TTT​GAA​GCA​TTT​GGG​TT	CCC​TCC​ATG​AAA​AGA​CTC​AGA​GG
GAPDH	CTC​AAC​AGG​GAT​GCT​TAC​CCC	GAT​ACG​GCC​AAA​TCC​GTT​CA

### 2.14 Histopathologic analysis

After fixation with 4% paraformaldehyde, the fixed joint tissues were then decalcified by decalcifying solution with EDTA. After decalcification, the joint tissues were embedded in paraffin and cut into 4 μm slices. Afterward, the Safranin O-Fast Green staining was performed following the manufacturer’s instructions. The sections were imaged using a Nikon NIS Elements BR light microscope (Nikon, Tokyo, Japan).

The meniscal cells were seeded onto 6-well plates, followed by fixation in 4% paraformaldehyde for 30 min. Next, the cells were washed with PBS and stained in 0.1% safranin O solution for 30 min. Finally, the cells were briefly washed with PBS for three times, 5 min each time. A microscope (Nikon, Tokyo, Japan) was employed to observe and photograph the staining results.

### 2.15 Immunofluorescence

Meniscal cells were seeded on coverslips and fixed with 4% paraformaldehyde for 15 min, followed by PBS washes for 5 min. Then, the coverslips were treated with 0.3% Triton-100 for 15 min. After washing with PBS, the coverslips were blocked for 1 h with 3% BSA. Following another PBS wash, the coverslips were incubated with the primary antibody anti-LTF (1:100) and anti-P65 (1:100) overnight at 4°C. After washing three times with PBS, the coverslips were incubated in secondary antibody (1:100, Cy3-conjugated goat anti-rabbit IgG antibody) at 37°C for 1 h, followed by three washes with PBS. Cell nuclei were stained using 4′6′-diamidino-2-phenylindole (DAPI) for 15 min. Fluorescent images were taken using fluorescence microscopy (Nikon, Tokyo, Japan).

### 2.16 Immunohistochemical (IHC) analysis

Paraffin-embedded tissue samples were cut sliced into 4 μm. The sections were dewaxed in xylene and dehydrated in ethanol. Then, citrate buffer antigen retrieval was used on all sections. Sections were then incubated in 3% hydrogen peroxide for 10 min to inhibit endogenous peroxidase activity followed by washing three times with PBS. Afterward, sections were washed and blocked with 3% BSA and then incubated with the primary antibody overnight at 4 °C. Subsequently, sections were incubated with horseradish peroxidase (HRP) conjugated secondary antibody for 1 h at room temperature. The IHC reaction was revealed with DAB chromogen kit and images were captured with a Nikon NIS Elements BR light microscope (Nikon, Tokyo, Japan). All IHC images were analyzed with ImageJ software (National Institutes of Health, United States). All positive and negative cells were counted in each field and the percentage of total number of positive cells was calculated (positive rate (%) = number of positive cells/number of total cells × 100%).

### 2.17 Western blotting

Meniscal tissue and cells were washed twice with ice-cold PBS and lysed in 200 μl RIPA Lysis Buffer and 1 mM of phenylmethylsulfonyl fluoride (PMSF) for 5 min on ice to extract total protein. Cytoplasmic protein and nucleic protein were respectively extracted by the nuclear and cytoplasmic protein extraction kit following the manufacturer’s protocols. Equal amounts of protein lysates (30 μg per lane) were resolved by sodium dodecyl sulfate-polyacrylamide gel electrophoresis (SDS-PAGE) on 10% polyacrylamide gels, transferred to polyvinylidene difluoride membranes. They blotted with the primary antibodies for LTF (1:1000 dilution) or GAPDH (1:1000 dilution) at 4°C overnight. Band intensity was quantified using Quantity One (Bio-Rad, Shanghai, China).

### 2.18 Statistical analysis

SPSS 20.0 (SPSS Science Inc., Chicago, Illinois, United States) and Prism 8.0 (GraphPad Software, La Jolla, CA, United States) were used to analyze experimental data. All data were expressed as mean ± standard error of the mean (S.E.M.). The student’s two-tailed t-test was used to compare the mean values of various groups as applicable. A value of *p* < 0.05 was considered statistically significant.

## 3 Results

### 3.1 Construction of the WGCNA network

**FIGURE 1:**
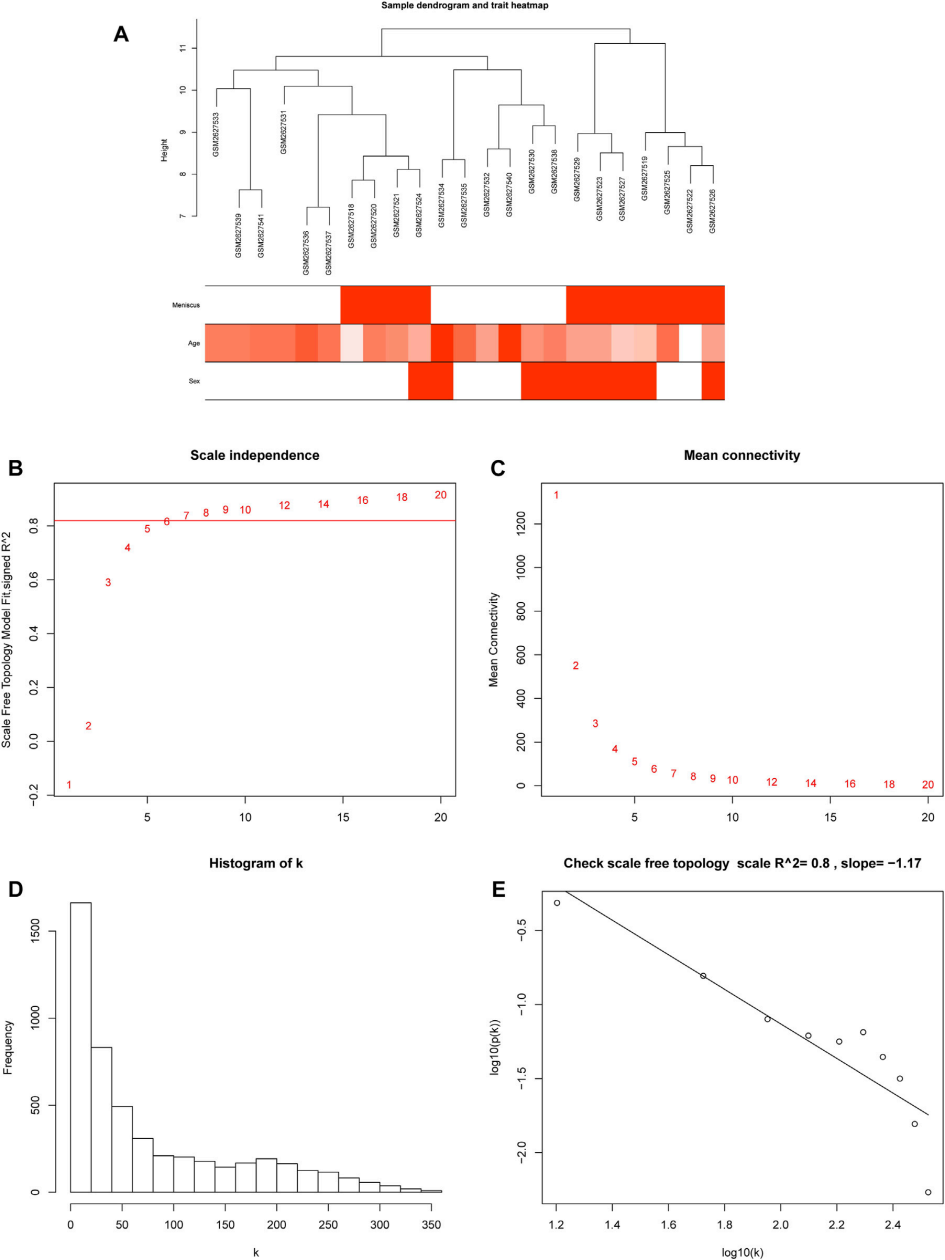
Sample clustering and soft threshold screening. **(A)** Heatmap of sample clustering and clinical traits. **(B)** Analysis of the scale-free fit index for various soft-thresholding powers (β), and 6 was the most fit power value. **(C)** Analysis of the mean connectivity for different soft-thresholding powers. **(D)** Histogram of connectivity distribution when β = 6. **(E)** Scale-free topology validation when β = 6.

The processed gene expression data were ordered according to MAD from largest to smallest, and then a WGCNA network was constructed using the top 5,000 expressed genes. Through the hierarchical clustering tree, we found that the GSE2627528 sample was distinct from other samples according to their height >12 (Supplementary Figure S2) and then removed. After filtering the outlier sample GSE2627528, the relationship between sample clustering and clinical traits are shown in Figure 1A. A soft thresholding power (β) of 6 was chosen, which was the lowest power for which the scale-free topology fit index (scale-free R2) curve flattened out upon reaching a high value of 0.8 (Figures 1B, C). The co-expression networks met the requirements of scale-free topology (Figures 1D, E).

### 3.2 Identification of co-DEGs

**FIGURE 2:**
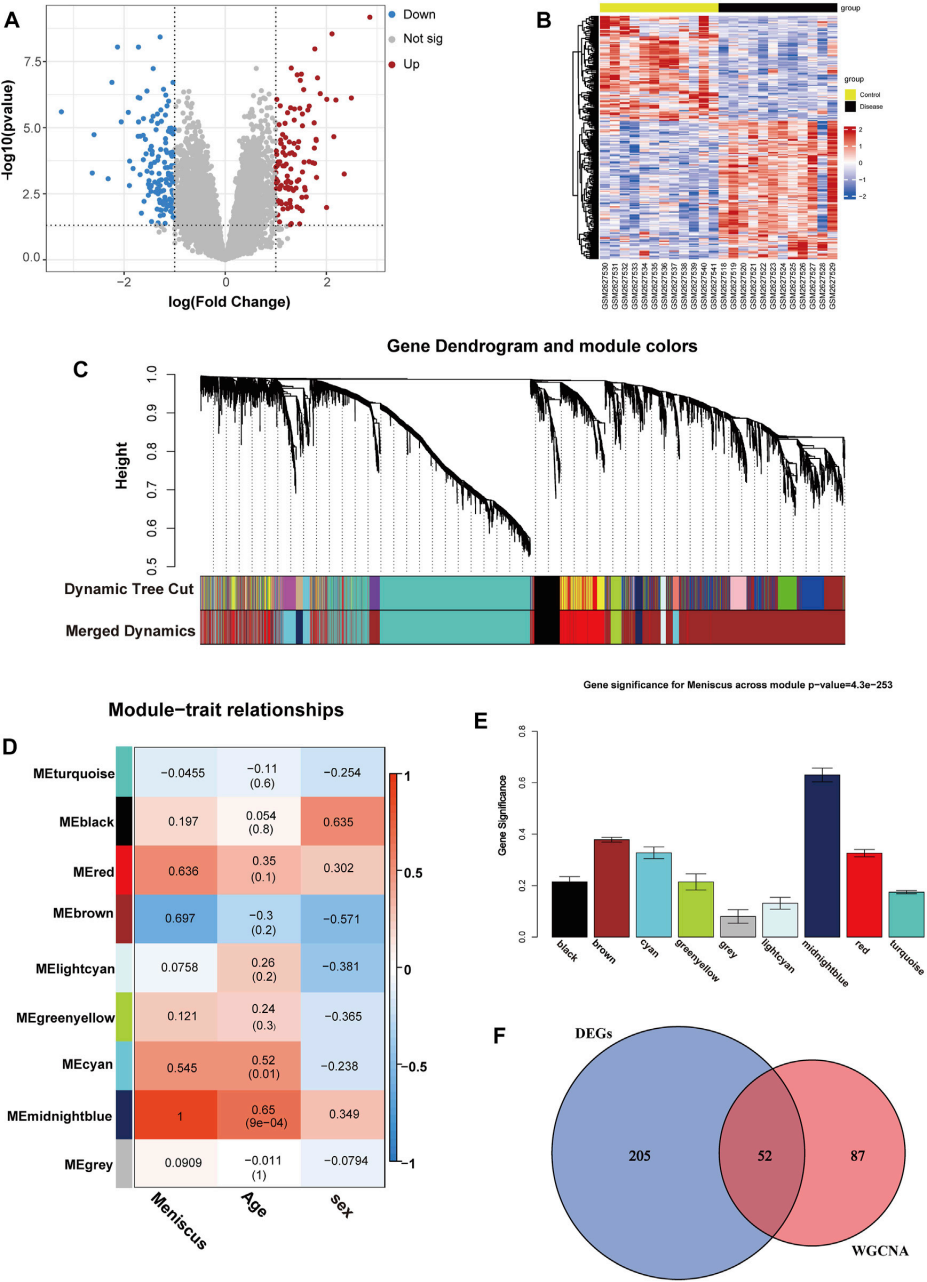
Identifications of co-DEGs. **(A)** Volcano map of DEGs, *p* < 0.05 and |logFC| >1.0 as threshold, the upregulated and downregulated genes are represented by red and blue, respectively. **(B)** Heatmap of DEGs. **(C)**The cluster dendrogram of gene modules before and after merging **(D)** Module and clinical traits correlation analysis. **(E)** Module significance values of 7 co-expression modules associated with OA meniscus. **(F)** Venn diagram of the key module genes and DEGs.

Analyzing the GSE98918 dataset, we identified 257 DEGs (111 upregulated DEGs and 146 downregulated DEGs) in OA meniscal tissues compared with non-OA meniscal tissues through differential expression analysis, and a volcano plot is shown in Figure 2A. A heatmap of DEGs is shown in Figure 2B. To know the potential pathological functions of 257 DEGs, we performed enrichment analysis and all GO terms and KEGG pathways of DEGs are listed in Supplementary Table S2. Modules were merged at a cut height of 0.25, and the minimum module size was set to 30 (Langfelder et al., 2008); thus, 17 gene modules were finally merged into 9 gene modules (Figure 2C), and the unadjusted Rand index was 0.8720651 and adjusted Rand index was 0.5769732. A correlation analysis was performed between gene modules and clinical traits. The results showed that the midnightblue gene module (including 139 genes) had the largest significant correlation with OA meniscus (rg = 1, Figure 2D) and age (cor = 0.65, *p* < 0.01, Figure 2D), and suggested that the midnightblue gene module was closely related to age. To identify markers related to meniscal aging, 52 co-DEGs (42 upregulated DEGs and 10 downregulated DEGs) were screened out by intersecting 139 genes from the midnightblue gene module with DEGs from the differential expression analysis (Figure 2F) (Supplementary Table S3).

### 3.3 Enrichment analysis of co-DEGs and identification of hub gene

**FIGURE 3:**
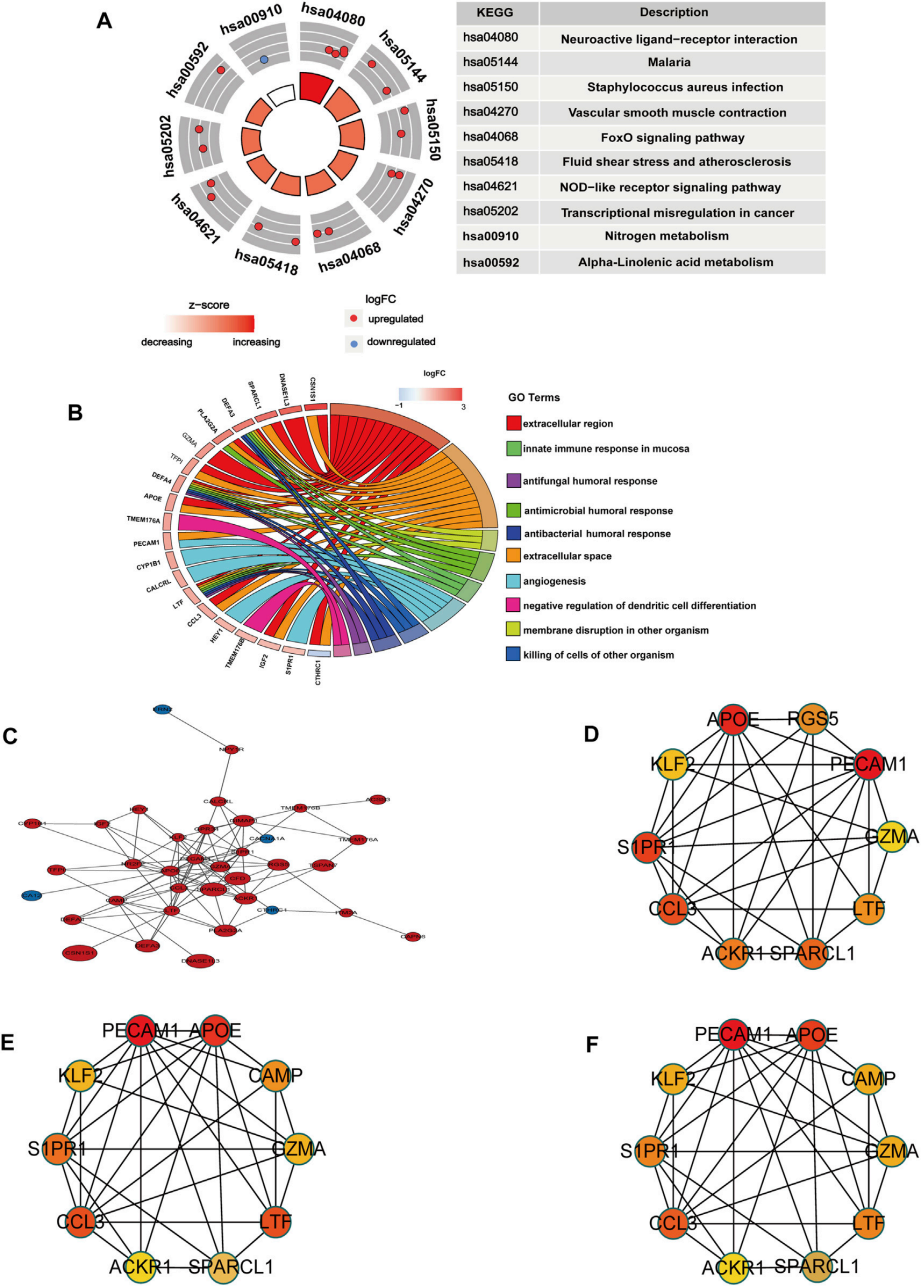
Enrichment analysis and PPI network of co-DEGs. **(A)** The circle diagram shows the enrichment analysis results of co-DEGs and the top ten KEGG pathways. Red dots represent upregulated genes and blue dots represent downregulated genes. **(B)** Chord plot shows the association of co-DEGs with the top ten GO terms, colors are represented by logFC values. **(C)** PPI network of co-DEGs, blue represents downregulated genes, red represents upregulated genes, and size represents logFC values. **(D)** Gene set network diagram of MCC algorithm in cytoHubba, the redder the color, the higher the ranking, and the yellower the color, the lower the ranking **(E)** The gene set network diagram of the Degree algorithm in cytoHubba, the redder the color, the higher the ranking, and the yellower the color, the lower the ranking. **(F)** The gene set network diagram of the MCN algorithm in cytoHubba, the redder the color in the box, the higher the ranking, and the yellower the color, the lower the ranking.

KEGG pathway enrichment and GO analyses were performed to explore the potential pathological process of OA meniscus using the KOBAS database. Ranked based on the *p*-value, we screened out the top ten KEGG pathways and GO terms. KEGG enrichment analysis showed that these co-DEGs were mainly related to neuroactive ligand‒receptor interactions, malaria, *Staphylococcus aureus* infection, vascular smooth muscle contraction, the FOXO signaling pathway, fluid shear stress and atherosclerosis, the NOD-like receptor signaling pathway, transcriptional misregulation in cancer, nitrogen metabolism, and alpha-linolenic acid metabolism (Figure 3A). GO analysis revealed that these genes were mainly involved in the extracellular region, extracellular space, membrane disruption in other organisms, antimicrobial humoral response, innate immune response in mucosa, angiogenesis, killing of cells of other organisms, antibacterial humoral response, antifungal humoral response, and negative regulation of dendritic cell differentiation (Figure 3B). All GO terms and KEGG pathways of co-DEGs are listed in Supplementary Table S4. Fifty-two co-DEGs were uploaded to the STRING database to construct PPI network and visualized by Cytoscape software. The PPI network showed 36 nodes (Supplementary Table S5), where each node represents a DEG, including 32 upregulated genes and 4 downregulated genes (Figure 3C). The top 10 genome modules were obtained from the PPI network through the MCC, MNC, and Degree methods in the cytoHubba plugin of Cytoscape software (Figures 3D–F). Nine upregulated hub genes (PECAM1, APOE, CCL3, LTF, S1PR1, SPARCL1, KLF2, GZMA, ACKR1) were identified by taking the intersection of 3 genome modules. Moreover, to know the potential pathological functions of hub genes, we performed enrichment analysis and all GO terms and KEGG pathways of hub genes are listed in Supplementary Table S6.

### 3.4 Validation of hub gene

**FIGURE 4:**
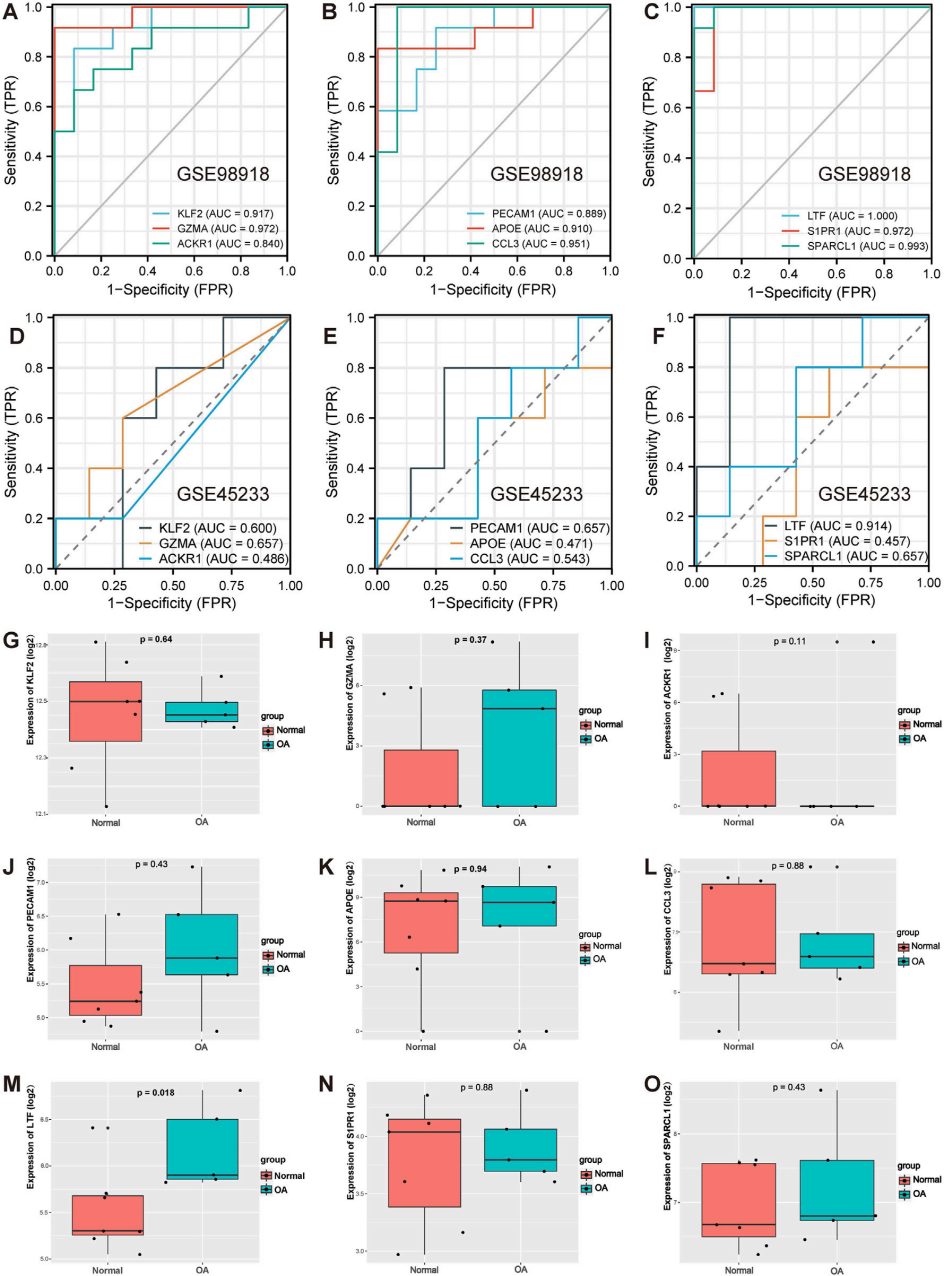
ROC curve analysis of hub genes in the GSE98918 dataset and validation of hub genes in the GSE45233 dataset. **(A)** ROC curve analysis of KLF2, GZMA, ACKR1 in the GSE98918 dataset. **(B)** ROC curve analysis of PECAM1, APOE, CCL3 in the GSE98918 dataset. **(C)** ROC curve analysis of LTF, S1PR1, SPARCL1 in the GSE98918 dataset. **(D)** ROC curve analysis of KLF2, GZMA, ACKR1 in the GSE45233 dataset. **(E)** ROC curve analysis of PECAM1, APOE, CCL3 in the GSE45233 dataset. **(F)** ROC curve analysis of LTF, S1PR1, SPARCL1 in the GSE45233 dataset. **(G)**The expression of KLF2 in the GSE45233 dataset. **(H)** The expression of GZMA in the GSE45233 dataset **(I)** The expression of ACKR1 in the GSE45233 dataset **(J)** The expression of PECAM1 in the GSE45233 dataset **(K)** The expression of APOE in the GSE45233 dataset. (**L**) The expression of CCL3 in the GSE45233 dataset. (**M**) The expression of LTF in the GSE45233 dataset (**N**) The expression of S1PR1 in the GSE45233 dataset. (**O**) The expression of SPARCL1 in the GSE45233 dataset.

Next, the diagnostic sensitivity of the hub genes was assessed by ROC curve analysis in the GSE98918 dataset. The area under the curve (AUC) for KLF2 was 0.917 and 95% confidence interval (95% CI) was 0.807–1.000 (Figure 4A); the AUC for GZMA was 0.972 and 95% CI was 0.913–1.000 (Figure 4A); the AUC for ACKR1 was 0.840 and 95% CI was 0.673–1.000 (Figure 4A); the AUC for PECAM1 was 0.889 and 95% CI was 0.761–1.000 (Figure 4B); the AUC for APOE was 0.910 and 95% CI was 0.780–1.000 (Figure 4B); the AUC for CCL3 was 0.951 and 95% CI was 0.853–1.000 (Figure 4B); the AUC for LTF was 1.000 and 95% CI was 1.000–1.000 (Figure 4C); the AUC for S1PR1 was 0.972 and 95% CI was 0.913–1.000 (Figure 4C); and the AUC for SPARCL1 was 0.993 and 95% CI was 0.974–1.000 (Figure 4C). In addition, these 9 hub genes were further validated in another external GSE45233 dataset, and the results showed the AUC for LTF was 0.914 and 95% CI was 0.733–1.000 (Figure 4F), the AUC for KLF2 was 0.600 and 95% CI was 0.241–0.959 (Figure 4D), the AUC for GZMA was 0.657 and 95% CI was 0.341–0.973 (Figure 4D), the AUC for ACKR1 was 0.486 and 95% CI was 0.195–0.776 (Figure 4D), the AUC for PECAM1 was 0.657 and 95% CI was 0.271–1.000 (Figure 4E), the AUC for APOE was 0.471 and 95% CI was 0.094–0.848 (Figure 4E), the AUC for CCL3 was 0.543 and 95% CI was 0.172–0.914 (Figure 4E), the AUC for S1PR1 was 0.457 and 95% CI was 0.088–0.826 (Figure 4F), the AUC for SPARCL1 was 0.657 and 95% CI was 0.317–0.998 (Figure 4F). Moreover, the expression of these 9 hub genes was also validated in the GSE45233 dataset, and the results showed that log2FC for LTF was 1.79 and the expression of LTF was higher in the OA meniscus group (*p* < 0.05, Figure 4M). However, no significant difference in the gene expression of KLF2 (*p* = 0.64, Figure 4G), GZMA (*p* = 0.37, Figure 4H), ACKR1 (*p* = 1, Figure 4I), PECAM1 (*p* = 0.43, Figure 4J), APOE (*p* = 0.94, Figure 4K), CCL3 (*p* = 0.88, Figure 4L), S1PR1 (*p* = 0.88, Figure 4N) or SPARCL1 (*p* = 0.43, Figure 4O) was observed. Based on the high predictive power of AUC >0.9 (Obuchowski and Bullen, 2018), these results suggested that LTF might be a diagnostic marker for meniscal degeneration.

### 3.5 Increased expression of LTF was observed in aged meniscal tissues and senescent cells

**FIGURE 5:**
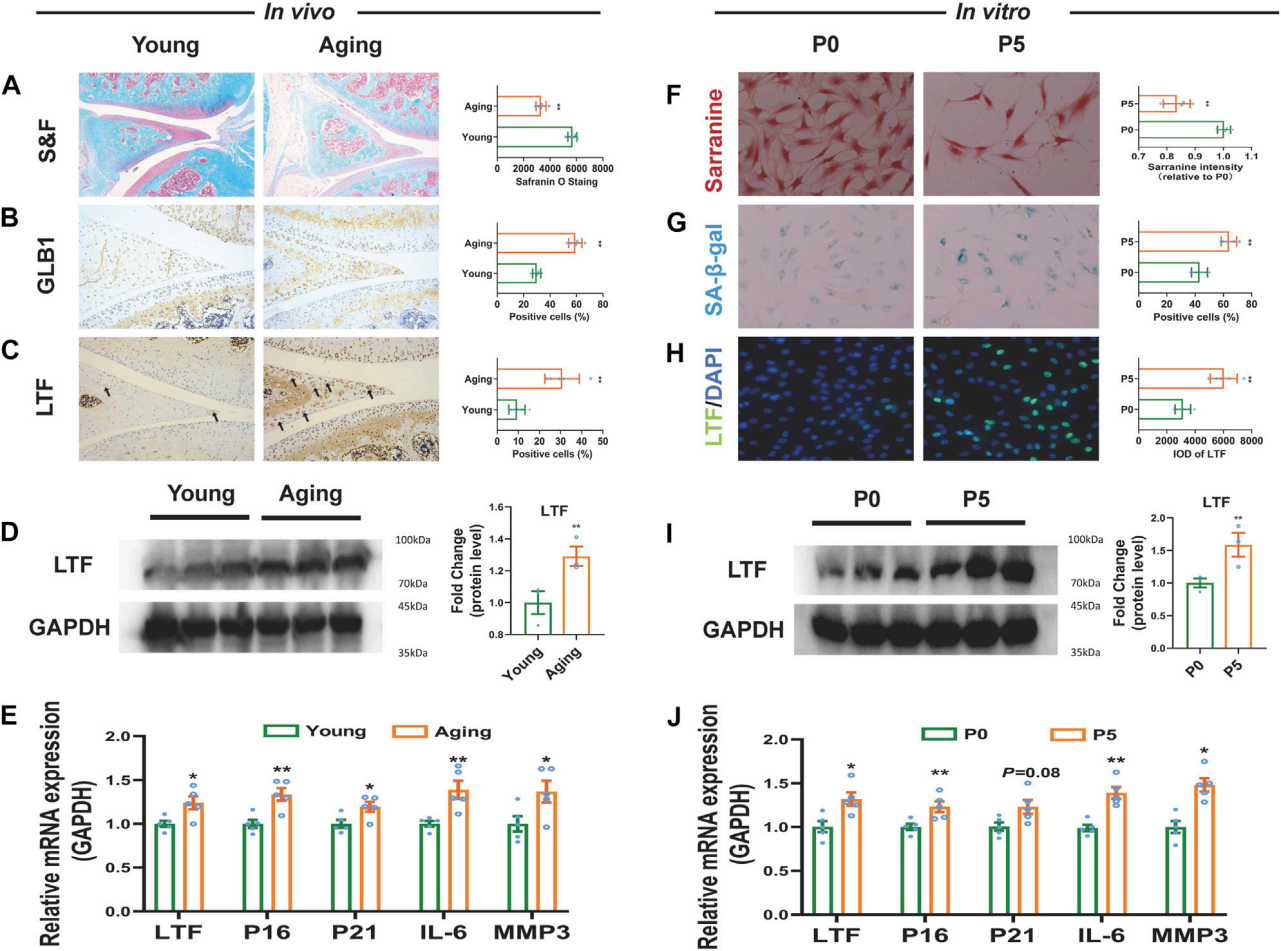
The expression of LTF in aged meniscal tissues and senescent meniscal cells of rats. **(A)** Representative images and quantitative analysis of Safranin-O Fast staining *in vivo*, scale bar = 50 μm, n = 5. **(B, C)** Representative immunohistochemistry images and quantitative analysis for GLB1 and LTF *in vivo*, scale bar = 50 μm, n = 5. **(D)** LTF protein expression *in vivo*, n = 3. **(E)** The mRNA expression of LTF, P16, P21, IL-6 and MMP3 *in vivo*, n = 5 **(F)** Representative images and quantitative analysis of Safranin-O staining *in vitro*, scale bar = 50 μm, n = 5 **(G)** Representative images and quantification of SA-β-Gal positive cells of SA-β-Gal staining *in vitro*, scale bar = 50 μm, n = 5 **(H)** Representative images and quantitative analysis of immunofluorescence for LTF *in vitro*, scale bar = 50 μm, n = 5 **(I)** LTF protein expression *in vitro*, n = 3**(J)** The mRNA expression of LTF, P16, P21, IL-6 and MMP3 *in vitro*. Mean ± S.E.M., ^*^ *p* < 0.05, ^**^ *p* < 0.01 compared with control groups. S&F, safraninO-Fast green; GLB1, galactosidase beta 1; LTF, lactotransferrin; SA-β-Gal, senescence-associated β-galactosidase; P16 (CDKN2A), cyclin dependent kinase inhibitor 2A; P21 (CDKN1A), cyclin dependent kinase inhibitor 1A; IL-6, interleukin 6; MMP3, matrix metallopeptidase 3.

The above WGCNA showed that the increased expression of LTF was involved in the age-related OA meniscus module, suggesting that high expression of LTF is closely related to meniscal aging. Therefore, the expression level of LTF was further validated in the meniscus of the senescent rat model (Sengupta, 2013). Safranin O-Fast Green staining showed that, compared to the young group (2-month-old rats), the glycosaminoglycan (GAG) content in the meniscal tissue of the aged group (24-month-old rats) was significantly decreased (*p* < 0.01, Figure 5A). Immunohistochemical staining showed that the expression of GLB1 and LTF were elevated with statistically significant (*p* < 0.01, Figures 5B, C). in the aging group. RT‒qPCR results showed that the mRNA expression of senescence-associated genes CDKN2A (P16) and CDKN1A (P21) and senescence-associated secretory phenotype (SASP)-related markers IL-6 and MMP3 were increased in the meniscal tissue of aging rats (*p* < 0.05, *p* < 0.01, Figure 5E). At the same time, we observed that the protein and mRNA expression of LTF were increased in the meniscal tissue of aging rats (*p* < 0.05, *p* < 0.01, Figures 5C–E). Furthermore, the expression level of LTF was validated based on the replicative cellular senescence model (Thompson et al., 2017). The results showed that compared with the P0 cells, the GAG content was decreased and SA-β-gal staining was enhanced in the P5 meniscal cells (*p* < 0.01, Figures 5F, G). The mRNA expression levels of P16, P21, IL-6 and MMP3 were increased in the P5 meniscal cells (*p* < 0.05, *p* < 0.01, Figure 5J). Similarly, we also observed that the protein and mRNA expression of LTF were statistically increased in the P5 meniscal cells (*p* < 0.05, *p* < 0.01, Figures 5H–J). The above results demonstrated that LTF expression was elevated in the senescent meniscus.

### 3.6 LTF knockdown inhibited the NF-κB signaling pathway and alleviated meniscal senescence

**FIGURE 6:**
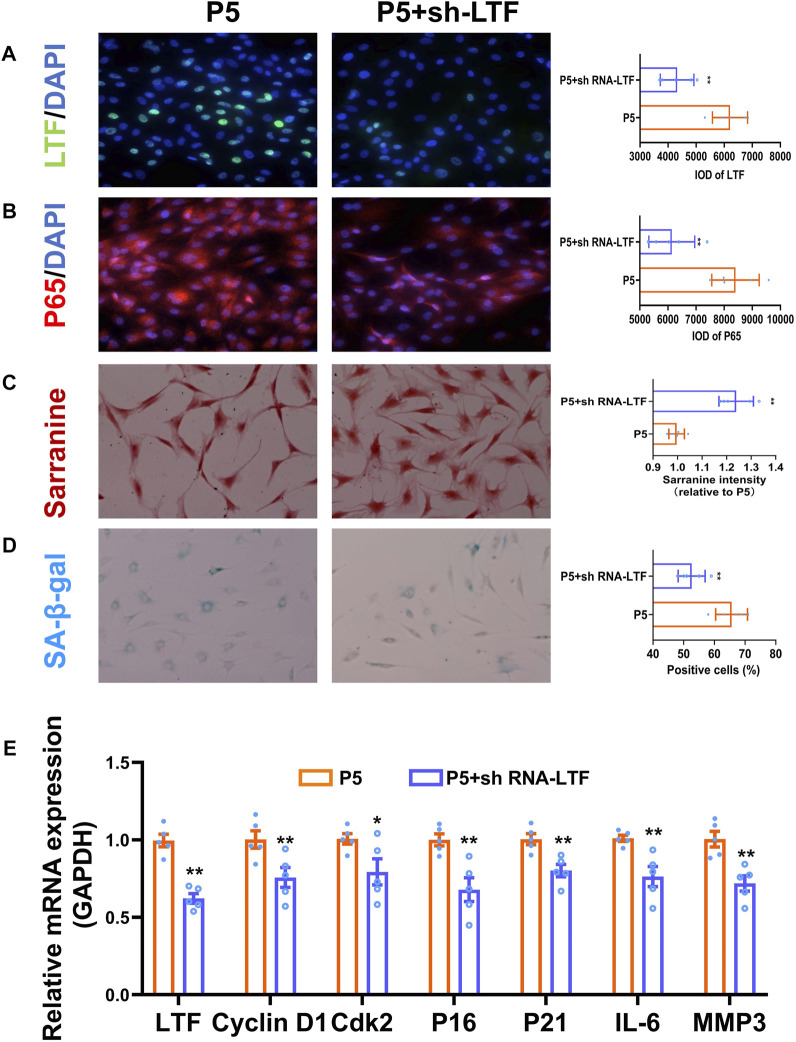
Knockdown of LTF inhibited the NF-κB signaling pathway and alleviated meniscal senescence. **(A, B)** Representative images and quantitative analysis of immunofluorescence for LTF and P65, scale bar = 50 μm. **(C)** Representative images and quantitative analysis of Safranin-O staining, scale bar = 50 μm. **(D)** Representative images and quantification of SA-β-Gal positive cells of SA-β-Gal staining cells, scale bar = 50 μm. **(E)** The mRNA expression of LTF, Cyclin D1, Cdk2, P16, P21, IL-6 and MMP3. Mean ± S.E.M., ^*^ *p* < 0.05, ** *p* < 0.01 compared with control groups, n = 5. LTF, lactotransferrin; P65, RELA proto-oncogene, NF-κB subunit; SA-β-Gal, senescence-associated β-galactosidase; P16 (CDKN2A), Cdk2, cyclin dependent kinase 2; cyclin dependent kinase inhibitor 2A; P21 (CDKN1A), cyclin dependent kinase inhibitor 1A; IL-6, interleukin 6; MMP3, matrix metallopeptidase 3.

It is known that the activation of the NF-κB signaling pathway is closely related to many aging-related diseases, such as osteoarthritis, osteoporosis and neurodegenerative diseases (Choi et al., 2019; Sivandzade et al., 2019; Kim et al., 2022). Our enrichment analysis showed that LTF potentially was involved in the activation of the NF-κB signaling pathway. However, this phenomenon has not been reported in relevant studies in the area of meniscal degeneration. Therefore, LTF knockdown was performed to investigate the potential role of LTF in senescent meniscal cells. The results showed that sh-LTF effectively reduced the expression of LTF in P5 meniscal cells (*p* < 0.01, Figures 6A, E). The GAG content was increased, and the expression of β-galactosidase was repressed in the sh-LTF-treated group (*p* < 0.01, Figures 6C, D). The protein expression and nuclear translocation of P65, a key factor in NF-κB signaling pathway, as well as the mRNA expression levels of Cyclin D1, Cdk2, P16, P21, IL-6, and MMP3, were statistically decreased by sh-LTF treatment (*p* < 0.05, *p* < 0.01, Figures 6B, E). These results suggested that knockdown of LTF could inhibit the NF-κB signaling pathway and alleviate meniscal senescence.

### 3.7 LTF contributed to meniscal senescence by activating the NF-κB signaling pathway

**FIGURE 7:**
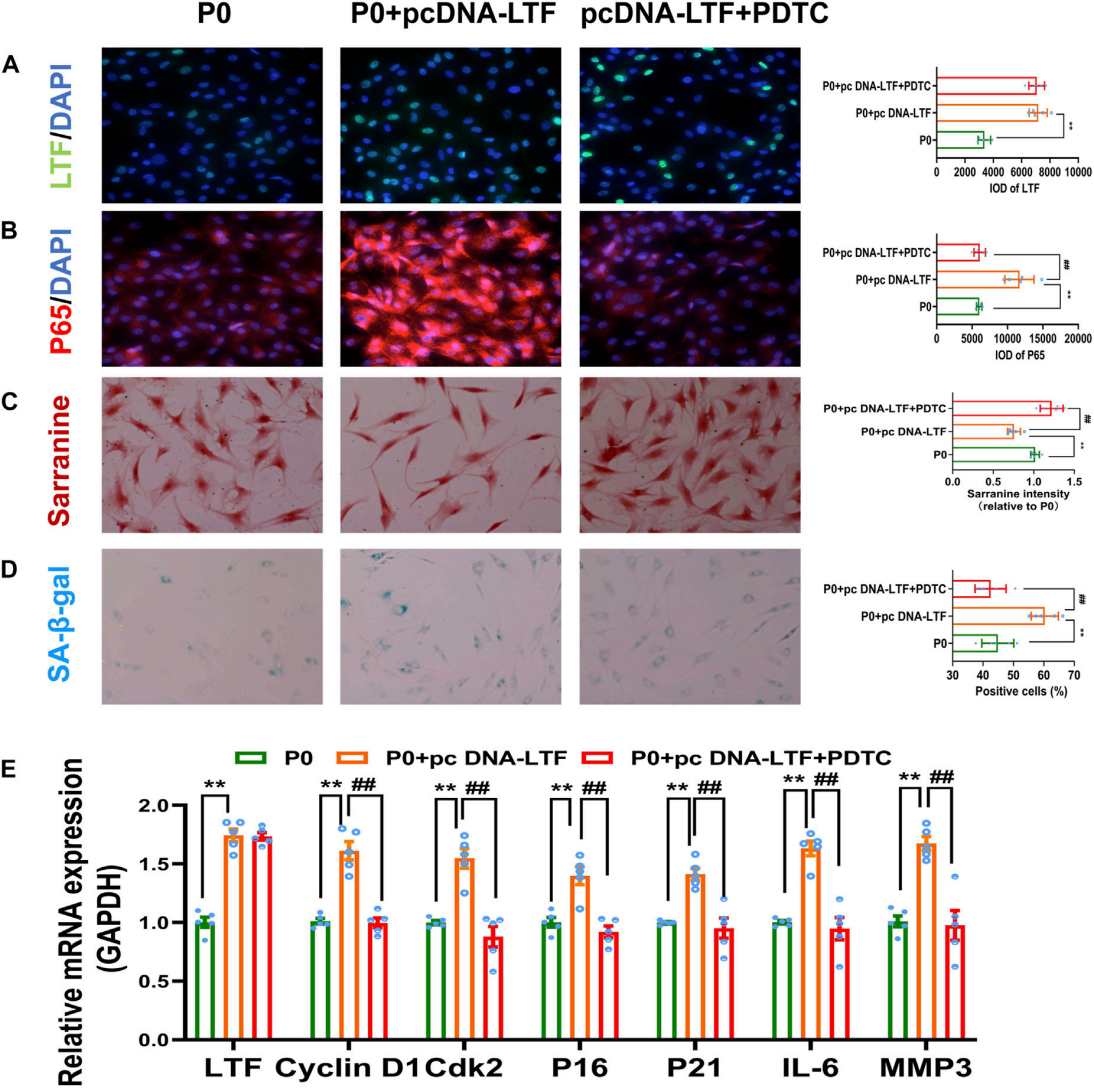
Overexpression of LTF contributed to meniscal senescence through the NF-κB signaling pathway **(A, B)** Representative images and quantitative analysis of immunofluorescence for LTF and P65, scale bar = 50 μm **(C)** Representative images and quantitative analysis of Safranin-O staining, scale bar = 50 μm **(D)** Representative images and quantification of SA-β-Gal positive cells of SA-β-Gal staining, scale bar = 50 μm **(E)** The mRNA expression of LTF, Cyclin D1, Cdk2, P16, P21, IL-6 and MMP3. Mean ± S.E.M., ^*^ *p* < 0.05, ^**^ *p* < 0.01 compared with control groups, ^#^ *p* < 0.05, ^##^ *p* < 0.01, compared with PDTC groups, n = 5. PDTC, pyrrolidine dithiocarbamate; LTF, lactotransferrin; P65, RELA proto-oncogene, NF-κB subunit; SA-β-Gal, senescence-associated β-galactosidase; Cdk2, cyclin dependent kinase 2; P16 (CDKN2A), cyclin dependent kinase inhibitor 2A; P21 (CDKN1A), cyclin dependent kinase inhibitor 1A; IL-6, interleukin 6; MMP3, matrix metallopeptidase 3.

Previous studies have shown that in cervical cancer, high expression of LTF can activate the NF-κB signaling pathway and further lead to the activation of downstream target genes (Oh et al., 2004). Additional studies suggested that LTF could activate the NF-κB signaling pathway, promoting macrophage activation (Curran et al., 2006). Therefore, we speculated that LTF was involved in mediating the occurrence of meniscal senescence through the NF-κB signaling pathway. Thus, we utilized LTF overexpression plasmid and the NF-κB inhibitor PDTC to validate this speculation in P0 cells. The results showed that pcDNA-LTF treatment effectively upregulated the expression of LTF in P0 meniscal cells (*p* < 0.01, Figures 7A, E). Moreover, increased protein expression and nuclear translocation of P65, enhanced activity of senescence-associated (SA)-β-galactosidase (SA-β-gal) and decreased GAG content were observed in the pcDNA-LTF-treated group (*p* < 0.01, Figures 7B–D). In addition, LTF overexpression promoted the mRNA expression levels of Cyclin D1, Cdk2, P16, P21, IL-6, and MMP3 (*p* < 0.01, Figure 7E). However, the phenomena above caused by LTF overexpression could be reversed by the NF-κB inhibitor PDTC (*p* < 0.05, *p* < 0.01, Figures 7A–E). These results suggested that high expression of LTF could induce meniscal senescence through the NF-κB signaling pathway.

## 4 Discussion

Age-related changes induce meniscal tissue vulnerability and lead to meniscal dysfunction (Tsujii et al., 2017). Meniscal degeneration can lead to increased brittleness of the meniscal tissue, thereby increasing the incidence of meniscal tears in the elderly population (Kwon et al., 2019). This will further promote OA development by changing the load distributions and transferring its load to the articular cartilage between the tibia and femur (Nebelung et al., 2016). Therefore, early detection of meniscal degeneration may play a role in protecting the function of the meniscus and preventing or delaying early OA (Berthiaume et al., 2005). However, the early warning signs and potential targets for therapeutic intervention of meniscal degeneration are currently unknown. Thus, it is necessary to explore diagnostic markers and intervention targets associated with meniscal degeneration to prevent the occurrence and progression of OA.

In this study, gene expression microarray dataset (GSE98918) consisted of samples from 12 OA meniscal degeneration tissues and 12 non-OA meniscal tissues. The critical genes in meniscal degeneration were screened using the WGCNA algorithm and differential expression analysis. Fifty-two genes were found to be associated with the age-related OA meniscus module. Based on the KEGG pathway enrichment analysis, we found that these genes were mainly involved in neuroactive ligand‒receptor interaction, malaria, *S. aureus* infection, vascular smooth muscle contraction, the FOXO signaling pathway, fluid shear stress and atherosclerosis, the nod-like receptor signaling pathway, transcriptional misregulation in cancer, nitrogen metabolism, and alpha-linolenic acid metabolism. Research has suggested that dysregulated FOXO transcription factors might be involved in cartilage aging and OA (Akasaki et al., 2014). Furthermore, activation of the NOD-like receptor signaling pathway induced pyroptotic inflammation and cartilage degradation in OA (Li et al., 2021). In addition, GO analysis revealed that these genes were mainly involved in the extracellular region, extracellular space and membrane disruption in other organisms. The meniscal extracellular matrix structures were gradually destroyed with meniscal degeneration, which in turn affected its function (Adams et al., 1983; Hellio Le Graverand et al., 2001). Therefore, our findings and conclusions were consistent with previous findings.

Cytoscape is an open-source software project for integrating biomolecular interaction networks with high-throughput expression data and other molecular states into a unified conceptual framework (Shannon et al., 2003). The cytoHubba plugin ranks nodes with various algorithms based on network characteristics (Chin et al., 2014). Based on three of these algorithms in this study, we identified 9 hub genes, including PECAM1, APOE, CCL3, LTF, S1PR1, SPARCL1, KLF2, GZMA and ACKR1. The predictive power of 9 hub genes was further predicted with the ROC curve in another external GSE45233 dataset, where an AUC of 0.914 were found for LTF. Moreover, the analysis of GSE45233 dataset further confirmed that LTF expression was statistically increased in the OA meniscus group. LTF is an 80-kDa iron-binding glycoprotein, a member of the transferrin family, which is present in secretions and neutrophil secondary granules (Legrand et al., 2005). Previous studies have shown that the expression of LTF increases with age and that LTF is implicated in the progression of the pathology of neurodegenerative diseases (Leveugle et al., 1994). Joshua Rowland et al. found that high LTF expression was associated with human aging and age-related kidney diseases (Rowland et al., 2019). However, there are no relevant reports of LTF affecting meniscal aging thus far. A previous study showed that the phenotypes of 24-month-old rats conformed to aging features in terms of anatomy, physiology, development and biological phenomena (Sengupta, 2013). After 5 passages, the cells were senescent, and the expression of senescence-associated protein and SASP were significantly elevated (Thompson et al., 2017; Wang et al., 2021). Therefore, we established aging models using 24-month-old rats and P5 meniscal cells. In the present study, we found that the GAG content was decreased, the mRNA expression levels of senescence-associated genes P16 and P21 and senescence-associated secretory phenotype (SASP)-related markers IL-6 and MMP3 were significantly increased, and the expression of the senescence marker GLB1 was elevated in aged rat meniscal tissues. Additionally, we observed the cellular senescence phenomenon in P5 meniscal cells. Meanwhile, high expression of LTF was confirmed in aged meniscal tissues and senescent meniscal cells. The above results demonstrated that the high expression of LTF could serve as a diagnostic marker for meniscal aging and degeneration.

Various studies have reported that excessive activation of NF-κB could induce the progression of nearly all aging-related diseases, and inhibition of NF-κB, in contrast, could reverse SASP and aging (García-García et al., 2021; Zhang X. B. et al., 2022). Considering that LTF was related to age in WGCNA (cor = 0.9, *p* < 0.01) and GO analysis results suggested that LTF was involved in the positive regulation of the NF-κB signaling pathway, we speculated that high LTF expression might contribute to meniscal aging and degeneration through the NF-κB signaling pathway. Studies have shown that LTF can activate the TLR4 signaling pathway, leading to NF-κB pathway activation and the production of subsequent proinflammatory cytokines (Ando et al., 2010). Oh et al. demonstrated that LTF overexpression could directly transmit signals to upstream regulators of NF-κB signaling, resulting in the activation of the NF-κB signaling pathway (Oh et al., 2004). Our study showed that in P5 meniscal cells, knockdown of LTF could inhibit the protein expression and nuclear translocation of P65, decrease the expression of NF-κB signaling pathway target gene (Cyclin D1, Cdk2), suppress the SASP and increase the GAG content. Meanwhile, LTF overexpression promoted the protein expression and nuclear translocation of P65, increased the expression of NF-κB signaling pathway target gene (Cyclin D1, Cdk2), increased the expression of SASP and reduced the GAG content. The phenomena above caused by LTF overexpression could be reversed by the NF-κB inhibitor PDTC. These results demonstrated that high LTF expression induced meniscal aging and degeneration through the NF-κB signaling pathway.

However, there were some limitations in this study, First, considering the difficulty in obtaining meniscal tissue from young adult, all experiments discussed in this work were carried out on rats, however, more clinical samples are needed to further validate the results. Second, the sample size in the GSE45233 dataset was small, which might induce bias, so more sample data are needed to validate our conclusions.

## 5 Conclusion

In conclusion, for the first time, we identified differential gene sets based on WGCNA and differential expression analysis and validated LTF as a diagnostic marker for age-related meniscal degeneration. Furthermore, we confirmed that LTF induced meniscal aging and degeneration by activating the NF-κB signaling pathway, which provides a potential therapeutic target for alleviating meniscal aging and degeneration.

## Figures and Tables

**Table:** 

**LTF**	Lactotransferrin
**OA**	Osteoarthritis
**P5**	Passage 5th
**P5**	Passage 0
**KOBAS**	KEGG Orthology Based Annotation System
**MAD**	Median absolute deviation
**GEO**	Gene Expression Omnibus
**GO**	Gene Ontology
**KEGG**	Kyoto Encyclopedia of Genes and Genomes
**PPI**	Protein–protein interaction
**DEGs**	Differentially expressed genes
**Co-DEGs**	Cmmon differentially expressed genes
**ROC**	Receiver operating characteristic
**KOBAS**	KEGG Orthology Based Annotation System
**STRING**	The Search Tool for Retrieving Interacting Genes
**MNC**	Maximum Neighborhood Component
**MCC**	Maximal Clique Centrality
**AUC**	Area under the curve
**PDTC**	Pyrrolidine dithiocarbamate
**SASP**	Senescence-associated secretory phenotype
**ANOVA**	Analysis of variance
**FBS**	Fetal bovine serum
**PBS**	Phosphate buffer saline
**HRP**	Horseradish peroxidase
**sh-RNA**	Short hairpin RNA
**RT-qPCR**	Real-time quantitative PCR
**SA-β gal**	Senescence-associated β-galactosidase
**GAPDH**	Glyceraldehyde 3-phosphate dehydrogenase
**IHC**	Immunohistochemical
**SE.M.**	Standard error of the mean

## Data Availability

Publicly available datasets were analyzed in this study. This data can be found here: NCBI/GSE98918 and GSE45233.
