# Supplementary material for: LTF induces senescence and degeneration in the meniscus via the NF-κB signaling pathway: A study based on integrated bioinformatics analysis and experimental validation
Source: Front Mol Biosci. 2023 Apr 24;10:1134253. doi: 10.3389/fmolb.2023.1134253 (PMC10164984; doi:10.3389/fmolb.2023.1134253)
Supplement: Supplementary file 1 [file Table1.DOCX]

**Table S1: Details of the GSE98918 dataset.**

| **Samples** | **tissues** | **Age (years)** | **Sex** |
| --- | --- | --- | --- |
| GSM2627518 | arthroscopic partial meniscectomy | 37 | Female |
| GSM2627519 | arthroscopic partial meniscectomy | 45 | male |
| GSM2627520 | arthroscopic partial meniscectomy | 62 | Female |
| GSM2627521 | arthroscopic partial meniscectomy | 58 | Female |
| GSM2627522 | arthroscopic partial meniscectomy | 31 | Female |
| GSM2627523 | arthroscopic partial meniscectomy | 53 | male |
| GSM2627524 | arthroscopic partial meniscectomy | 50 | male |
| GSM2627525 | arthroscopic partial meniscectomy | 65 | Female |
| GSM2627526 | arthroscopic partial meniscectomy | 53 | male |
| GSM2627527 | arthroscopic partial meniscectomy | 43 | male |
| GSM2627529 | arthroscopic partial meniscectomy | 53 | male |
| GSM2627530 | Osteoarthritis | 57 | male |
| GSM2627531 | Osteoarthritis | 64 | Female |
| GSM2627532 | Osteoarthritis | 53 | Female |
| GSM2627533 | Osteoarthritis | 62 | Female |
| GSM2627534 | Osteoarthritis | 80 | male |
| GSM2627535 | Osteoarthritis | 67 | Female |
| GSM2627536 | Osteoarthritis | 70 | Female |
| GSM2627537 | Osteoarthritis | 64 | Female |
| GSM2627538 | Osteoarthritis | 61 | male |
| GSM2627539 | Osteoarthritis | 62 | Female |
| GSM2627540 | Osteoarthritis | 79 | Female |
| GSM2627541 | Osteoarthritis | 64 | Female |
