# Supplementary figures and images for: LTF induces senescence and degeneration in the meniscus via the NF-κB signaling pathway: A study based on integrated bioinformatics analysis and experimental validation
Source: Front Mol Biosci. 2023 Apr 24;10:1134253. doi: 10.3389/fmolb.2023.1134253 (PMC10164984; doi:10.3389/fmolb.2023.1134253)

# Sample clustering to detect outliers

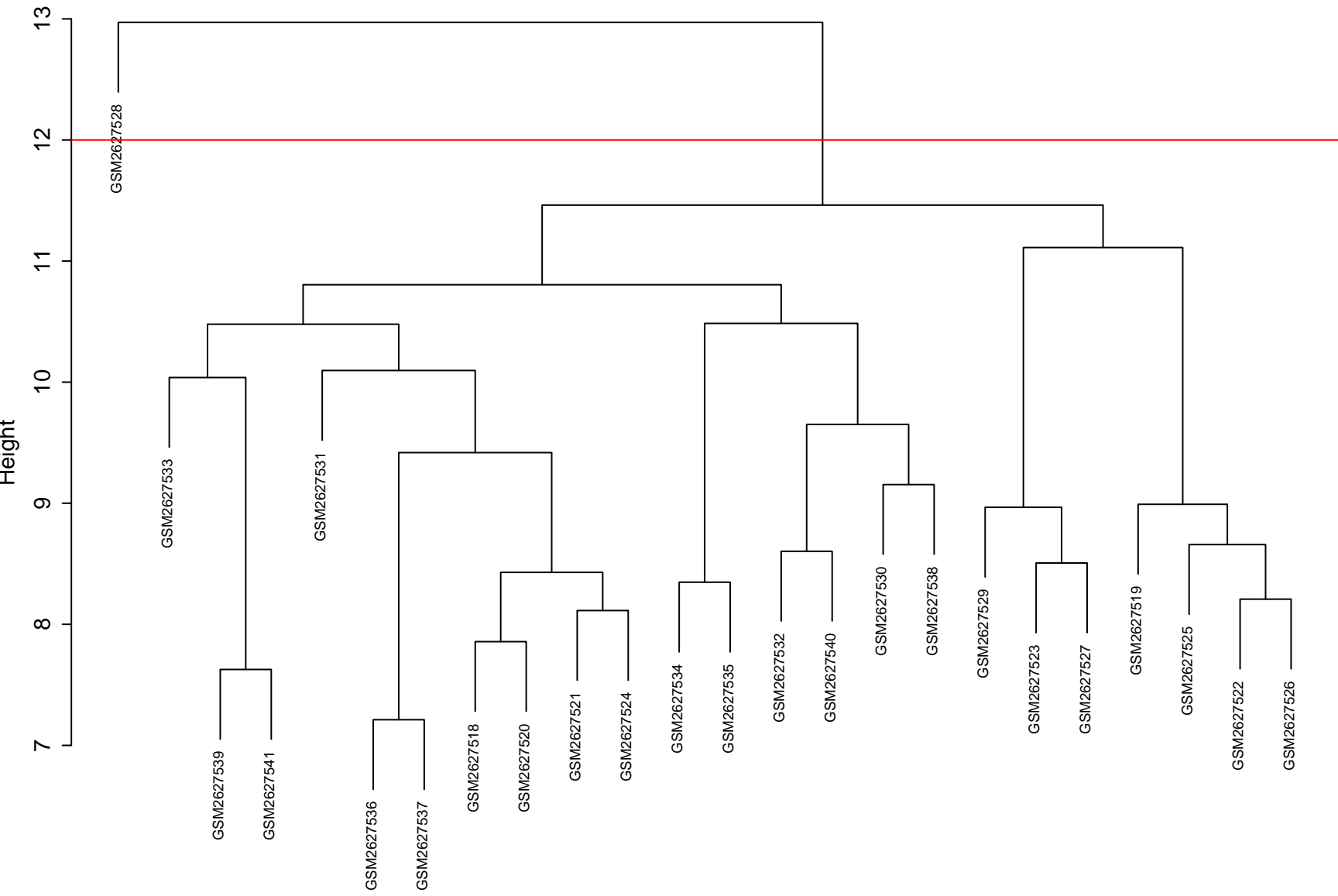

Supplement: Supplementary file 2 [file Image2.PDF]

# Bioinformatics analysis

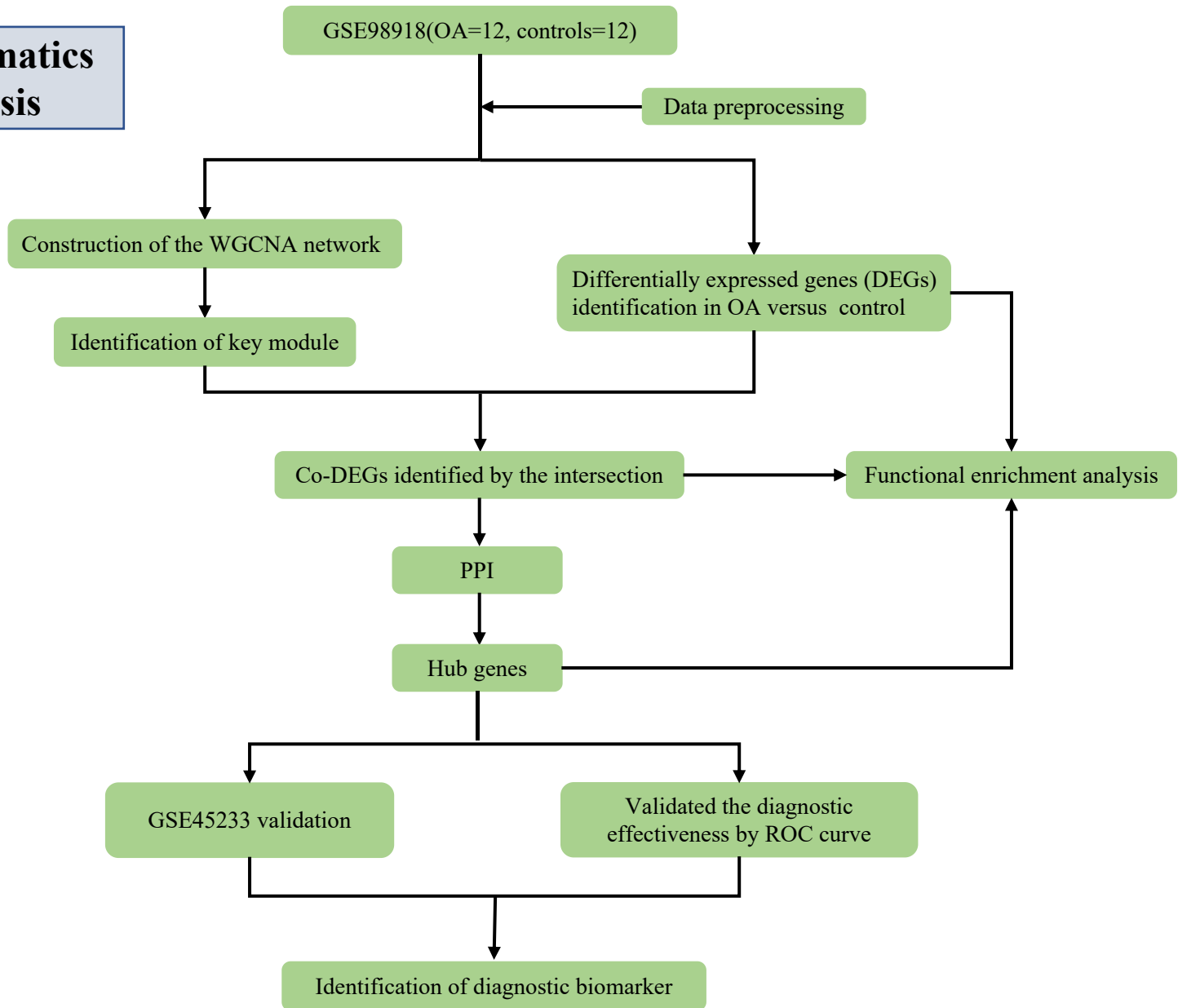

Supplement: Supplementary file 8 [file Image1.PDF]
