# Supplementary material for: LTF induces senescence and degeneration in the meniscus via the NF-κB signaling pathway: A study based on integrated bioinformatics analysis and experimental validation
Source: Front Mol Biosci. 2023 Apr 24;10:1134253. doi: 10.3389/fmolb.2023.1134253 (PMC10164984; doi:10.3389/fmolb.2023.1134253)
Supplement: Supplementary file 3 [file Table2.DOCX]

**Table S2. Detailed results of the KEGG and GO enrichment analysis of the DEGs.**

| **Group** | **ID** | **Description** | **Count** | ***P* Value** | **Input** |
| --- | --- | --- | --- | --- | --- |
| KEGG | hsa05150 | Staphylococcus aureus infection | 9 | 1.18E-09 | CFH/CFI/C1QC/C1QB/C1QA/HLA-DRB5/DEFA4/DEFA3/HLA-DRA |
| KEGG | hsa04610 | Complement and coagulation cascades | 7 | 1.06E-06 | CFH/CFI/C1QC/C1QB/C1QA/TFPI/A2M |
| KEGG | hsa05322 | Systemic lupus erythematosus | 7 | 2.68E-05 | C1QC/H2AFY2/C1QB/C1QA/HLA-DRB5/HIST1H2AI/HLA-DRA |
| KEGG | hsa04974 | Protein digestion and absorption | 6 | 2.91E-05 | CPA3/COL5A2/COL13A1/COL1A1/COL3A1/COL6A3 |
| KEGG | hsa05020 | Prion diseases | 4 | 9.56E-05 | EGR1/C1QC/C1QB/C1QA |
| KEGG | hsa05133 | Pertussis | 5 | 0.000145471 | ITGB1/IRF8/C1QC/C1QB/C1QA |
| KEGG | hsa05200 | Pathways in cancer | 12 | 0.000146677 | ITGB1/TRAF1/SPI1/FZD4/PIM1/IL13/LAMA1/MGST2/VEGFA/MMP9/IGF2/HEY1 |
| KEGG | hsa05323 | Rheumatoid arthritis | 5 | 0.000322154 | CCL3/HLA-DRA/VEGFA/IL11/HLA-DRB5 |
| KEGG | hsa04657 | IL-17 signaling pathway | 5 | 0.000354394 | S100A9/IL13/FOSL1/S100A8/MMP9 |
| KEGG | hsa04151 | PI3K-Akt signaling pathway | 9 | 0.000440457 | ITGB1/LAMA1/PPP2R3A/SGK2/NR4A1/VEGFA/COL1A1/COL6A3/IGF2 |
| KEGG | hsa04933 | AGE-RAGE signaling pathway in diabetic complications | 5 | 0.000486734 | COL3A1/COL1A1/EGR1/VEGFA/PIM1 |
| KEGG | hsa04934 | Cushing syndrome | 6 | 0.000497283 | FZD4/AIPL1/NR4A1/KCNK2/AHR/CRHR2 |
| KEGG | hsa05310 | Asthma | 3 | 0.00118234 | HLA-DRA/IL13/HLA-DRB5 |
| KEGG | hsa05165 | Human papillomavirus infection | 8 | 0.001222748 | ITGB1/LAMA1/FZD4/PPP2R3A/VEGFA/COL1A1/COL6A3/HEY1 |
| KEGG | hsa04068 | FoxO signaling pathway | 5 | 0.001607778 | CCNB2/S1PR1/RAG1/KLF2/SGK2 |
| KEGG | hsa01100 | Metabolic pathways | 19 | 0.001674475 | MGST2/MTHFD2/ACSS3/FAP/SPTLC2/CA12/ETNPPL/PLA2G2A/TYMS/FUT4/DTYMK/NT5C1B/GPX7/MRI1/NMNAT2/UAP1L1/ND3/CFD/SMOX |
| KEGG | hsa04510 | Focal adhesion | 6 | 0.001731969 | ITGB1/LAMA1/MYLK4/VEGFA/COL1A1/COL6A3 |
| KEGG | hsa05418 | Fluid shear stress and atherosclerosis | 5 | 0.002000043 | MGST2/PECAM1/VEGFA/KLF2/MMP9 |
| KEGG | hsa04512 | ECM-receptor interaction | 4 | 0.002328351 | ITGB1/LAMA1/COL6A3/COL1A1 |
| KEGG | hsa04060 | Cytokine-cytokine receptor interaction | 7 | 0.002696815 | CCL3/CCL18/IL11/IL13/GDF6/TNFRSF10D/TNFRSF12A |
| KEGG | hsa05166 | Human T-cell leukemia virus 1 infection | 6 | 0.002756004 | CCNB2/HLA-DRA/SPI1/EGR1/HLA-DRB5/FOSL1 |
| KEGG | hsa04940 | Type I diabetes mellitus | 3 | 0.002841582 | HLA-DRA/CPE/HLA-DRB5 |
| KEGG | hsa05144 | Malaria | 3 | 0.004026997 | PECAM1/ACKR1/HBB |
| KEGG | hsa05142 | Chagas disease (American trypanosomiasis) | 4 | 0.004337915 | CCL3/C1QC/C1QB/C1QA |
| KEGG | hsa04080 | Neuroactive ligand-receptor interaction | 7 | 0.005651003 | GZMA/CALCRL/S1PR1/NPY1R/GCGR/GABBR2/CRHR2 |
| KEGG | hsa05152 | Tuberculosis | 5 | 0.005685253 | CD74/HLA-DRA/CEBPG/BCL10/HLA-DRB5 |
| KEGG | hsa05145 | Toxoplasmosis | 4 | 0.005943722 | ITGB1/LAMA1/HLA-DRA/HLA-DRB5 |
| KEGG | hsa00240 | Pyrimidine metabolism | 3 | 0.006015935 | TYMS/DTYMK/NT5C1B |
| KEGG | hsa05202 | Transcriptional misregulation in cancer | 5 | 0.006635025 | TRAF1/SPI1/DEFA3/TSPAN7/MMP9 |
| KEGG | hsa05416 | Viral myocarditis | 3 | 0.006888214 | LAMA1/HLA-DRA/HLA-DRB5 |
| KEGG | hsa00670 | One carbon pool by folate | 2 | 0.007970128 | TYMS/MTHFD2 |
| KEGG | hsa04611 | Platelet activation | 4 | 0.00811978 | ITGB1/COL3A1/MYLK4/COL1A1 |
| KEGG | hsa05321 | Inflammatory bowel disease (IBD) | 3 | 0.008501039 | HLA-DRA/IL13/HLA-DRB5 |
| KEGG | hsa05205 | Proteoglycans in cancer | 5 | 0.009393982 | ITGB1/VEGFA/FZD4/IGF2/MMP9 |
| KEGG | hsa00514 | Other types of O-glycan biosynthesis | 2 | 0.009446508 | B3GALTL/GXYLT2 |
| KEGG | hsa04926 | Relaxin signaling pathway | 4 | 0.009500965 | COL3A1/COL1A1/VEGFA/MMP9 |
| KEGG | hsa05206 | MicroRNAs in cancer | 6 | 0.011665341 | CYP1B1/PIM1/EZH2/VEGFA/MMP9/FSCN1 |
| KEGG | hsa05140 | Leishmaniasis | 3 | 0.011920317 | ITGB1/HLA-DRA/HLA-DRB5 |
| KEGG | hsa04612 | Antigen processing and presentation | 3 | 0.013211055 | CD74/HLA-DRA/HLA-DRB5 |
| KEGG | hsa05412 | Arrhythmogenic right ventricular cardiomyopathy (ARVC) | 3 | 0.013211055 | ITGB1/GJA1/LAMA1 |
| KEGG | hsa04514 | Cell adhesion molecules (CAMs) | 4 | 0.013904139 | ITGB1/PECAM1/HLA-DRA/HLA-DRB5 |
| KEGG | hsa04145 | Phagosome | 4 | 0.015839133 | ITGB1/HLA-DRA/CFD/HLA-DRB5 |
| KEGG | hsa00760 | Nicotinate and nicotinamide metabolism | 2 | 0.016440489 | NMNAT2/NT5C1B |
| KEGG | hsa04630 | Jak-STAT signaling pathway | 4 | 0.019424452 | SOCS5/IL11/IL13/PIM1 |
| KEGG | hsa04658 | Th1 and Th2 cell differentiation | 3 | 0.020823865 | HLA-DRA/IL13/HLA-DRB5 |
| KEGG | hsa05222 | Small cell lung cancer | 3 | 0.021400701 | ITGB1/TRAF1/LAMA1 |
| KEGG | hsa05225 | Hepatocellular carcinoma | 4 | 0.021797419 | IGF2/ARID2/MGST2/FZD4 |
| KEGG | hsa05146 | Amoebiasis | 3 | 0.022580469 | COL3A1/LAMA1/COL1A1 |
| KEGG | hsa04640 | Hematopoietic cell lineage | 3 | 0.023795032 | IL11/HLA-DRA/HLA-DRB5 |
| KEGG | hsa04972 | Pancreatic secretion | 3 | 0.024415358 | PLA2G2A/CPA3/FAP |
| KEGG | hsa05330 | Allograft rejection | 2 | 0.025041214 | HLA-DRA/HLA-DRB5 |
| KEGG | hsa04061 | Viral protein interaction with cytokine and cytokine receptor | 3 | 0.025682078 | CCL18/CCL3/TNFRSF10D |
| KEGG | hsa05332 | Graft-versus-host disease | 2 | 0.028645813 | HLA-DRA/HLA-DRB5 |
| KEGG | hsa05219 | Bladder cancer | 2 | 0.028645813 | VEGFA/MMP9 |
| KEGG | hsa04975 | Fat digestion and absorption | 2 | 0.028645813 | PLA2G2A/FAP |
| KEGG | hsa04659 | Th17 cell differentiation | 3 | 0.030388637 | AHR/HLA-DRA/HLA-DRB5 |
| KEGG | hsa03022 | Basal transcription factors | 2 | 0.033751661 | TAF13/TAF4B |
| KEGG | hsa04670 | Leukocyte transendothelial migration | 3 | 0.03400907 | ITGB1/PECAM1/MMP9 |
| KEGG | hsa04672 | Intestinal immune network for IgA production | 2 | 0.039182315 | HLA-DRA/HLA-DRB5 |
| KEGG | hsa04071 | Sphingolipid signaling pathway | 3 | 0.039435767 | S1PR1/PPP2R3A/SPTLC2 |
| KEGG | hsa04979 | Cholesterol metabolism | 2 | 0.040588527 | NPC1/APOE |
| KEGG | hsa05320 | Autoimmune thyroid disease | 2 | 0.044919121 | HLA-DRA/HLA-DRB5 |
| KEGG | hsa04923 | Regulation of lipolysis in adipocytes | 2 | 0.047896696 | FABP4/NPY1R |
| KEGG | hsa00480 | Glutathione metabolism | 2 | 0.049411817 | MGST2/GPX7 |
| GO | GO:0005515 | protein binding | 152 | 9.62E-26 | STX1A/PLOD1/ZCCHC16/HBD/PPP2R3A/HBB/MAFB/PECAM1/CACNA1A/ROPN1L/FAM46C/CAPN6/RABIF/WEE1/HEY1/RGS3/CEP192/TSPAN2/POSTN/LPL/TMEM176A/TSPAN7/GPATCH8/TMEM176B/SOCS5/SLC2A5/NPY1R/C1QC/C1QB/C1QA/AHR/ZNF699/CCL3/HHIPL2/AIPL1/SPON2/GLIS1/ANO1/MGST2/GOLT1B/PRG4/SPRYD7/COL3A1/GABBR2/CMIP/TNFRSF12A/ARL4C/WDR41/APOE/AMER1/EGR1/MDK/FRZB/NPC1/COL13A1/MRI1/NARF/IGF2/NR4A1/SIRT4/EFEMP1/ARID2/DNASE1L3/KIAA0895L/RAG1/VEGFA/CHST1/HMCN1/GIMAP1/GJA1/MTHFD2/FCF1/IRF8/ITM2A/GPX7/TBC1D2/SYN1/CD74/FNBP4/H2AFY2/TAF13/RHOT2/TNFRSF10D/SRGN/A2M/POLH/TRMT6/SPI1/FZD4/S1PR1/TMEM200A/SS18L1/NR2F2/SYVN1/FOSL1/S100A8/KDM6B/S100A9/TPRA1/S100A2/LAMA1/MTSS1L/MYLK4/LTF/SLC43A2/FSCN1/GZMA/SLC17A9/CCL18/IL11/IL13/TRAPPC6B/WIF1/COL1A1/CRHR2/UBE2C/C16orf54/CEMIP/RASL12/CALCRL/SPARCL1/ADAM12/EZH2/HIST1H2AI/LOX/PARM1/HLA-DRA/HJURP/FAP/CAMP/ARHGAP1/CLIP3/RGS5/KLF2/ABCB4/CSN1S1/ITGB1/ULK3/ZC3H7B/PIM1/INTS8/LOXL2/LBP/CEBPG/BCL10/CCNB2/TRAF1/CFH/CFI/CFD/MMP9/C2orf40 |
| GO | GO:0005576 | extracellular region | 56 | 4.97E-23 | STX1A/CCL3/CEMIP/HHIPL2/LAMA1/SPARCL1/HBB/ADAM12/WIF1/ADAMTS14/MMP9/SRGN/COL3A1/LOX/FIBIN/A2M/LOXL1/TLL2/APOE/CPA3/FAP/ROPN1L/NPC1/DEFA4/DEFA3/S100A8/TFPI/S100A9/CSN1S1/IGF2/CRISPLD2/MDK/EFEMP1/DNASE1L3/COL5A2/LPL/VEGFA/LTF/NXPH1/PLA2G2A/GZMA/CFH/CFI/IL11/IL13/C1QC/C1QB/C1QA/SEPP1/COL13A1/GPX7/COL1A1/MXRA5/COL6A3/CTHRC1/NXPH4 |
| GO | GO:0005615 | extracellular space | 47 | 4.97E-19 | CCL3/LAMA1/CPE/HBB/SRGN/COL3A1/LOX/A2M/PECAM1/LOXL2/APOE/FZD4/S100A9/CPA3/FAP/GDF6/FRZB/DEFA4/DEFA3/S100A8/TFPI/CDNF/CSN1S1/IGF2/CRISPLD2/LOXL1/EFEMP1/MMP9/SPARCL1/COL5A2/POSTN/LPL/VEGFA/LTF/PLA2G2A/MTHFD2/CFH/CFI/CCL18/IL11/IL13/C1QC/C2orf40/COL13A1/COL1A1/COL6A3/CTHRC1 |
| GO | GO:0062023 | collagen-containing extracellular matrix | 25 | 4.60E-18 | SPARCL1/COL3A1/A2M/LOXL1/LOXL2/APOE/CPA3/S100A9/S100A8/LAMA1/MDK/EFEMP1/COL1A1/COL5A2/POSTN/MXRA5/HMCN1/EDIL3/C1QC/C1QB/C1QA/COL13A1/MMP9/COL6A3/CTHRC1 |
| GO | GO:0070062 | extracellular exosome | 41 | 7.05E-11 | CD74/SEPP1/CRISPLD2/H2AFY2/CPE/HBB/FABP4/HIST1H2AI/A2M/PECAM1/HLA-DRA/APOE/FAP/PLOD1/ARHGAP1/NPC1/DEFA3/S100A8/TM9SF2/S100A9/ITGB1/SPON2/SLC2A5/LBP/EFEMP1/ABCB4/ANO1/PLA2G2A/LPL/MXRA5/LTF/HMCN1/HLA-DRB5/FSCN1/CFH/CFI/PLXNA1/MMP9/COL6A3/IGLL5/EDIL3 |
| GO | GO:0030199 | collagen fibril organization | 9 | 7.32E-11 | LOXL1/LOXL2/CYP1B1/COL5A2/COL13A1/ADAMTS14/COL1A1/COL3A1/LOX |
| GO | GO:0030198 | extracellular matrix organization | 15 | 1.36E-10 | ITGB1/CRISPLD2/PECAM1/COL1A1/LAMA1/COL5A2/POSTN/ADAM12/COL13A1/ADAMTS14/MMP9/COL3A1/LOX/LOXL1/COL6A3 |
| GO | GO:0031012 | extracellular matrix | 14 | 5.07E-10 | SPON2/APOE/CRISPLD2/EFEMP1/LAMA1/COL5A2/POSTN/COL13A1/VEGFA/ADAMTS14/COL1A1/COL3A1/LOX/COL6A3 |
| GO | GO:0005201 | extracellular matrix structural constituent | 11 | 2.23E-09 | LAMA1/EFEMP1/EDIL3/COL5A2/POSTN/COL13A1/MXRA5/COL1A1/COL3A1/HMCN1/CTHRC1 |
| GO | GO:0050729 | positive regulation of inflammatory response | 8 | 8.17E-08 | CCL3/MDK/MGST2/PLA2G2A/FABP4/LPL/S100A9/S100A8 |
| GO | GO:0045893 | positive regulation of transcription, DNA-templated | 17 | 1.16E-07 | CD74/MDK/SPI1/FZD4/PIM1/SOX11/EGR1/GDF6/SS18L1/NR2F2/AHR/APOE/KLF2/COL1A1/MAFB/BCL10/HEY1 |
| GO | GO:0008201 | heparin binding | 10 | 1.32E-07 | MDK/APOE/CFH/CRISPLD2/FAP/POSTN/LPL/COL13A1/VEGFA/LTF |
| GO | GO:0005654 | nucleoplasm | 49 | 1.67E-07 | CAMP/AIPL1/UBE2C/H2AFY2/ANO1/TAF13/ADAM12/EZH2/RHOT2/CMIP/PARM1/POLH/SYVN1/LOXL2/SPI1/CEBPG/SMOX/SOX11/S1PR1/EGR1/NPC1/SS18L1/TATDN2/TRMT6/TAF4B/FOSL1/LBP/KDM6B/S100A9/WEE1/MRI1/INTS8/PIM1/ARID2/NR4A1/TBC1D2/TSPAN2/NR2F2/RAG1/ABCB4/HJURP/FSCN1/HEY1/GJA1/SGK2/PLXNA1/IRF8/AHR/FCF1 |
| GO | GO:0005737 | cytoplasm | 57 | 2.64E-07 | CD74/CCL3/CAMP/AIPL1/ULK3/FAP/CEMIP/ANO1/FABP4/EZH2/CDADC1/PIM1/NPC1/GABBR2/FAM46C/ARHGAP1/SYVN1/WDR41/APOE/CAPN6/CACNA1A/S1PR1/EGR1/MDK/ROPN1L/ARL4C/CLIP3/RGS5/TAF4B/S100A9/LBP/ABCB4/SPTLC2/WEE1/ITGB1/TRAF1/GJA1/FRZB/LPL/VEGFA/LTF/UNK/BCL10/FSCN1/HEY1/CCNB2/CALCRL/IL11/IRF8/C2orf40/AHR/TYMS/DTYMK/COL1A1/IL13/CTHRC1/NCF1B |
| GO | GO:0019221 | cytokine-mediated signaling pathway | 12 | 3.24E-07 | ITGB1/CCL3/FZD4/PIM1/IL11/S1PR1/IL13/STX1A/VEGFA/MMP9/SOCS5/FSCN1 |
| GO | GO:0050832 | defense response to fungus | 5 | 1.71E-06 | SPON2/DEFA4/DEFA3/S100A8/S100A9 |
| GO | GO:0019730 | antimicrobial humoral response | 6 | 1.89E-06 | PLA2G2A/DEFA4/S100A9/LTF/DEFA3/S100A8 |
| GO | GO:0043542 | endothelial cell migration | 5 | 2.68E-06 | PECAM1/LOXL2/CYP1B1/FAP/S100A2 |
| GO | GO:0030141 | secretory granule | 7 | 2.75E-06 | STX1A/CPA3/PLA2G2A/VEGFA/LTF/COL1A1/SRGN |
| GO | GO:0030669 | clathrin-coated endocytic vesicle membrane | 5 | 3.08E-06 | CD74/HLA-DRA/APOE/FZD4/HLA-DRB5 |
| GO | GO:0005581 | collagen trimer | 6 | 3.33E-06 | C1QC/C1QB/C1QA/COL13A1/LOX/CTHRC1 |
| GO | GO:0045121 | membrane raft | 10 | 3.39E-06 | ITGB1/GJA1/CFD/PECAM1/S1PR1/ANO1/CLIP3/NPC1/ABCB4/BCL10 |
| GO | GO:0001501 | skeletal system development | 8 | 3.43E-06 | IGF2/FRZB/SOX11/MMP9/COL5A2/COL13A1/COL1A1/COL3A1 |
| GO | GO:0032355 | response to estradiol | 7 | 6.24E-06 | GJA1/TFPI/NR2F2/POSTN/EZH2/COL1A1/CSN1S1 |
| GO | GO:0005886 | plasma membrane | 53 | 6.46E-06 | CD74/STX1A/CEMIP/RASL12/CPE/PLA2G2A/ADAM12/ABHD12/RHOT2/TNFRSF10D/GABBR2/TNFRSF12A/ARL4C/PARM1/FAP/PECAM1/HLA-DRA/APOE/FZD4/RGS3/CACNA1A/S1PR1/CALCRL/KCNK2/CLIP3/RGS5/S100A9/LBP/OR3A2/TFPI/MGST2/S100A8/ITGB1/SLC2A5/PIM1/ARID2/ABCB4/CA12/HLA-DRB5/ANO1/LPL/GCGR/SLC43A2/GJA1/IGDCC4/PLXNA1/NPY1R/ACKR1/ITM2A/COL13A1/AMER1/CRHR2/UBE2C |
| GO | GO:0046872 | metal ion binding | 33 | 6.75E-06 | ZNF699/CAMP/HBD/GLIS1/ANO1/HBB/ADAM12/ADAMTS14/COL3A1/POLH/ZNF354B/CACNA1A/EGR1/TATDN2/SYVN1/NPC1/KLF2/KDM6B/NARF/ITGB1/SPON2/ZC3H7B/ARID2/TMEM129/COL5A2/POSTN/RAG1/GPATCH8/UNK/NT5DC2/CFI/ZNF469/COL1A1 |
| GO | GO:0001701 | in utero embryonic development | 9 | 6.91E-06 | ITGB1/CCNB2/CMIP/FAM46C/FOSL1/KLF2/VEGFA/UNK/IGF2 |
| GO | GO:0045444 | fat cell differentiation | 6 | 7.61E-06 | NR4A1/IL11/GLIS1/GDF6/CLIP3/MAFB |
| GO | GO:0030020 | extracellular matrix structural constituent conferring tensile strength | 5 | 9.26E-06 | COL5A2/COL3A1/COL6A3/COL13A1/COL1A1 |
| GO | GO:0005509 | calcium ion binding | 16 | 9.28E-06 | DCHS1/TLL2/PPP2R3A/EFEMP1/CACNA1A/EDIL3/DNASE1L3/SPARCL1/PLA2G2A/LOXL2/LPL/S100A9/S100A8/RHOT2/HMCN1/S100A2 |
| GO | GO:0005634 | nucleus | 57 | 1.08E-05 | CD74/ZNF699/CAMP/AIPL1/GLIS1/CEMIP/CPE/FABP4/EZH2/CDADC1/SS18L1/LOX/FAM46C/ARL4C/POLH/CLIP3/LOXL2/SPI1/CEBPG/APOE/RGS3/ZNF354B/SOX11/EGR1/KCNK2/NR2F2/TRMT6/NPC1/FOSL1/KLF2/KDM6B/S100A9/WEE1/NARF/S100A8/MAFB/DNASE1L3/ZC3H7B/PIM1/NR4A1/RAG1/LTF/LBP/BCL10/FSCN1/HEY1/CCNB2/RFX8/GZMA/UBE2C/IRF8/AHR/TYMS/ZNF469/DTYMK/CACNA1A/HIST1H2AI |
| GO | GO:0098869 | cellular oxidant detoxification | 6 | 1.18E-05 | APOE/HBD/HBB/MGST2/S100A9/GPX7 |
| GO | GO:0004720 | protein-lysine 6-oxidase activity | 3 | 1.24E-05 | LOXL1/LOX/LOXL2 |
| GO | GO:0018057 | peptidyl-lysine oxidation | 3 | 1.24E-05 | LOXL1/LOX/LOXL2 |
| GO | GO:0007155 | cell adhesion | 13 | 1.24E-05 | ITGB1/SPON2/LOXL2/CYP1B1/LAMA1/S1PR1/CADM4/POSTN/ADAM12/LBP/COL6A3/EDIL3/FAP |
| GO | GO:0048514 | blood vessel morphogenesis | 4 | 1.33E-05 | LAMA1/LOX/CYP1B1/NR2F2 |
| GO | GO:0048306 | calcium-dependent protein binding | 6 | 1.36E-05 | STX1A/S100A9/S100A8/S100A2/A2M/SYN1 |
| GO | GO:0001540 | amyloid-beta binding | 6 | 1.55E-05 | CD74/APOE/FZD4/CACNA1A/C1QA/ITM2A |
| GO | GO:0030335 | positive regulation of cell migration | 9 | 1.69E-05 | ITGB1/CCL3/CEMIP/PECAM1/S1PR1/MMP9/MDK/VEGFA/COL1A1 |
| GO | GO:0061304 | retinal blood vessel morphogenesis | 3 | 1.86E-05 | CYP1B1/FZD4/LAMA1 |
| GO | GO:0008270 | zinc ion binding | 17 | 1.89E-05 | TRAF1/TLL2/NR4A1/ZCCHC16/SIRT4/RABIF/CFD/EGR1/CA12/CPE/RAG1/CDADC1/S100A8/MMP9/S100A9/NR2F2/CPA3 |
| GO | GO:0042803 | protein homodimerization activity | 15 | 2.11E-05 | PECAM1/APOE/FZD4/GZMA/FAP/DEFA3/ANO1/DEFA4/NR2F2/AHR/LPL/RAG1/CDADC1/VEGFA/FIBIN |
| GO | GO:0005783 | endoplasmic reticulum | 19 | 2.52E-05 | PLA2G2A/LOXL2/CEMIP/TMEM259/CALCRL/MGST2/DNASE1L3/TMEM129/PLOD1/SYVN1/GOLT1B/TRAPPC6B/APOE/NPC1/GPX7/TFPI/CDNF/FIBIN/GIMAP1 |
| GO | GO:0009986 | cell surface | 14 | 2.92E-05 | ITGB1/CD74/HLA-DRA/FZD4/FAP/TNFRSF10D/KCNK2/LPL/FUT4/WIF1/LTF/TFPI/VEGFA/TNFRSF12A |
| GO | GO:0009612 | response to mechanical stimulus | 5 | 3.17E-05 | COL3A1/POSTN/FOSL1/KCNK2/COL1A1 |
| GO | GO:0098883 | synapse pruning | 3 | 3.62E-05 | C1QC/C1QB/C1QA |
| GO | GO:0002544 | chronic inflammatory response | 3 | 3.62E-05 | GJA1/S100A9/S100A8 |
| GO | GO:0051673 | membrane disruption in other organism | 3 | 3.62E-05 | DEFA4/DEFA3/LTF |
| GO | GO:0001816 | cytokine production | 4 | 3.80E-05 | FABP4/FAP/S100A9/S100A8 |
| GO | GO:0072562 | blood microparticle | 7 | 3.82E-05 | APOE/CFH/HBD/C1QC/C1QB/HBB/A2M |
| GO | GO:0042802 | identical protein binding | 23 | 4.60E-05 | CD74/STX1A/CCL3/MGST2/MAFB/APOE/CEBPG/CACNA1A/FAP/GDF6/ROPN1L/MRI1/S100A2/TRAF1/MMP9/LPL/VEGFA/HJURP/BCL10/CFH/TYMS/COL1A1/SYN1 |
| GO | GO:0017147 | Wnt-protein binding | 4 | 5.52E-05 | FRZB/WIF1/FZD4/CTHRC1 |
| GO | GO:0005887 | integral component of plasma membrane | 22 | 5.94E-05 | ANO1/PRG4/GABBR2/GPR34/PECAM1/HLA-DRA/FZD4/FAP/KCNK2/NPC1/OR3A2/TM9SF2/CALCRL/ABCB4/TSPAN2/TSPAN7/GCGR/GJA1/SLC2A5/NPY1R/CRHR2/PLXNA1 |
| GO | GO:0000790 | nuclear chromatin | 17 | 7.14E-05 | SPI1/CEBPG/SOX11/IRF8/H2AFY2/NR4A1/AHR/RFX8/NPC1/EZH2/FOSL1/KLF2/KDM6B/HIST1H2AI/MAFB/EGR1/HEY1 |
| GO | GO:0002020 | protease binding | 6 | 7.28E-05 | ITGB1/FAP/BCL10/COL1A1/COL3A1/A2M |
| GO | GO:0008083 | growth factor activity | 7 | 8.84E-05 | IGF2/MDK/EFEMP1/IL11/GDF6/VEGFA/CDNF |
| GO | GO:0046982 | protein heterodimerization activity | 9 | 0.000125376 | ITGB1/TAF13/FZD4/NR4A1/H2AFY2/AHR/TAF4B/HIST1H2AI/GABBR2 |
| GO | GO:0090090 | negative regulation of canonical Wnt signaling pathway | 7 | 0.000144958 | MDK/APOE/FZD4/FRZB/AMER1/EGR1/CTHRC1 |
| GO | GO:0001525 | angiogenesis | 8 | 0.000150542 | PECAM1/CYP1B1/CALCRL/S1PR1/VEGFA/TNFRSF12A/FAP/HEY1 |
| GO | GO:0002576 | platelet degranulation | 6 | 0.00015764 | IGF2/PECAM1/SEPP1/VEGFA/SRGN/A2M |
| GO | GO:0043547 | positive regulation of GTPase activity | 9 | 0.000159475 | ITGB1/CCL3/CCL18/S1PR1/TBC1D2/RGS5/EZH2/RGS3/ARHGAP1 |
| GO | GO:0071356 | cellular response to tumor necrosis factor | 6 | 0.00016437 | CCL3/CCL18/POSTN/FABP4/KLF2/COL1A1 |
| GO | GO:0006954 | inflammatory response | 10 | 0.00016677 | CCL3/CCL18/FAP/IL13/ACKR1/TSPAN2/S100A9/S100A8/PLA2G2A/CHST1 |
| GO | GO:0030658 | transport vesicle membrane | 4 | 0.000168324 | CD74/HLA-DRA/CPE/HLA-DRB5 |
| GO | GO:0006914 | autophagy | 6 | 0.000171325 | WDR41/ULK3/IRF8/NPC1/S100A9/S100A8 |
| GO | GO:0042613 | MHC class II protein complex | 3 | 0.000173322 | CD74/HLA-DRA/HLA-DRB5 |
| GO | GO:0061053 | somite development | 3 | 0.000173322 | FRZB/SOX11/PPP2R3A |
| GO | GO:0010718 | positive regulation of epithelial to mesenchymal transition | 4 | 0.000183473 | MDK/LOXL2/EZH2/COL1A1 |
| GO | GO:0010628 | positive regulation of gene expression | 10 | 0.000204204 | CD74/CCL3/IL13/GJA1/SOX11/EGR1/VEGFA/GCGR/CAMK2N1/HEY1 |
| GO | GO:0014002 | astrocyte development | 3 | 0.00020489 | TSPAN2/S100A9/S100A8 |
| GO | GO:0043395 | heparan sulfate proteoglycan binding | 3 | 0.00020489 | LPL/APOE/CFH |
| GO | GO:0071404 | cellular response to low-density lipoprotein particle stimulus | 3 | 0.000239958 | ITGB1/SOCS5/NPC1 |
| GO | GO:0050731 | positive regulation of peptidyl-tyrosine phosphorylation | 5 | 0.000251253 | CD74/PECAM1/VEGFA/IGF2/IL11 |
| GO | GO:0000122 | negative regulation of transcription by RNA polymerase II | 15 | 0.000260159 | IGF2/LOXL2/SPI1/GLIS1/ZNF354B/SOX11/IRF8/H2AFY2/NR2F2/AHR/EZH2/VEGFA/EGR1/HEY1/KLF2 |
| GO | GO:0032496 | response to lipopolysaccharide | 6 | 0.000264273 | GJA1/LOXL1/IL13/MGST2/S100A9/S100A8 |
| GO | GO:0042060 | wound healing | 5 | 0.000292185 | COL3A1/PECAM1/LOX/S100A8/POSTN |
| GO | GO:0005178 | integrin binding | 6 | 0.000317816 | ITGB1/IGF2/EDIL3/LPL/COL3A1/FAP |
| GO | GO:0045582 | positive regulation of T cell differentiation | 3 | 0.000321231 | CD74/MDK/RAG1 |
| GO | GO:0002227 | innate immune response in mucosa | 3 | 0.000367748 | DEFA4/DEFA3/LTF |
| GO | GO:0072657 | protein localization to membrane | 3 | 0.000367748 | STX1A/CPE/TM9SF2 |
| GO | GO:0002042 | cell migration involved in sprouting angiogenesis | 3 | 0.000418386 | ITGB1/NR4A1/VEGFA |
| GO | GO:0120162 | positive regulation of cold-induced thermogenesis | 5 | 0.000426132 | GJA1/FABP4/IL13/VEGFA/KDM6B |
| GO | GO:0030225 | macrophage differentiation | 3 | 0.000473293 | SPI1/VEGFA/MMP9 |
| GO | GO:0002286 | T cell activation involved in immune response | 3 | 0.000532614 | CD74/MDK/LPL |
| GO | GO:0016021 | integral component of membrane | 39 | 0.000535495 | CD74/STX1A/TMEM259/MGST2/GOLT1B/ADAM12/TNFRSF10D/TNFRSF12A/PARM1/HLA-DRA/CACNA1A/S1PR1/TMEM200A/ABHD12/SLC43A2/NPC1/SPTLC2/ABCB4/B3GALTL/CADM4/ERN2/DCHS1/TMEM129/CA12/TSPAN2/TMEM176A/GXYLT2/TMEM176B/CHST1/GCGR/GIMAP1/SLC17A9/ACKR1/TPRA1/ITM2A/FUT4/IGDCC4/FAP/C16orf54 |
| GO | GO:0071222 | cellular response to lipopolysaccharide | 6 | 0.000548119 | SPON2/IRF8/DEFA4/DEFA3/TFPI/BCL10 |
| GO | GO:0005788 | endoplasmic reticulum lumen | 8 | 0.000618842 | APOE/SPARCL1/COL5A2/COL13A1/GPX7/COL1A1/COL3A1/COL6A3 |
| GO | GO:0001649 | osteoblast differentiation | 5 | 0.000627144 | IGF2/CCL3/LOX/GLIS1/COL1A1 |
| GO | GO:0044291 | cell-cell contact zone | 3 | 0.000665055 | GJA1/CADM4/PECAM1 |
| GO | GO:0061844 | antimicrobial humoral immune response mediated by antimicrobial peptide | 4 | 0.000736334 | S100A9/DEFA4/DEFA3/LTF |
| GO | GO:0030522 | intracellular receptor signaling pathway | 3 | 0.000738448 | AHR/NR4A1/NR2F2 |
| GO | GO:0018119 | peptidyl-cysteine S-nitrosylation | 2 | 0.000769782 | S100A9/S100A8 |
| GO | GO:0061041 | regulation of wound healing | 2 | 0.000769782 | CADM4/TNFRSF12A |
| GO | GO:0071376 | cellular response to corticotropin-releasing hormone stimulus | 2 | 0.000769782 | NR4A1/CRHR2 |
| GO | GO:0031721 | hemoglobin alpha binding | 2 | 0.000769782 | HBD/HBB |
| GO | GO:0019732 | antifungal humoral response | 2 | 0.000769782 | DEFA4/LTF |
| GO | GO:0035662 | Toll-like receptor 4 binding | 2 | 0.000769782 | S100A9/S100A8 |
| GO | GO:0007186 | G protein-coupled receptor signaling pathway | 17 | 0.000797885 | CCL3/APOE/FZD4/CALCRL/CCL18/S1PR1/NPY1R/ACKR1/KCNK2/TPRA1/RGS5/OR3A2/RGS3/GCGR/GABBR2/CRHR2/GPR34 |
| GO | GO:0044344 | cellular response to fibroblast growth factor stimulus | 3 | 0.000816801 | POSTN/NR4A1/COL1A1 |
| GO | GO:0030449 | regulation of complement activation | 5 | 0.000827024 | CFH/C1QC/C1QB/C1QA/CFI |
| GO | GO:0005802 | trans-Golgi network | 6 | 0.000891093 | POSTN/CLIP3/NMNAT2/TRAPPC6B/FUT4/CHST1 |
| GO | GO:0009617 | response to bacterium | 5 | 0.000891976 | FABP4/HMCN1/LPL/GZMA/CEP192 |
| GO | GO:0071556 | integral component of lumenal side of endoplasmic reticulum membrane | 3 | 0.000900242 | CD74/HLA-DRA/HLA-DRB5 |
| GO | GO:0022617 | extracellular matrix disassembly | 4 | 0.000919033 | TLL2/LPL/A2M/MMP9 |
| GO | GO:0031093 | platelet alpha granule lumen | 4 | 0.000919033 | SRGN/IGF2/VEGFA/A2M |
| GO | GO:0070301 | cellular response to hydrogen peroxide | 4 | 0.000969262 | EZH2/CYP1B1/KLF2/KDM6B |
| GO | GO:0001934 | positive regulation of protein phosphorylation | 6 | 0.000998216 | CD74/PECAM1/CLIP3/VEGFA/MMP9/IGF2 |
| GO | GO:2001199 | negative regulation of dendritic cell differentiation | 2 | 0.001022227 | TMEM176A/TMEM176B |
| GO | GO:0071438 | invadopodium membrane | 2 | 0.001022227 | ITGB1/FAP |
| GO | GO:0070314 | G1 to G0 transition | 2 | 0.001022227 | EZH2/C2orf40 |
| GO | GO:0050544 | arachidonic acid binding | 2 | 0.001022227 | S100A9/S100A8 |
| GO | GO:0045944 | positive regulation of transcription by RNA polymerase II | 17 | 0.001026507 | IGF2/SPI1/GLIS1/SOX11/S1PR1/IRF8/KDM6B/SS18L1/AHR/NR4A1/VEGFA/EGR1/IL11/CEBPG/MAFB/HEY1/KLF2 |
| GO | GO:0005829 | cytosol | 49 | 0.001064499 | STX1A/CCL3/AIPL1/HBD/HBB/FABP4/A2M/RHOT2/CMIP/ARHGAP1/PARM1/POLH/LBP/SMOX/CFD/CACNA1A/TBC1D2/SS18L1/ARL4C/CLIP3/NPC1/FOSL1/NT5C1B/RABIF/S100A9/MRI1/S100A8/TRAF1/RGS3/PIM1/NR4A1/CEP192/NR2F2/LPL/ABCB4/BCL10/FSCN1/SOCS5/CCNB2/SGK2/PLXNA1/IRF8/AHR/TYMS/TRAPPC6B/DTYMK/AMER1/UBE2C/SYN1 |
| GO | GO:0051493 | regulation of cytoskeleton organization | 3 | 0.00108289 | CAPN6/S100A9/S100A8 |
| GO | GO:0045814 | negative regulation of gene expression, epigenetic | 3 | 0.00108289 | EZH2/SPI1/H2AFY2 |
| GO | GO:0005911 | cell-cell junction | 6 | 0.00114573 | PECAM1/FZD4/LAMA1/CFD/COL13A1/FSCN1 |
| GO | GO:0035633 | maintenance of blood-brain barrier | 3 | 0.00118234 | ITGB1/GJA1/PECAM1 |
| GO | GO:0001937 | negative regulation of endothelial cell proliferation | 3 | 0.00118234 | GJA1/APOE/NR2F2 |
| GO | GO:0004601 | peroxidase activity | 3 | 0.00118234 | HBD/GPX7/HBB |
| GO | GO:0030175 | filopodium | 4 | 0.001249672 | ITGB1/LPL/ARL4C/FSCN1 |
| GO | GO:0000981 | DNA-binding transcription factor activity, RNA polymerase II-specific | 14 | 0.001271319 | SPI1/GLIS1/SOX11/IRF8/NR4A1/NR2F2/AHR/RFX8/FOSL1/KLF2/CEBPG/MAFB/EGR1/HEY1 |
| GO | GO:0019825 | oxygen binding | 3 | 0.001287366 | HBD/CYP1B1/HBB |
| GO | GO:1903659 | regulation of complement-dependent cytotoxicity | 2 | 0.001308982 | IL11/CFH |
| GO | GO:0071813 | lipoprotein particle binding | 2 | 0.001308982 | LPL/APOE |
| GO | GO:0030183 | B cell differentiation | 4 | 0.001311877 | ITGB1/IL11/RAG1/CEBPG |
| GO | GO:0043065 | positive regulation of apoptotic process | 8 | 0.001341615 | ITGB1/CYP1B1/GZMA/FRZB/CLIP3/FOSL1/TNFRSF12A/BCL10 |
| GO | GO:0030593 | neutrophil chemotaxis | 4 | 0.001582391 | CCL18/CCL3/S100A9/S100A8 |
| GO | GO:0003677 | DNA binding | 17 | 0.001598677 | ZNF699/NR4A1/CEBPG/ZNF354B/ARID2/EGR1/H2AFY2/NPC1/ZNF469/AHR/TAF4B/RAG1/HIST1H2AI/LTF/DNASE1L3/HJURP/KLF2 |
| GO | GO:0022898 | regulation of transmembrane transporter activity | 2 | 0.00162962 | GJA1/PIM1 |
| GO | GO:0035767 | endothelial cell chemotaxis | 2 | 0.00162962 | NR4A1/VEGFA |
| GO | GO:0035425 | autocrine signaling | 2 | 0.00162962 | S100A9/S100A8 |
| GO | GO:0009629 | response to gravity | 2 | 0.00162962 | STX1A/FOSL1 |
| GO | GO:0043588 | skin development | 3 | 0.001637032 | COL5A2/COL3A1/COL1A1 |
| GO | GO:0031640 | killing of cells of other organism | 3 | 0.001637032 | DEFA4/DEFA3/LTF |
| GO | GO:0071260 | cellular response to mechanical stimulus | 4 | 0.001731199 | GJA1/IL13/BCL10/COL1A1 |
| GO | GO:0006508 | proteolysis | 9 | 0.00177124 | TLL2/CFI/CPA3/FAP/CAPN6/ADAM12/ADAMTS14/MMP9/LTF |
| GO | GO:0071347 | cellular response to interleukin-1 | 4 | 0.001809125 | CCL18/CCL3/KLF2/TFPI |
| GO | GO:0000139 | Golgi membrane | 11 | 0.001830959 | CD74/GJA1/HLA-DRA/CFD/HLA-DRB5/GOLT1B/TRAPPC6B/CHST1/GIMAP1/PARM1/NMNAT2 |
| GO | GO:0005102 | signaling receptor binding | 8 | 0.001847653 | LAMA1/APOE/ANO1/LPL/WIF1/NXPH1/A2M/NXPH4 |
| GO | GO:0009636 | response to toxic substance | 4 | 0.001972207 | AHR/TYMS/CYP1B1/CCL3 |
| GO | GO:0034371 | chylomicron remodeling | 2 | 0.001983718 | LPL/APOE |
| GO | GO:0060982 | coronary artery morphogenesis | 2 | 0.001983718 | ARID2/VEGFA |
| GO | GO:0031720 | haptoglobin binding | 2 | 0.001983718 | HBD/HBB |
| GO | GO:0034372 | very-low-density lipoprotein particle remodeling | 2 | 0.001983718 | LPL/APOE |
| GO | GO:0043184 | vascular endothelial growth factor receptor 2 binding | 2 | 0.001983718 | CADM4/VEGFA |
| GO | GO:2000343 | positive regulation of chemokine (C-X-C motif) ligand 2 production | 2 | 0.001983718 | CD74/LPL |
| GO | GO:0060907 | positive regulation of macrophage cytokine production | 2 | 0.001983718 | CD74/SPON2 |
| GO | GO:0030324 | lung development | 4 | 0.00205744 | CRISPLD2/LOX/VEGFA/FAP |
| GO | GO:0042493 | response to drug | 7 | 0.00206841 | MDK/TYMS/NPC1/LPL/FOSL1/COL1A1/LOX |
| GO | GO:0042632 | cholesterol homeostasis | 4 | 0.002145183 | FABP4/LPL/APOE/NPC1 |
| GO | GO:1902895 | positive regulation of pri-miRNA transcription by RNA polymerase II | 3 | 0.002341512 | SPI1/FOSL1/EGR1 |
| GO | GO:0031838 | haptoglobin-hemoglobin complex | 2 | 0.002370858 | HBD/HBB |
| GO | GO:0050795 | regulation of behavior | 2 | 0.002370858 | CCL3/MDK |
| GO | GO:0043177 | organic acid binding | 2 | 0.002370858 | HBD/HBB |
| GO | GO:0035999 | tetrahydrofolate interconversion | 2 | 0.002370858 | TYMS/MTHFD2 |
| GO | GO:0004888 | transmembrane signaling receptor activity | 5 | 0.002458008 | PECAM1/GABBR2/ACKR1/NPC1/TNFRSF10D |
| GO | GO:0038023 | signaling receptor activity | 6 | 0.002465863 | FZD4/PLXNA1/ACKR1/NPC1/OR3A2/FAP |
| GO | GO:0005789 | endoplasmic reticulum membrane | 14 | 0.002500118 | GJA1/CYP1B1/TMEM259/MGST2/PLOD1/TMEM129/KCNK2/ERN2/SYVN1/SPTLC2/ABHD12/PLA2G2A/B3GALTL/GIMAP1 |
| GO | GO:0019731 | antibacterial humoral response | 3 | 0.002501567 | DEFA4/DEFA3/LTF |
| GO | GO:0030574 | collagen catabolic process | 3 | 0.002668227 | COL13A1/ADAMTS14/MMP9 |
| GO | GO:0004879 | nuclear receptor activity | 3 | 0.002668227 | AHR/NR4A1/NR2F2 |
| GO | GO:0032570 | response to progesterone | 3 | 0.002668227 | TYMS/FOSL1/CSN1S1 |
| GO | GO:0007275 | multicellular organism development | 9 | 0.002669662 | DCHS1/IGF2/TLL2/PIM1/PLXNA1/ANO1/WIF1/TDRD12/EDIL3 |
| GO | GO:0050786 | RAGE receptor binding | 2 | 0.002790625 | S100A9/S100A8 |
| GO | GO:0002523 | leukocyte migration involved in inflammatory response | 2 | 0.002790625 | S100A9/S100A8 |
| GO | GO:0048407 | platelet-derived growth factor binding | 2 | 0.002790625 | COL3A1/COL1A1 |
| GO | GO:0006707 | cholesterol catabolic process | 2 | 0.002790625 | FAP/APOE |
| GO | GO:0048333 | mesodermal cell differentiation | 2 | 0.002790625 | ITGB1/KDM6B |
| GO | GO:0045446 | endothelial cell differentiation | 2 | 0.002790625 | S1PR1/KDM6B |
| GO | GO:0005833 | hemoglobin complex | 2 | 0.002790625 | HBD/HBB |
| GO | GO:0008528 | G protein-coupled peptide receptor activity | 3 | 0.002841582 | GCGR/CRHR2/CALCRL |
| GO | GO:0045087 | innate immune response | 10 | 0.002844844 | SPON2/CFI/C1QC/C1QB/C1QA/DEFA4/S100A9/S100A8/IGLL5/BCL10 |
| GO | GO:0006956 | complement activation | 4 | 0.002941959 | CFH/C1QC/C1QB/C1QA |
| GO | GO:0002224 | toll-like receptor signaling pathway | 3 | 0.003021718 | S100A9/BCL10/S100A8 |
| GO | GO:0001932 | regulation of protein phosphorylation | 3 | 0.003021718 | LOX/CADM4/ROPN1L |
| GO | GO:0048538 | thymus development | 3 | 0.003021718 | CCNB2/MAFB/RAG1 |
| GO | GO:0034097 | response to cytokine | 3 | 0.003021718 | TYMS/FOSL1/COL3A1 |
| GO | GO:0032588 | trans-Golgi network membrane | 4 | 0.003053958 | CD74/CLIP3/HLA-DRA/HLA-DRB5 |
| GO | GO:0006887 | exocytosis | 4 | 0.003053958 | STX1A/CCL3/SLC17A9/GCGR |
| GO | GO:0005604 | basement membrane | 4 | 0.003053958 | LAMA1/LOXL2/HMCN1/LOXL1 |
| GO | GO:0048856 | anatomical structure development | 3 | 0.003208721 | WIF1/SPARCL1/NR2F2 |
| GO | GO:0006958 | complement activation, classical pathway | 5 | 0.003238259 | C1QC/IGLL5/C1QB/C1QA/CFI |
| GO | GO:0050927 | positive regulation of positive chemotaxis | 2 | 0.003242607 | S1PR1/VEGFA |
| GO | GO:0060674 | placenta blood vessel development | 2 | 0.003242607 | FOSL1/NR2F2 |
| GO | GO:0071803 | positive regulation of podosome assembly | 2 | 0.003242607 | LPL/FSCN1 |
| GO | GO:0045080 | - | 2 | 0.003242607 | CD74/EGR1 |
| GO | GO:2000648 | positive regulation of stem cell proliferation | 2 | 0.003242607 | SOX11/GJA1 |
| GO | GO:0033690 | positive regulation of osteoblast proliferation | 2 | 0.003242607 | LTF/CTHRC1 |
| GO | GO:0050728 | negative regulation of inflammatory response | 4 | 0.00328662 | SOCS5/IL13/APOE/CALCRL |
| GO | GO:0003151 | outflow tract morphogenesis | 3 | 0.003402671 | SOX11/NPY1R/VEGFA |
| GO | GO:0032587 | ruffle membrane | 4 | 0.003407353 | ITGB1/MTSS1L/FAP/LPL |
| GO | GO:0051092 | positive regulation of NF-kappaB transcription factor activity | 5 | 0.003503556 | TRAF1/S100A9/LTF/S100A8/BCL10 |
| GO | GO:0043312 | neutrophil degranulation | 9 | 0.003508152 | CRISPLD2/PECAM1/SLC2A5/HBB/DEFA4/S100A9/S100A8/MMP9/LTF |
| GO | GO:0001666 | response to hypoxia | 5 | 0.003595357 | POSTN/LOXL2/EGR1/VEGFA/PLOD1 |
| GO | GO:0051781 | positive regulation of cell division | 3 | 0.003603649 | IGF2/MDK/VEGFA |
| GO | GO:0001726 | ruffle | 4 | 0.003657801 | ITGB1/LPL/TNFRSF12A/FSCN1 |
| GO | GO:0019886 | antigen processing and presentation of exogenous peptide antigen via MHC class II | 4 | 0.003657801 | CD74/HLA-DRA/CFD/HLA-DRB5 |
| GO | GO:0016064 | immunoglobulin mediated immune response | 2 | 0.003726398 | CD74/BCL10 |
| GO | GO:0071383 | cellular response to steroid hormone stimulus | 2 | 0.003726398 | NPC1/TFPI |
| GO | GO:0005344 | oxygen carrier activity | 2 | 0.003726398 | HBD/HBB |
| GO | GO:0030195 | negative regulation of blood coagulation | 2 | 0.003726398 | APOE/TFPI |
| GO | GO:0042627 | chylomicron | 2 | 0.003726398 | LPL/APOE |
| GO | GO:2000678 | negative regulation of transcription regulatory region DNA binding | 2 | 0.003726398 | SOX11/HEY1 |
| GO | GO:0042542 | response to hydrogen peroxide | 3 | 0.003811733 | COL1A1/FOSL1/HBB |
| GO | GO:0008253 | 5'-nucleotidase activity | 2 | 0.004241593 | NT5DC2/NT5C1B |
| GO | GO:0010996 | response to auditory stimulus | 2 | 0.004241593 | MDK/ABHD12 |
| GO | GO:2000279 | negative regulation of DNA biosynthetic process | 2 | 0.004241593 | GJA1/KCNK2 |
| GO | GO:0042043 | neurexin family protein binding | 2 | 0.004241593 | FAP/CPE |
| GO | GO:1904645 | response to amyloid-beta | 2 | 0.004241593 | CACNA1A/MMP9 |
| GO | GO:0015671 | oxygen transport | 2 | 0.004241593 | HBD/HBB |
| GO | GO:0071902 | positive regulation of protein serine/threonine kinase activity | 3 | 0.004249515 | IGF2/EZH2/LTF |
| GO | GO:0071230 | cellular response to amino acid stimulus | 3 | 0.004479357 | COL5A2/COL3A1/COL1A1 |
| GO | GO:0004252 | serine-type endopeptidase activity | 5 | 0.004500803 | FAP/GZMA/CFI/LTF/MMP9 |
| GO | GO:0008134 | transcription factor binding | 7 | 0.004603077 | SPI1/CEBPG/PIM1/AHR/MAFB/BCL10/HEY1 |
| GO | GO:0045600 | positive regulation of fat cell differentiation | 3 | 0.004716593 | FRZB/LPL/WIF1 |
| GO | GO:0004222 | metalloendopeptidase activity | 4 | 0.00478436 | ADAM12/ADAMTS14/TLL2/MMP9 |
| GO | GO:1901653 | cellular response to peptide | 2 | 0.004787793 | ANO1/KLF2 |
| GO | GO:0016298 | lipase activity | 2 | 0.004787793 | FAP/LPL |
| GO | GO:0060044 | negative regulation of cardiac muscle cell proliferation | 2 | 0.004787793 | GJA1/KCNK2 |
| GO | GO:0030970 | retrograde protein transport, ER to cytosol | 2 | 0.004787793 | SYVN1/TMEM129 |
| GO | GO:0071682 | endocytic vesicle lumen | 2 | 0.004787793 | APOE/HBB |
| GO | GO:0006968 | cellular defense response | 3 | 0.004961289 | ITGB1/FOSL1/BCL10 |
| GO | GO:0012507 | ER to Golgi transport vesicle membrane | 3 | 0.004961289 | CD74/HLA-DRA/HLA-DRB5 |
| GO | GO:0003700 | DNA-binding transcription factor activity | 10 | 0.005064327 | SPI1/RFX8/SOX11/EGR1/NR2F2/AHR/FOSL1/KLF2/MAFB/HEY1 |
| GO | GO:0023026 | MHC class II protein complex binding | 2 | 0.005364602 | CD74/HLA-DRA |
| GO | GO:1905564 | positive regulation of vascular endothelial cell proliferation | 2 | 0.005364602 | IGF2/MDK |
| GO | GO:0034374 | low-density lipoprotein particle remodeling | 2 | 0.005364602 | PLA2G2A/APOE |
| GO | GO:0046889 | positive regulation of lipid biosynthetic process | 2 | 0.005364602 | APOE/SIRT4 |
| GO | GO:0044849 | estrous cycle | 2 | 0.005364602 | MDK/EGR1 |
| GO | GO:0015909 | long-chain fatty acid transport | 2 | 0.005364602 | FABP4/APOE |
| GO | GO:0030502 | negative regulation of bone mineralization | 2 | 0.005364602 | SRGN/CCL3 |
| GO | GO:0036498 | IRE1-mediated unfolded protein response | 3 | 0.005473317 | SYVN1/ERN2/TATDN2 |
| GO | GO:1903561 | extracellular vesicle | 3 | 0.005473317 | COL6A3/APOE/EDIL3 |
| GO | GO:1904724 | tertiary granule lumen | 3 | 0.005473317 | HBB/LTF/MMP9 |
| GO | GO:0005507 | copper ion binding | 3 | 0.005473317 | LOXL1/LOX/LOXL2 |
| GO | GO:0006955 | immune response | 8 | 0.005679627 | HLA-DRA/IL13/CEBPG/GZMA/CCL18/IRF8/C1QC/RAG1 |
| GO | GO:0010812 | negative regulation of cell-substrate adhesion | 2 | 0.005971627 | FZD4/COL1A1 |
| GO | GO:0055114 | oxidation-reduction process | 9 | 0.006006558 | LOXL1/CYP1B1/MTHFD2/PLOD1/MGST2/NCF1B/GPX7/KDM6B/SMOX |
| GO | GO:0006644 | phospholipid metabolic process | 3 | 0.006015935 | PLA2G2A/LPL/PRG4 |
| GO | GO:0032731 | positive regulation of interleukin-1 beta production | 3 | 0.006015935 | CCL3/LPL/EGR1 |
| GO | GO:0048661 | positive regulation of smooth muscle cell proliferation | 3 | 0.00629886 | S1PR1/IL13/CALCRL |
| GO | GO:0005764 | lysosome | 6 | 0.006413373 | GJA1/HLA-DRA/CALCRL/FAP/NPC1/BCL10 |
| GO | GO:0043123 | positive regulation of I-kappaB kinase/NF-kappaB signaling | 5 | 0.006492988 | CD74/GOLT1B/GJA1/LTF/BCL10 |
| GO | GO:0050839 | cell adhesion molecule binding | 3 | 0.006589602 | ITGB1/POSTN/CPE |
| GO | GO:0019835 | cytolysis | 2 | 0.00660848 | PLA2G2A/GZMA |
| GO | GO:0010744 | positive regulation of macrophage derived foam cell differentiation | 2 | 0.00660848 | PLA2G2A/LPL |
| GO | GO:0016209 | antioxidant activity | 2 | 0.00660848 | APOE/S100A9 |
| GO | GO:0044877 | protein-containing complex binding | 7 | 0.006669296 | ITGB1/STX1A/APOE/CEBPG/FZD4/MAFB/BCL10 |
| GO | GO:0045669 | positive regulation of osteoblast differentiation | 3 | 0.006888214 | SOX11/LTF/CTHRC1 |
| GO | GO:0050804 | modulation of chemical synaptic transmission | 3 | 0.006888214 | SRGN/CACNA1A/FAP |
| GO | GO:0006909 | phagocytosis | 3 | 0.006888214 | ITGB1/PECAM1/IRF8 |
| GO | GO:0016055 | Wnt signaling pathway | 5 | 0.007074183 | AMER1/WIF1/FZD4/CPE/NARF |
| GO | GO:0006935 | chemotaxis | 4 | 0.007075065 | CCL18/CCL3/S1PR1/FOSL1 |
| GO | GO:0098978 | glutamatergic synapse | 7 | 0.007174768 | ITGB1/STX1A/APOE/FZD4/CACNA1A/SPARCL1/SRGN |
| GO | GO:0007188 | adenylate cyclase-modulating G protein-coupled receptor signaling pathway | 3 | 0.007194747 | GCGR/CRHR2/CALCRL |
| GO | GO:0040008 | regulation of growth | 3 | 0.007194747 | SOCS5/CCNB2/SEPP1 |
| GO | GO:0043254 | regulation of protein-containing complex assembly | 2 | 0.007274774 | APOE/HJURP |
| GO | GO:0032332 | positive regulation of chondrocyte differentiation | 2 | 0.007274774 | LOXL2/GDF6 |
| GO | GO:0031902 | late endosome membrane | 4 | 0.007481603 | HLA-DRA/CFD/NPC1/HLA-DRB5 |
| GO | GO:0050900 | leukocyte migration | 5 | 0.007533257 | ITGB1/CD74/COL1A1/TNFRSF10D/PECAM1 |
| GO | GO:0006915 | apoptotic process | 9 | 0.007640967 | TRAF1/GZMA/PIM1/GDF6/AHR/S100A9/S100A8/TNFRSF10D/GJA1 |
| GO | GO:0004602 | glutathione peroxidase activity | 2 | 0.007970128 | MGST2/GPX7 |
| GO | GO:0034361 | very-low-density lipoprotein particle | 2 | 0.007970128 | LPL/APOE |
| GO | GO:1990837 | sequence-specific double-stranded DNA binding | 9 | 0.00825506 | GLIS1/CEBPG/IRF8/NR4A1/AHR/FOSL1/EGR1/HEY1/KLF2 |
| GO | GO:0004620 | phospholipase activity | 2 | 0.008694164 | LPL/ABHD12 |
| GO | GO:0071542 | dopaminergic neuron differentiation | 2 | 0.008694164 | CDNF/VEGFA |
| GO | GO:0045807 | positive regulation of endocytosis | 2 | 0.008694164 | CLIP3/APOE |
| GO | GO:0010667 | negative regulation of cardiac muscle cell apoptotic process | 2 | 0.008694164 | MDK/SIRT4 |
| GO | GO:0055007 | cardiac muscle cell differentiation | 2 | 0.008694164 | ITGB1/KDM6B |
| GO | GO:0035567 | non-canonical Wnt signaling pathway | 2 | 0.008694164 | FRZB/FZD4 |
| GO | GO:1990138 | neuron projection extension | 2 | 0.008694164 | POSTN/PLXNA1 |
| GO | GO:0031490 | chromatin DNA binding | 3 | 0.008847874 | EZH2/H2AFY2/KDM6B |
| GO | GO:0030666 | endocytic vesicle membrane | 3 | 0.008847874 | CD74/HLA-DRA/HLA-DRB5 |
| GO | GO:0008217 | regulation of blood pressure | 3 | 0.008847874 | GCGR/NPY1R/HBB |
| GO | GO:0070098 | chemokine-mediated signaling pathway | 3 | 0.009202897 | CCL18/CCL3/ACKR1 |
| GO | GO:0005518 | collagen binding | 3 | 0.009202897 | LOX/SPARCL1/MMP9 |
| GO | GO:0009749 | response to glucose | 3 | 0.009202897 | GJA1/LPL/EGR1 |
| GO | GO:0001228 | DNA-binding transcription activator activity, RNA polymerase II-specific | 8 | 0.009287029 | SPI1/CEBPG/SOX11/GLIS1/NR4A1/FOSL1/EGR1/MAFB |
| GO | GO:0010596 | negative regulation of endothelial cell migration | 2 | 0.009446508 | APOE/NR2F2 |
| GO | GO:0007166 | cell surface receptor signaling pathway | 6 | 0.009562178 | PECAM1/LAMA1/CALCRL/TNFRSF10D/GCGR/CRHR2 |
| GO | GO:0042531 | positive regulation of tyrosine phosphorylation of STAT protein | 3 | 0.009566146 | PECAM1/IL13/VEGFA |
| GO | GO:0045766 | positive regulation of angiogenesis | 4 | 0.009745058 | ITGB1/VEGFA/ADAM12/CYP1B1 |
| GO | GO:0007601 | visual perception | 5 | 0.00995173 | EFEMP1/HMCN1/CYP1B1/AIPL1/COL1A1 |
| GO | GO:0070374 | positive regulation of ERK1 and ERK2 cascade | 5 | 0.010142588 | CD74/CCL3/APOE/CCL18/PLA2G2A |
| GO | GO:0009314 | response to radiation | 2 | 0.010226787 | COL3A1/POLH |
| GO | GO:0017015 | regulation of transforming growth factor beta receptor signaling pathway | 2 | 0.010226787 | SOX11/LOX |
| GO | GO:0033344 | cholesterol efflux | 2 | 0.010226787 | NPC1/APOE |
| GO | GO:0048844 | artery morphogenesis | 2 | 0.010226787 | APOE/VEGFA |
| GO | GO:0090398 | cellular senescence | 2 | 0.010226787 | ULK3/C2orf40 |
| GO | GO:0016125 | sterol metabolic process | 2 | 0.010226787 | FAP/CYP1B1 |
| GO | GO:0043433 | negative regulation of DNA-binding transcription factor activity | 3 | 0.010705605 | EZH2/CEBPG/PIM1 |
| GO | GO:0060333 | interferon-gamma-mediated signaling pathway | 3 | 0.010705605 | HLA-DRA/IRF8/HLA-DRB5 |
| GO | GO:0019882 | antigen processing and presentation | 2 | 0.011034635 | CD74/HLA-DRA |
| GO | GO:0090103 | cochlea morphogenesis | 2 | 0.011034635 | FRZB/CTHRC1 |
| GO | GO:0046686 | response to cadmium ion | 2 | 0.011034635 | NPC1/DTYMK |
| GO | GO:0034383 | low-density lipoprotein particle clearance | 2 | 0.011034635 | FAP/NPC1 |
| GO | GO:0007165 | signal transduction | 13 | 0.011240135 | PECAM1/RGS3/RASL12/GJA1/CCL18/NR4A1/SPARCL1/PRG4/WIF1/OR3A2/TNFRSF10D/MDK/CDNF |
| GO | GO:0006325 | chromatin organization | 4 | 0.011295256 | HIST1H2AI/EZH2/H2AFY2/SS18L1 |
| GO | GO:0045671 | negative regulation of osteoclast differentiation | 2 | 0.011869687 | CCL3/MAFB |
| GO | GO:0043236 | laminin binding | 2 | 0.011869687 | ITGB1/LBP |
| GO | GO:0006629 | lipid metabolic process | 4 | 0.012126471 | MGST2/FAP/TPRA1/ABCB4 |
| GO | GO:0006816 | calcium ion transport | 3 | 0.012342081 | CACNA1A/CALCRL/CCL3 |
| GO | GO:0015949 | nucleobase-containing small molecule interconversion | 2 | 0.012731581 | TYMS/DTYMK |
| GO | GO:2001238 | positive regulation of extrinsic apoptotic signaling pathway | 2 | 0.012731581 | TNFRSF12A/BCL10 |
| GO | GO:0090023 | positive regulation of neutrophil chemotaxis | 2 | 0.012731581 | CD74/MDK |
| GO | GO:0019433 | triglyceride catabolic process | 2 | 0.012731581 | FABP4/LPL |
| GO | GO:0017025 | TBP-class protein binding | 2 | 0.012731581 | AHR/TAF13 |
| GO | GO:1900745 | positive regulation of p38MAPK cascade | 2 | 0.012731581 | VEGFA/GDF6 |
| GO | GO:0006259 | DNA metabolic process | 2 | 0.012731581 | DNASE1L3/TATDN2 |
| GO | GO:0004806 | triglyceride lipase activity | 2 | 0.012731581 | FAP/LPL |
| GO | GO:0045296 | cadherin binding | 6 | 0.012738352 | DCHS1/ITGB1/CCNB2/TBC1D2/ARHGAP1/FSCN1 |
| GO | GO:0001618 | virus receptor activity | 3 | 0.012772319 | ITGB1/LBP/NPC1 |
| GO | GO:0030154 | cell differentiation | 9 | 0.012887499 | MDK/TLL2/SPI1/SOX11/NR2F2/COL13A1/TMEM176B/TDRD12/HEY1 |
| GO | GO:0001503 | ossification | 3 | 0.013211055 | COL5A2/LTF/COL1A1 |
| GO | GO:0071897 | DNA biosynthetic process | 2 | 0.013619962 | TYMS/LOX |
| GO | GO:0007628 | adult walking behavior | 2 | 0.013619962 | NPC1/ABHD12 |
| GO | GO:0001968 | fibronectin binding | 2 | 0.013619962 | ITGB1/VEGFA |
| GO | GO:0042744 | hydrogen peroxide catabolic process | 2 | 0.013619962 | HBD/HBB |
| GO | GO:0035924 | cellular response to vascular endothelial growth factor stimulus | 2 | 0.013619962 | NR4A1/VEGFA |
| GO | GO:0007267 | cell-cell signaling | 5 | 0.01378104 | CCL18/GJA1/S100A9/C1QA/CCL3 |
| GO | GO:0043565 | sequence-specific DNA binding | 7 | 0.014076509 | EGR1/CEBPG/NR4A1/NR2F2/RAG1/KDM6B/MAFB |
| GO | GO:0016042 | lipid catabolic process | 3 | 0.014114109 | PLA2G2A/FAP/LPL |
| GO | GO:0005667 | transcription regulator complex | 5 | 0.014259791 | AHR/SOX11/MAFB/SPI1/NR4A1 |
| GO | GO:0050840 | extracellular matrix binding | 2 | 0.014534473 | VEGFA/SPARCL1 |
| GO | GO:0070328 | triglyceride homeostasis | 2 | 0.014534473 | LPL/APOE |
| GO | GO:0001774 | microglial cell activation | 2 | 0.014534473 | IL13/C1QA |
| GO | GO:0051603 | proteolysis involved in cellular protein catabolic process | 2 | 0.014534473 | FAP/GZMA |
| GO | GO:0048020 | CCR chemokine receptor binding | 2 | 0.014534473 | CCL18/CCL3 |
| GO | GO:0002102 | podosome | 2 | 0.014534473 | LPL/FSCN1 |
| GO | GO:0043029 | T cell homeostasis | 2 | 0.014534473 | CCNB2/RAG1 |
| GO | GO:0050829 | defense response to Gram-negative bacterium | 3 | 0.015051407 | DEFA4/DEFA3/LTF |
| GO | GO:0006464 | cellular protein modification process | 3 | 0.015051407 | LOX/CPE/LOXL2 |
| GO | GO:0000902 | cell morphogenesis | 3 | 0.015051407 | DCHS1/FAP/KLF2 |
| GO | GO:0001958 | endochondral ossification | 2 | 0.015474765 | COL13A1/COL1A1 |
| GO | GO:0010803 | regulation of tumor necrosis factor-mediated signaling pathway | 2 | 0.015474765 | CLIP3/TRAF1 |
| GO | GO:0004181 | metallocarboxypeptidase activity | 2 | 0.015474765 | CPA3/CPE |
| GO | GO:0048565 | digestive tract development | 2 | 0.015474765 | DCHS1/COL3A1 |
| GO | GO:0051281 | positive regulation of release of sequestered calcium ion into cytosol | 2 | 0.015474765 | IL13/CEMIP |
| GO | GO:0007159 | leukocyte cell-cell adhesion | 2 | 0.015474765 | ITGB1/PECAM1 |
| GO | GO:0007420 | brain development | 5 | 0.015505251 | TSPAN2/CACNA1A/S1PR1/SEPP1/H2AFY2 |
| GO | GO:0043085 | positive regulation of catalytic activity | 3 | 0.015532942 | IGF2/MGST2/NCF1B |
| GO | GO:0016477 | cell migration | 5 | 0.015762773 | ITGB1/S1PR1/LPL/CTHRC1/FSCN1 |
| GO | GO:0043539 | protein serine/threonine kinase activator activity | 2 | 0.016440489 | IGF2/LTF |
| GO | GO:0043388 | positive regulation of DNA binding | 2 | 0.016440489 | CEBPG/MMP9 |
| GO | GO:0060612 | adipose tissue development | 2 | 0.016440489 | AMER1/SPTLC2 |
| GO | GO:0014047 | glutamate secretion | 2 | 0.016440489 | STX1A/GJA1 |
| GO | GO:0000132 | establishment of mitotic spindle orientation | 2 | 0.016440489 | ITGB1/GJA1 |
| GO | GO:0098685 | Schaffer collateral - CA1 synapse | 3 | 0.016521862 | ITGB1/SRGN/SYN1 |
| GO | GO:0000977 | RNA polymerase II transcription regulatory region sequence-specific DNA binding | 6 | 0.017264997 | EGR1/GLIS1/H2AFY2/NR4A1/NR2F2/HEY1 |
| GO | GO:0060325 | face morphogenesis | 2 | 0.017431302 | CRISPLD2/COL1A1 |
| GO | GO:0033077 | T cell differentiation in thymus | 2 | 0.017431302 | MAFB/RAG1 |
| GO | GO:0051059 | NF-kappaB binding | 2 | 0.017431302 | TAF4B/BCL10 |
| GO | GO:2000352 | negative regulation of endothelial cell apoptotic process | 2 | 0.017431302 | IL11/IL13 |
| GO | GO:0042605 | peptide antigen binding | 2 | 0.017431302 | HLA-DRA/HLA-DRB5 |
| GO | GO:0090314 | positive regulation of protein targeting to membrane | 2 | 0.017431302 | CACNA1A/CEMIP |
| GO | GO:0000777 | condensed chromosome kinetochore | 3 | 0.017545337 | CAMP/HJURP/SS18L1 |
| GO | GO:0008013 | beta-catenin binding | 3 | 0.017545337 | GJA1/KDM6B/AMER1 |
| GO | GO:0097421 | liver regeneration | 2 | 0.018446862 | TYMS/EZH2 |
| GO | GO:0048247 | lymphocyte chemotaxis | 2 | 0.018446862 | CCL18/CCL3 |
| GO | GO:0002040 | sprouting angiogenesis | 2 | 0.018446862 | LOXL2/VEGFA |
| GO | GO:0090102 | cochlea development | 2 | 0.018446862 | DCHS1/KCNK2 |
| GO | GO:0048477 | oogenesis | 2 | 0.018446862 | MDK/TAF4B |
| GO | GO:0016020 | membrane | 21 | 0.019027905 | DCHS1/ITGB1/LOXL2/APOE/CCNB2/FRZB/RABIF/MGST2/CFI/LAMA1/SYVN1/GOLT1B/NPC1/FUT4/VEGFA/LBP/RHOT2/TM9SF2/ABCB4/CD74/ABHD12 |
| GO | GO:0046718 | viral entry into host cell | 3 | 0.019145526 | ITGB1/LBP/NPC1 |
| GO | GO:0007156 | homophilic cell adhesion via plasma membrane adhesion molecules | 4 | 0.019424452 | DCHS1/ITGB1/HMCN1/PECAM1 |
| GO | GO:0005575 | cellular_component | 7 | 0.019429324 | FIBIN/ETNPPL/GPATCH8/TDRD12/CAMK2N1/NXPH4/S100A2 |
| GO | GO:0042056 | chemoattractant activity | 2 | 0.019486832 | CCL3/VEGFA |
| GO | GO:0001530 | lipopolysaccharide binding | 2 | 0.019486832 | SPON2/LTF |
| GO | GO:0030032 | lamellipodium assembly | 2 | 0.019486832 | ITGB1/S1PR1 |
| GO | GO:0032839 | dendrite cytoplasm | 2 | 0.019486832 | CACNA1A/ABHD12 |
| GO | GO:0004519 | endonuclease activity | 2 | 0.019486832 | RAG1/ERN2 |
| GO | GO:0006641 | triglyceride metabolic process | 2 | 0.019486832 | LPL/APOE |
| GO | GO:0045892 | negative regulation of transcription, DNA-templated | 8 | 0.019687621 | LOXL2/SPI1/NR2F2/AHR/FABP4/EZH2/ERN2/HEY1 |
| GO | GO:0032755 | positive regulation of interleukin-6 production | 3 | 0.019696281 | CD74/SPON2/LPL |
| GO | GO:0007568 | aging | 4 | 0.019808264 | TYMS/LOXL2/TPRA1/C1QA |
| GO | GO:0006805 | xenobiotic metabolic process | 3 | 0.020255726 | AHR/MGST2/CYP1B1 |
| GO | GO:0034504 | protein localization to nucleus | 2 | 0.020550876 | CFD/COL1A1 |
| GO | GO:1904707 | positive regulation of vascular associated smooth muscle cell proliferation | 2 | 0.020550876 | GJA1/MMP9 |
| GO | GO:0005154 | epidermal growth factor receptor binding | 2 | 0.020550876 | EFEMP1/SOCS5 |
| GO | GO:0010942 | positive regulation of cell death | 2 | 0.020550876 | KCNK2/HBB |
| GO | GO:0031663 | lipopolysaccharide-mediated signaling pathway | 2 | 0.020550876 | CCL3/BCL10 |
| GO | GO:0060412 | ventricular septum morphogenesis | 2 | 0.020550876 | SOX11/HEY1 |
| GO | GO:0098794 | postsynapse | 3 | 0.021400701 | C1QC/C1QB/C1QA |
| GO | GO:0007043 | cell-cell junction assembly | 2 | 0.021638663 | DCHS1/FSCN1 |
| GO | GO:2001244 | positive regulation of intrinsic apoptotic signaling pathway | 2 | 0.021638663 | S100A9/S100A8 |
| GO | GO:0051017 | actin filament bundle assembly | 2 | 0.021638663 | LPL/FSCN1 |
| GO | GO:0000978 | RNA polymerase II cis-regulatory region sequence-specific DNA binding | 9 | 0.022267271 | SPI1/RFX8/SOX11/IRF8/NR4A1/EZH2/FOSL1/KDM6B/MAFB |
| GO | GO:0071346 | cellular response to interferon-gamma | 3 | 0.022580469 | CCL18/CCL3/IRF8 |
| GO | GO:0098742 | cell-cell adhesion via plasma-membrane adhesion molecules | 2 | 0.022749865 | DCHS1/PECAM1 |
| GO | GO:0006730 | one-carbon metabolic process | 2 | 0.022749865 | MTHFD2/CA12 |
| GO | GO:0008219 | cell death | 2 | 0.022749865 | CACNA1A/BCL10 |
| GO | GO:0032735 | positive regulation of interleukin-12 production | 2 | 0.022749865 | MDK/IRF8 |
| GO | GO:0048018 | receptor ligand activity | 2 | 0.022749865 | IGF2/VEGFA |
| GO | GO:0007160 | cell-matrix adhesion | 3 | 0.023183402 | ITGB1/COL3A1/COL13A1 |
| GO | GO:0005794 | Golgi apparatus | 12 | 0.023354076 | GJA1/APOE/CPE/SRGN/ITM2A/FUT4/NPC1/COL1A1/NMNAT2/FIBIN/PARM1/SYN1 |
| GO | GO:0042383 | sarcolemma | 3 | 0.023795032 | ITGB1/COL6A3/SLC2A5 |
| GO | GO:0019900 | kinase binding | 3 | 0.023795032 | STX1A/CACNA1A/BCL10 |
| GO | GO:0050830 | defense response to Gram-positive bacterium | 3 | 0.023795032 | PLA2G2A/DEFA4/DEFA3 |
| GO | GO:0048488 | synaptic vesicle endocytosis | 2 | 0.023884156 | STX1A/FAP |
| GO | GO:0016251 | RNA polymerase II general transcription initiation factor activity | 2 | 0.023884156 | TAF13/TAF4B |
| GO | GO:0007596 | blood coagulation | 4 | 0.02390455 | COL1A1/HBB/HBD/TFPI |
| GO | GO:0016324 | apical plasma membrane | 6 | 0.024196703 | GJA1/SLC2A5/ANO1/KCNK2/ABCB4/C2orf40 |
| GO | GO:0004867 | serine-type endopeptidase inhibitor activity | 3 | 0.024415358 | COL6A3/A2M/TFPI |
| GO | GO:0045471 | response to ethanol | 3 | 0.024415358 | TYMS/IL13/S100A8 |
| GO | GO:0016032 | viral process | 7 | 0.024782358 | HLA-DRA/ZC3H7B/CFH/CFI/C1QA/FAM46C/TSPAN7 |
| GO | GO:1901216 | positive regulation of neuron death | 2 | 0.025041214 | EGR1/C1QA |
| GO | GO:0043491 | protein kinase B signaling | 2 | 0.025041214 | CCL3/LOX |
| GO | GO:0000976 | transcription regulatory region sequence-specific DNA binding | 5 | 0.025210755 | AHR/SOX11/EGR1/H2AFY2/KLF2 |
| GO | GO:0009566 | fertilization | 2 | 0.026220719 | TDRD12/NR2F2 |
| GO | GO:0045429 | positive regulation of nitric oxide biosynthetic process | 2 | 0.026220719 | KLF2/HBB |
| GO | GO:0009968 | negative regulation of signal transduction | 2 | 0.026220719 | SOCS5/RGS5 |
| GO | GO:0003007 | heart morphogenesis | 2 | 0.026220719 | ARID2/VEGFA |
| GO | GO:0003779 | actin binding | 5 | 0.026281853 | ITGB1/CACNA1A/LPL/FSCN1/SYN1 |
| GO | GO:0006979 | response to oxidative stress | 3 | 0.026328463 | SEPP1/SGK2/GPX7 |
| GO | GO:0005096 | GTPase activator activity | 5 | 0.027011839 | MTSS1L/RGS5/TBC1D2/ARHGAP1/RGS3 |
| GO | GO:0001772 | immunological synapse | 2 | 0.027422357 | GZMA/BCL10 |
| GO | GO:0032715 | negative regulation of interleukin-6 production | 2 | 0.027422357 | SOCS5/KLF2 |
| GO | GO:0004866 | endopeptidase inhibitor activity | 2 | 0.027422357 | A2M/TFPI |
| GO | GO:0017046 | peptide hormone binding | 2 | 0.027422357 | GCGR/CRHR2 |
| GO | GO:0006342 | chromatin silencing | 2 | 0.027422357 | HIST1H2AI/H2AFY2 |
| GO | GO:0015701 | bicarbonate transport | 2 | 0.028645813 | CA12/HBB |
| GO | GO:0030890 | positive regulation of B cell proliferation | 2 | 0.028645813 | CD74/IL13 |
| GO | GO:0001895 | retina homeostasis | 2 | 0.028645813 | LTF/AIPL1 |
| GO | GO:0019955 | cytokine binding | 2 | 0.028645813 | CD74/FZD4 |
| GO | GO:2000134 | negative regulation of G1/S transition of mitotic cell cycle | 2 | 0.028645813 | WEE1/EZH2 |
| GO | GO:1904646 | cellular response to amyloid-beta | 2 | 0.028645813 | GJA1/CACNA1A |
| GO | GO:0001568 | blood vessel development | 2 | 0.028645813 | AHR/COL1A1 |
| GO | GO:0051591 | response to cAMP | 2 | 0.028645813 | FOSL1/COL1A1 |
| GO | GO:0005759 | mitochondrial matrix | 6 | 0.029077164 | MTHFD2/SIRT4/ACSS3/ETNPPL/TYMS/DTYMK |
| GO | GO:0030027 | lamellipodium | 4 | 0.029450955 | ITGB1/MTSS1L/FAP/FSCN1 |
| GO | GO:0031623 | receptor internalization | 2 | 0.029890777 | ITGB1/CALCRL |
| GO | GO:0043010 | camera-type eye development | 2 | 0.029890777 | EFEMP1/LAMA1 |
| GO | GO:0005771 | multivesicular body | 2 | 0.029890777 | CD74/GJA1 |
| GO | GO:0045786 | negative regulation of cell cycle | 2 | 0.029890777 | CAMK2N1/NR4A1 |
| GO | GO:0005125 | cytokine activity | 4 | 0.029944675 | IL11/IL13/VEGFA/GDF6 |
| GO | GO:0007218 | neuropeptide signaling pathway | 3 | 0.030388637 | NPY1R/CPE/NXPH4 |
| GO | GO:0050918 | positive chemotaxis | 2 | 0.031156942 | CCL3/VEGFA |
| GO | GO:0002548 | monocyte chemotaxis | 2 | 0.031156942 | CCL18/CCL3 |
| GO | GO:0008277 | regulation of G protein-coupled receptor signaling pathway | 2 | 0.031156942 | RGS5/RGS3 |
| GO | GO:0007507 | heart development | 4 | 0.031455152 | COL3A1/GJA1/LOX/CALCRL |
| GO | GO:0043410 | positive regulation of MAPK cascade | 3 | 0.031811064 | CD74/IL11/IGF2 |
| GO | GO:0030054 | cell junction | 4 | 0.031968436 | GJA1/TBC1D2/LPL/S100A9 |
| GO | GO:0005669 | transcription factor TFIID complex | 2 | 0.032444004 | TAF13/TAF4B |
| GO | GO:0007162 | negative regulation of cell adhesion | 2 | 0.032444004 | MDK/PLXNA1 |
| GO | GO:0003824 | catalytic activity | 2 | 0.032444004 | FAP/HHIPL2 |
| GO | GO:0010976 | positive regulation of neuron projection development | 3 | 0.03326784 | MDK/APOE/S100A9 |
| GO | GO:0043066 | negative regulation of apoptotic process | 7 | 0.033487816 | CD74/AIPL1/PIM1/MMP9/VEGFA/LTF/TNFRSF10D |
| GO | GO:0071407 | cellular response to organic cyclic compound | 2 | 0.033751661 | CCL3/CYP1B1 |
| GO | GO:0035914 | skeletal muscle cell differentiation | 2 | 0.033751661 | SOX11/EGR1 |
| GO | GO:0005044 | scavenger receptor activity | 2 | 0.033751661 | LOXL2/CFI |
| GO | GO:0051091 | positive regulation of DNA-binding transcription factor activity | 3 | 0.03400907 | FOSL1/CEBPG/FZD4 |
| GO | GO:0007189 | adenylate cyclase-activating G protein-coupled receptor signaling pathway | 3 | 0.03475884 | GCGR/S1PR1/CALCRL |
| GO | GO:0048839 | inner ear development | 2 | 0.035079615 | CACNA1A/C1QB |
| GO | GO:0009755 | hormone-mediated signaling pathway | 2 | 0.035079615 | GCGR/CRHR2 |
| GO | GO:0004896 | cytokine receptor activity | 2 | 0.035079615 | CD74/FZD4 |
| GO | GO:0014704 | intercalated disc | 2 | 0.035079615 | ITGB1/GJA1 |
| GO | GO:0071456 | cellular response to hypoxia | 3 | 0.035517133 | VEGFA/KCNK2/SIRT4 |
| GO | GO:0034774 | secretory granule lumen | 3 | 0.035517133 | CRISPLD2/S100A9/S100A8 |
| GO | GO:0042981 | regulation of apoptotic process | 4 | 0.035698974 | GJA1/LOX/EGR1/TNFRSF10D |
| GO | GO:0008626 | granzyme-mediated apoptotic signaling pathway | 1 | 0.036215943 | SRGN |
| GO | GO:0010792 | DNA double-strand break processing involved in repair via single-strand annealing | 1 | 0.036215943 | NARF |
| GO | GO:0019960 | C-X3-C chemokine binding | 1 | 0.036215943 | ITGB1 |
| GO | GO:0016071 | mRNA metabolic process | 1 | 0.036215943 | CEBPG |
| GO | GO:0021695 | cerebellar cortex development | 1 | 0.036215943 | EZH2 |
| GO | GO:0031579 | membrane raft organization | 1 | 0.036215943 | NPC1 |
| GO | GO:0071558 | histone demethylase activity (H3-K27 specific) | 1 | 0.036215943 | KDM6B |
| GO | GO:0005353 | fructose transmembrane transporter activity | 1 | 0.036215943 | SLC2A5 |
| GO | GO:0030492 | hemoglobin binding | 1 | 0.036215943 | HBB |
| GO | GO:0051147 | regulation of muscle cell differentiation | 1 | 0.036215943 | IGF2 |
| GO | GO:0030072 | peptide hormone secretion | 1 | 0.036215943 | CPE |
| GO | GO:0014910 | regulation of smooth muscle cell migration | 1 | 0.036215943 | PLXNA1 |
| GO | GO:0071557 | histone H3-K27 demethylation | 1 | 0.036215943 | KDM6B |
| GO | GO:0038033 | positive regulation of endothelial cell chemotaxis by VEGF-activated vascular endothelial growth factor receptor signaling pathway | 1 | 0.036215943 | VEGFA |
| GO | GO:0042159 | lipoprotein catabolic process | 1 | 0.036215943 | APOE |
| GO | GO:0050856 | regulation of T cell receptor signaling pathway | 1 | 0.036215943 | BCL10 |
| GO | GO:0035791 | platelet-derived growth factor receptor-beta signaling pathway | 1 | 0.036215943 | LOX |
| GO | GO:0071504 | cellular response to heparin | 1 | 0.036215943 | EGR1 |
| GO | GO:0035696 | monocyte extravasation | 1 | 0.036215943 | PECAM1 |
| GO | GO:0071332 | cellular response to fructose stimulus | 1 | 0.036215943 | SLC2A5 |
| GO | GO:0042158 | lipoprotein biosynthetic process | 1 | 0.036215943 | APOE |
| GO | GO:2001140 | positive regulation of phospholipid transport | 1 | 0.036215943 | ABCB4 |
| GO | GO:0046923 | ER retention sequence binding | 1 | 0.036215943 | CEMIP |
| GO | GO:0070873 | regulation of glycogen metabolic process | 1 | 0.036215943 | GCGR |
| GO | GO:0004530 | deoxyribonuclease I activity | 1 | 0.036215943 | DNASE1L3 |
| GO | GO:0004758 | serine C-palmitoyltransferase activity | 1 | 0.036215943 | SPTLC2 |
| GO | GO:0098639 | collagen binding involved in cell-matrix adhesion | 1 | 0.036215943 | ITGB1 |
| GO | GO:0070061 | fructose binding | 1 | 0.036215943 | SLC2A5 |
| GO | GO:0046976 | histone methyltransferase activity (H3-K27 specific) | 1 | 0.036215943 | EZH2 |
| GO | GO:0043208 | glycosphingolipid binding | 1 | 0.036215943 | LAMA1 |
| GO | GO:0032376 | positive regulation of cholesterol transport | 1 | 0.036215943 | ABCB4 |
| GO | GO:2000467 | positive regulation of glycogen (starch) synthase activity | 1 | 0.036215943 | IGF2 |
| GO | GO:1902262 | apoptotic process involved in blood vessel morphogenesis | 1 | 0.036215943 | SPI1 |
| GO | GO:0045590 | negative regulation of regulatory T cell differentiation | 1 | 0.036215943 | MDK |
| GO | GO:1902732 | positive regulation of chondrocyte proliferation | 1 | 0.036215943 | LTF |
| GO | GO:1904885 | beta-catenin destruction complex assembly | 1 | 0.036215943 | AMER1 |
| GO | GO:0031726 | CCR1 chemokine receptor binding | 1 | 0.036215943 | CCL3 |
| GO | GO:0043353 | enucleate erythrocyte differentiation | 1 | 0.036215943 | CEBPG |
| GO | GO:0019509 | L-methionine salvage from methylthioadenosine | 1 | 0.036215943 | MRI1 |
| GO | GO:0099699 | integral component of synaptic membrane | 1 | 0.036215943 | ITGB1 |
| GO | GO:0043120 | tumor necrosis factor binding | 1 | 0.036215943 | A2M |
| GO | GO:0045569 | TRAIL binding | 1 | 0.036215943 | TNFRSF10D |
| GO | GO:0030263 | apoptotic chromosome condensation | 1 | 0.036215943 | ERN2 |
| GO | GO:0048386 | positive regulation of retinoic acid receptor signaling pathway | 1 | 0.036215943 | KLF2 |
| GO | GO:0071621 | granulocyte chemotaxis | 1 | 0.036215943 | CCL3 |
| GO | GO:0043932 | ossification involved in bone remodeling | 1 | 0.036215943 | CTHRC1 |
| GO | GO:1905867 | epididymis development | 1 | 0.036215943 | GJA1 |
| GO | GO:0046473 | phosphatidic acid metabolic process | 1 | 0.036215943 | PLA2G2A |
| GO | GO:0032269 | negative regulation of cellular protein metabolic process | 1 | 0.036215943 | APOE |
| GO | GO:0003211 | cardiac ventricle formation | 1 | 0.036215943 | SOX11 |
| GO | GO:0046464 | acylglycerol catabolic process | 1 | 0.036215943 | ABHD12 |
| GO | GO:0017038 | protein import | 1 | 0.036215943 | APOE |
| GO | GO:0004464 | leukotriene-C4 synthase activity | 1 | 0.036215943 | MGST2 |
| GO | GO:0051246 | regulation of protein metabolic process | 1 | 0.036215943 | APOE |
| GO | GO:2001205 | negative regulation of osteoclast development | 1 | 0.036215943 | LTF |
| GO | GO:0036324 | vascular endothelial growth factor receptor-2 signaling pathway | 1 | 0.036215943 | VEGFA |
| GO | GO:0003357 | noradrenergic neuron differentiation | 1 | 0.036215943 | SOX11 |
| GO | GO:1903413 | cellular response to bile acid | 1 | 0.036215943 | ABCB4 |
| GO | GO:0001887 | selenium compound metabolic process | 1 | 0.036215943 | SEPP1 |
| GO | GO:0060509 | type I pneumocyte differentiation | 1 | 0.036215943 | KLF2 |
| GO | GO:2000117 | negative regulation of cysteine-type endopeptidase activity | 1 | 0.036215943 | LTF |
| GO | GO:0017059 | serine C-palmitoyltransferase complex | 1 | 0.036215943 | SPTLC2 |
| GO | GO:1904504 | positive regulation of lipophagy | 1 | 0.036215943 | SPTLC2 |
| GO | GO:0060666 | dichotomous subdivision of terminal units involved in salivary gland branching | 1 | 0.036215943 | PLXNA1 |
| GO | GO:0034363 | intermediate-density lipoprotein particle | 1 | 0.036215943 | APOE |
| GO | GO:0060669 | embryonic placenta morphogenesis | 1 | 0.036215943 | IGF2 |
| GO | GO:0045541 | negative regulation of cholesterol biosynthetic process | 1 | 0.036215943 | APOE |
| GO | GO:0032534 | regulation of microvillus assembly | 1 | 0.036215943 | FSCN1 |
| GO | GO:0060023 | soft palate development | 1 | 0.036215943 | SOX11 |
| GO | GO:0090554 | phosphatidylcholine floppase activity | 1 | 0.036215943 | ABCB4 |
| GO | GO:0001602 | pancreatic polypeptide receptor activity | 1 | 0.036215943 | NPY1R |
| GO | GO:0060351 | cartilage development involved in endochondral bone morphogenesis | 1 | 0.036215943 | COL1A1 |
| GO | GO:0016202 | regulation of striated muscle tissue development | 1 | 0.036215943 | LOX |
| GO | GO:0046850 | regulation of bone remodeling | 1 | 0.036215943 | MDK |
| GO | GO:0006290 | pyrimidine dimer repair | 1 | 0.036215943 | POLH |
| GO | GO:1905907 | negative regulation of amyloid fibril formation | 1 | 0.036215943 | APOE |
| GO | GO:0051525 | NFAT protein binding | 1 | 0.036215943 | SPI1 |
| GO | GO:0036066 | protein O-linked fucosylation | 1 | 0.036215943 | B3GALTL |
| GO | GO:1902991 | regulation of amyloid precursor protein catabolic process | 1 | 0.036215943 | APOE |
| GO | GO:0040016 | embryonic cleavage | 1 | 0.036215943 | TPRA1 |
| GO | GO:0044267 | cellular protein metabolic process | 4 | 0.036251604 | IGF2/APOE/LTF/SPARCL1 |
| GO | GO:0030336 | negative regulation of cell migration | 3 | 0.036283929 | CD74/ARID2/CYP1B1 |
| GO | GO:0051082 | unfolded protein binding | 3 | 0.036283929 | SYVN1/ERN2/AIPL1 |
| GO | GO:0007157 | heterophilic cell-cell adhesion via plasma membrane cell adhesion molecules | 2 | 0.03642757 | DCHS1/HMCN1 |
| GO | GO:0008542 | visual learning | 2 | 0.03642757 | ITGB1/RAG1 |
| GO | GO:0060348 | bone development | 2 | 0.03642757 | GJA1/AMER1 |
| GO | GO:0030178 | negative regulation of Wnt signaling pathway | 2 | 0.03642757 | FRZB/WIF1 |
| GO | GO:0009897 | external side of plasma membrane | 6 | 0.036784334 | ITGB1/CD74/PECAM1/S1PR1/IL13/IGLL5 |
| GO | GO:0043434 | response to peptide hormone | 2 | 0.037795233 | GJA1/COL1A1 |
| GO | GO:0045785 | positive regulation of cell adhesion | 2 | 0.037795233 | MDK/VEGFA |
| GO | GO:0008009 | chemokine activity | 2 | 0.037795233 | CCL18/CCL3 |
| GO | GO:0008284 | positive regulation of cell population proliferation | 7 | 0.038013896 | ITGB1/IGF2/SOX11/EZH2/VEGFA/IL11/FOSL1 |
| GO | GO:0046332 | SMAD binding | 2 | 0.039182315 | COL5A2/COL3A1 |
| GO | GO:0030155 | regulation of cell adhesion | 2 | 0.039182315 | LAMA1/S1PR1 |
| GO | GO:0050890 | cognition | 2 | 0.040588527 | HLA-DRA/ZCCHC16 |
| GO | GO:0032760 | positive regulation of tumor necrosis factor production | 2 | 0.040588527 | SPON2/CCL3 |
| GO | GO:1901215 | negative regulation of neuron death | 2 | 0.040588527 | IL13/TMEM259 |
| GO | GO:0007173 | epidermal growth factor receptor signaling pathway | 2 | 0.040588527 | EFEMP1/SOCS5 |
| GO | GO:0007269 | neurotransmitter secretion | 2 | 0.042013587 | STX1A/SYN1 |
| GO | GO:0050808 | synapse organization | 2 | 0.042013587 | C1QA/SYN1 |
| GO | GO:0010807 | regulation of synaptic vesicle priming | 1 | 0.042123637 | STX1A |
| GO | GO:0031670 | cellular response to nutrient | 1 | 0.042123637 | LPL |
| GO | GO:0033631 | cell-cell adhesion mediated by integrin | 1 | 0.042123637 | ITGB1 |
| GO | GO:0042415 | norepinephrine metabolic process | 1 | 0.042123637 | ZCCHC16 |
| GO | GO:0030853 | negative regulation of granulocyte differentiation | 1 | 0.042123637 | C1QC |
| GO | GO:0035748 | myelin sheath abaxonal region | 1 | 0.042123637 | ITGB1 |
| GO | GO:0106256 | hydroperoxy icosatetraenoate dehydratase activity | 1 | 0.042123637 | CYP1B1 |
| GO | GO:0045646 | regulation of erythrocyte differentiation | 1 | 0.042123637 | SPI1 |
| GO | GO:0009620 | response to fungus | 1 | 0.042123637 | BCL10 |
| GO | GO:0060754 | positive regulation of mast cell chemotaxis | 1 | 0.042123637 | VEGFA |
| GO | GO:2000273 | positive regulation of signaling receptor activity | 1 | 0.042123637 | ITGB1 |
| GO | GO:0061299 | retina vasculature morphogenesis in camera-type eye | 1 | 0.042123637 | FZD4 |
| GO | GO:0007423 | sensory organ development | 1 | 0.042123637 | MAFB |
| GO | GO:0061419 | positive regulation of transcription from RNA polymerase II promoter in response to hypoxia | 1 | 0.042123637 | VEGFA |
| GO | GO:0008354 | germ cell migration | 1 | 0.042123637 | ITGB1 |
| GO | GO:0017083 | 4-galactosyl-N-acetylglucosaminide 3-alpha-L-fucosyltransferase activity | 1 | 0.042123637 | FUT4 |
| GO | GO:0098802 | plasma membrane signaling receptor complex | 1 | 0.042123637 | TRAF1 |
| GO | GO:0032411 | positive regulation of transporter activity | 1 | 0.042123637 | SGK2 |
| GO | GO:0046581 | intercellular canaliculus | 1 | 0.042123637 | ABCB4 |
| GO | GO:0070293 | renal absorption | 1 | 0.042123637 | HBB |
| GO | GO:0061037 | negative regulation of cartilage development | 1 | 0.042123637 | FRZB |
| GO | GO:0098532 | histone H3-K27 trimethylation | 1 | 0.042123637 | EZH2 |
| GO | GO:0001740 | Barr body | 1 | 0.042123637 | H2AFY2 |
| GO | GO:0010644 | cell communication by electrical coupling | 1 | 0.042123637 | GJA1 |
| GO | GO:0032782 | bile acid secretion | 1 | 0.042123637 | ABCB4 |
| GO | GO:0048525 | negative regulation of viral process | 1 | 0.042123637 | LTF |
| GO | GO:0071253 | connexin binding | 1 | 0.042123637 | GJA1 |
| GO | GO:0008483 | transaminase activity | 1 | 0.042123637 | ETNPPL |
| GO | GO:2001236 | regulation of extrinsic apoptotic signaling pathway | 1 | 0.042123637 | TRAF1 |
| GO | GO:0060992 | response to fungicide | 1 | 0.042123637 | KDM6B |
| GO | GO:0072672 | neutrophil extravasation | 1 | 0.042123637 | PECAM1 |
| GO | GO:0003231 | cardiac ventricle development | 1 | 0.042123637 | KCNK2 |
| GO | GO:0070734 | histone H3-K27 methylation | 1 | 0.042123637 | EZH2 |
| GO | GO:0002830 | positive regulation of type 2 immune response | 1 | 0.042123637 | CD74 |
| GO | GO:0005172 | vascular endothelial growth factor receptor binding | 1 | 0.042123637 | VEGFA |
| GO | GO:1904294 | positive regulation of ERAD pathway | 1 | 0.042123637 | TMEM259 |
| GO | GO:0045627 | positive regulation of T-helper 1 cell differentiation | 1 | 0.042123637 | SOCS5 |
| GO | GO:0150051 | postsynaptic Golgi apparatus | 1 | 0.042123637 | TM9SF2 |
| GO | GO:0060022 | hard palate development | 1 | 0.042123637 | SOX11 |
| GO | GO:0043615 | astrocyte cell migration | 1 | 0.042123637 | CCL3 |
| GO | GO:1902949 | positive regulation of tau-protein kinase activity | 1 | 0.042123637 | EGR1 |
| GO | GO:0002232 | leukocyte chemotaxis involved in inflammatory response | 1 | 0.042123637 | MDK |
| GO | GO:0034447 | very-low-density lipoprotein particle clearance | 1 | 0.042123637 | APOE |
| GO | GO:0019065 | receptor-mediated endocytosis of virus by host cell | 1 | 0.042123637 | CFD |
| GO | GO:0042289 | MHC class II protein binding | 1 | 0.042123637 | CD74 |
| GO | GO:0031584 | activation of phospholipase D activity | 1 | 0.042123637 | CACNA1A |
| GO | GO:0036041 | long-chain fatty acid binding | 1 | 0.042123637 | FABP4 |
| GO | GO:0051427 | hormone receptor binding | 1 | 0.042123637 | FABP4 |
| GO | GO:0015755 | fructose transmembrane transport | 1 | 0.042123637 | SLC2A5 |
| GO | GO:0003158 | endothelium development | 1 | 0.042123637 | GJA1 |
| GO | GO:0006308 | DNA catabolic process | 1 | 0.042123637 | DNASE1L3 |
| GO | GO:0044691 | tooth eruption | 1 | 0.042123637 | COL1A1 |
| GO | GO:0001957 | intramembranous ossification | 1 | 0.042123637 | COL1A1 |
| GO | GO:0070231 | T cell apoptotic process | 1 | 0.042123637 | BCL10 |
| GO | GO:1902065 | response to L-glutamate | 1 | 0.042123637 | CACNA1A |
| GO | GO:0010385 | double-stranded methylated DNA binding | 1 | 0.042123637 | EGR1 |
| GO | GO:0060228 | phosphatidylcholine-sterol O-acyltransferase activator activity | 1 | 0.042123637 | APOE |
| GO | GO:0046322 | negative regulation of fatty acid oxidation | 1 | 0.042123637 | SIRT4 |
| GO | GO:0001956 | positive regulation of neurotransmitter secretion | 1 | 0.042123637 | STX1A |
| GO | GO:0023052 | signaling | 1 | 0.042123637 | CCL3 |
| GO | GO:0004528 | phosphodiesterase I activity | 1 | 0.042123637 | FSCN1 |
| GO | GO:2001268 | negative regulation of cysteine-type endopeptidase activity involved in apoptotic signaling pathway | 1 | 0.042123637 | MMP9 |
| GO | GO:0051354 | negative regulation of oxidoreductase activity | 1 | 0.042123637 | GZMA |
| GO | GO:0002283 | neutrophil activation involved in immune response | 1 | 0.042123637 | DNASE1L3 |
| GO | GO:1901727 | positive regulation of histone deacetylase activity | 1 | 0.042123637 | VEGFA |
| GO | GO:0055077 | gap junction hemi-channel activity | 1 | 0.042123637 | GJA1 |
| GO | GO:2000503 | positive regulation of natural killer cell chemotaxis | 1 | 0.042123637 | CCL3 |
| GO | GO:0097443 | sorting endosome | 1 | 0.042123637 | ARHGAP1 |
| GO | GO:1900020 | positive regulation of protein kinase C activity | 1 | 0.042123637 | CEMIP |
| GO | GO:0046085 | adenosine metabolic process | 1 | 0.042123637 | NT5C1B |
| GO | GO:0006686 | sphingomyelin biosynthetic process | 1 | 0.042123637 | SPTLC2 |
| GO | GO:0010994 | free ubiquitin chain polymerization | 1 | 0.042123637 | UBE2C |
| GO | GO:0045347 | negative regulation of MHC class II biosynthetic process | 1 | 0.042123637 | SPI1 |
| GO | GO:0071638 | negative regulation of monocyte chemotactic protein-1 production | 1 | 0.042123637 | SOCS5 |
| GO | GO:1901164 | negative regulation of trophoblast cell migration | 1 | 0.042123637 | GJA1 |
| GO | GO:0031960 | response to corticosteroid | 1 | 0.042123637 | COL1A1 |
| GO | GO:0042742 | defense response to bacterium | 4 | 0.0426564 | S100A9/IRF8/IGLL5/S100A8 |
| GO | GO:1904813 | ficolin-1-rich granule lumen | 3 | 0.042722016 | CRISPLD2/HBB/MMP9 |
| GO | GO:0010629 | negative regulation of gene expression | 4 | 0.043268297 | SOX11/APOE/VEGFA/CCL3 |
| GO | GO:0000287 | magnesium ion binding | 4 | 0.04576526 | WEE1/MTHFD2/FSCN1/ERN2 |
| GO | GO:0042995 | cell projection | 3 | 0.046141158 | CACNA1A/MRI1/MDK |
| GO | GO:0005506 | iron ion binding | 3 | 0.046141158 | PLOD1/CYP1B1/LTF |
| GO | GO:0070936 | protein K48-linked ubiquitination | 2 | 0.046399041 | SYVN1/UBE2C |
| GO | GO:0032154 | cleavage furrow | 2 | 0.046399041 | ITGB1/HMCN1 |
| GO | GO:0032757 | positive regulation of interleukin-8 production | 2 | 0.046399041 | CD74/BCL10 |
| GO | GO:0030170 | pyridoxal phosphate binding | 2 | 0.046399041 | SPTLC2/ETNPPL |
| GO | GO:0002931 | response to ischemia | 2 | 0.046399041 | GJA1/EGR1 |
| GO | GO:0004672 | protein kinase activity | 4 | 0.04704334 | CCL3/WEE1/ERN2/ULK3 |
| GO | GO:0006006 | glucose metabolic process | 2 | 0.047896696 | IGF2/NPY1R |
| GO | GO:1903078 | positive regulation of protein localization to plasma membrane | 2 | 0.047896696 | ITGB1/CLIP3 |
| GO | GO:0001650 | fibrillar center | 3 | 0.047900041 | TAF4B/FAP/MRI1 |
| GO | GO:0061384 | heart trabecula morphogenesis | 1 | 0.047995267 | S1PR1 |
| GO | GO:0046653 | tetrahydrofolate metabolic process | 1 | 0.047995267 | MTHFD2 |
| GO | GO:0061676 | importin-alpha family protein binding | 1 | 0.047995267 | CDADC1 |
| GO | GO:0042985 | negative regulation of amyloid precursor protein biosynthetic process | 1 | 0.047995267 | ITM2A |
| GO | GO:0033157 | regulation of intracellular protein transport | 1 | 0.047995267 | LPL |
| GO | GO:0030157 | pancreatic juice secretion | 1 | 0.047995267 | FAP |
| GO | GO:1900223 | positive regulation of amyloid-beta clearance | 1 | 0.047995267 | APOE |
| GO | GO:1900122 | positive regulation of receptor binding | 1 | 0.047995267 | MMP9 |
| GO | GO:0070244 | negative regulation of thymocyte apoptotic process | 1 | 0.047995267 | RAG1 |
| GO | GO:0031987 | locomotion involved in locomotory behavior | 1 | 0.047995267 | FZD4 |
| GO | GO:0048842 | positive regulation of axon extension involved in axon guidance | 1 | 0.047995267 | VEGFA |
| GO | GO:0002639 | positive regulation of immunoglobulin production | 1 | 0.047995267 | IL13 |
| GO | GO:0009404 | toxin metabolic process | 1 | 0.047995267 | CYP1B1 |
| GO | GO:0046887 | positive regulation of hormone secretion | 1 | 0.047995267 | SOX11 |
| GO | GO:0002606 | positive regulation of dendritic cell antigen processing and presentation | 1 | 0.047995267 | CD74 |
| GO | GO:0004536 | deoxyribonuclease activity | 1 | 0.047995267 | DNASE1L3 |
| GO | GO:0031730 | CCR5 chemokine receptor binding | 1 | 0.047995267 | CCL3 |
| GO | GO:0001841 | neural tube formation | 1 | 0.047995267 | SOX11 |
| GO | GO:0001517 | N-acetylglucosamine 6-O-sulfotransferase activity | 1 | 0.047995267 | CHST1 |
| GO | GO:0052651 | monoacylglycerol catabolic process | 1 | 0.047995267 | ABHD12 |
| GO | GO:0017185 | peptidyl-lysine hydroxylation | 1 | 0.047995267 | PLOD1 |
| GO | GO:0044241 | lipid digestion | 1 | 0.047995267 | FAP |
| GO | GO:0006596 | polyamine biosynthetic process | 1 | 0.047995267 | SMOX |
| GO | GO:0014033 | neural crest cell differentiation | 1 | 0.047995267 | FRZB |
| GO | GO:2000987 | positive regulation of behavioral fear response | 1 | 0.047995267 | GJA1 |
| GO | GO:0032489 | regulation of Cdc42 protein signal transduction | 1 | 0.047995267 | APOE |
| GO | GO:0036003 | positive regulation of transcription from RNA polymerase II promoter in response to stress | 1 | 0.047995267 | KLF2 |
| GO | GO:0061469 | regulation of type B pancreatic cell proliferation | 1 | 0.047995267 | NR4A1 |
| GO | GO:0019209 | kinase activator activity | 1 | 0.047995267 | BCL10 |
| GO | GO:0016308 | 1-phosphatidylinositol-4-phosphate 5-kinase activity | 1 | 0.047995267 | CFD |
| GO | GO:1901165 | positive regulation of trophoblast cell migration | 1 | 0.047995267 | VEGFA |
| GO | GO:0031394 | positive regulation of prostaglandin biosynthetic process | 1 | 0.047995267 | CD74 |
| GO | GO:0010886 | positive regulation of cholesterol storage | 1 | 0.047995267 | LPL |
| GO | GO:0045581 | negative regulation of T cell differentiation | 1 | 0.047995267 | CD74 |
| GO | GO:0045120 | pronucleus | 1 | 0.047995267 | EZH2 |
| GO | GO:2000144 | positive regulation of DNA-templated transcription, initiation | 1 | 0.047995267 | FOSL1 |
| GO | GO:0016004 | phospholipase activator activity | 1 | 0.047995267 | CCL3 |
| GO | GO:0004415 | hyalurononglucosaminidase activity | 1 | 0.047995267 | CEMIP |
| GO | GO:1904141 | positive regulation of microglial cell migration | 1 | 0.047995267 | CCL3 |
| GO | GO:0019966 | interleukin-1 binding | 1 | 0.047995267 | A2M |
| GO | GO:2000766 | negative regulation of cytoplasmic translation | 1 | 0.047995267 | UNK |
| GO | GO:0006004 | fucose metabolic process | 1 | 0.047995267 | B3GALTL |
| GO | GO:1905538 | polysome binding | 1 | 0.047995267 | UNK |
| GO | GO:0140036 | ubiquitin-dependent protein binding | 1 | 0.047995267 | FSCN1 |
| GO | GO:0043152 | induction of bacterial agglutination | 1 | 0.047995267 | SPON2 |
| GO | GO:0071494 | cellular response to UV-C | 1 | 0.047995267 | POLH |
| GO | GO:0010042 | response to manganese ion | 1 | 0.047995267 | FIBIN |
| GO | GO:0045650 | negative regulation of macrophage differentiation | 1 | 0.047995267 | C1QC |
| GO | GO:0002320 | lymphoid progenitor cell differentiation | 1 | 0.047995267 | SPI1 |
| GO | GO:0051101 | regulation of DNA binding | 1 | 0.047995267 | HJURP |
| GO | GO:0010828 | positive regulation of glucose transmembrane transport | 1 | 0.047995267 | CLIP3 |
| GO | GO:0000480 | endonucleolytic cleavage in 5'-ETS of tricistronic rRNA transcript (SSU-rRNA, 5.8S rRNA, LSU-rRNA) | 1 | 0.047995267 | FCF1 |
| GO | GO:0007379 | segment specification | 1 | 0.047995267 | MAFB |
| GO | GO:1901385 | regulation of voltage-gated calcium channel activity | 1 | 0.047995267 | CACNA1A |
| GO | GO:0034979 | NAD-dependent protein deacetylase activity | 1 | 0.047995267 | SIRT4 |
| GO | GO:0030213 | hyaluronan biosynthetic process | 1 | 0.047995267 | CEMIP |
| GO | GO:0071499 | cellular response to laminar fluid shear stress | 1 | 0.047995267 | KLF2 |
| GO | GO:0051593 | response to folic acid | 1 | 0.047995267 | TYMS |
| GO | GO:0030098 | lymphocyte differentiation | 1 | 0.047995267 | SPI1 |
| GO | GO:2000391 | positive regulation of neutrophil extravasation | 1 | 0.047995267 | MDK |
| GO | GO:0010649 | regulation of cell communication by electrical coupling | 1 | 0.047995267 | GJA1 |
| GO | GO:0045202 | synapse | 6 | 0.048336196 | CACNA1A/FAP/C1QC/C1QB/C1QA/NMNAT2 |
| GO | GO:0016485 | protein processing | 2 | 0.049411817 | SRGN/CPE |
| GO | GO:0071560 | cellular response to transforming growth factor beta stimulus | 2 | 0.049411817 | POSTN/COL1A1 |
| GO | GO:0007266 | Rho protein signal transduction | 2 | 0.049411817 | PECAM1/ARHGAP1 |
| GO | GO:0030665 | clathrin-coated vesicle membrane | 2 | 0.049411817 | CEMIP/FZD4 |
| GO | GO:0046854 | phosphatidylinositol phosphorylation | 2 | 0.049411817 | SOCS5/CFD |
| GO | GO:0005765 | lysosomal membrane | 5 | 0.049550623 | CD74/WDR41/HLA-DRA/NPC1/HLA-DRB5 |
| GO | GO:0010008 | endosome membrane | 4 | 0.049658595 | CFD/ARHGAP1/PARM1/TM9SF2 |
| GO | GO:0017124 | SH3 domain binding | 3 | 0.049691502 | GJA1/ADAM12/ARHGAP1 |

**Abbreviation**: GO, gene ontology; KEGG, Kyoto Encyclopedia of Genes and Genomes; DEGs, differentially expressed genes;
