# Supplementary material for: LTF induces senescence and degeneration in the meniscus via the NF-κB signaling pathway: A study based on integrated bioinformatics analysis and experimental validation
Source: Front Mol Biosci. 2023 Apr 24;10:1134253. doi: 10.3389/fmolb.2023.1134253 (PMC10164984; doi:10.3389/fmolb.2023.1134253)
Supplement: Supplementary file 4 [file Table5.DOCX]

**Table S5. Details of** **36 node genes and identification of hub gene in the PPI network.**

| **node genes** | **MCC** | **MNC** | **Degree** |
| --- | --- | --- | --- |
| PECAM1 | 417 | 18 | 19 |
| APOE | 328 | 16 | 18 |
| S1PR1 | 266 | 11 | 11 |
| CCL3 | 240 | 12 | 12 |
| SPARCL1 | 158 | 9 | 9 |
| ACKR1 | 152 | 8 | 8 |
| LTF | 133 | 11 | 12 |
| RGS5 | 121 | 5 | 6 |
| KLF2 | 108 | 9 | 9 |
| GZMA | 100 | 9 | 9 |
| CAMP | 85 | 9 | 10 |
| GPR34 | 50 | 6 | 6 |
| CFD | 48 | 5 | 5 |
| NR2F2 | 48 | 7 | 7 |
| GIMAP1 | 28 | 6 | 6 |
| DEFA4 | 24 | 4 | 4 |
| DEFA3 | 24 | 4 | 4 |
| PLA2G2A | 19 | 5 | 6 |
| IGF2 | 14 | 5 | 5 |
| HEY1 | 12 | 4 | 4 |
| TMEM176B | 7 | 4 | 5 |
| TFPI | 6 | 3 | 3 |
| CALCRL | 4 | 2 | 4 |
| TMEM176A | 3 | 2 | 3 |
| CTHRC1 | 3 | 1 | 3 |
| ITM2A | 3 | 1 | 3 |
| TSPAN7 | 3 | 2 | 3 |
| NPY1R | 2 | 1 | 2 |
| CYP1B1 | 2 | 2 | 2 |
| CACNA1A | 2 | 1 | 2 |
| ERN2 | 1 | 1 | 1 |
| CSN1S1 | 1 | 1 | 1 |
| CAPN6 | 1 | 1 | 1 |
| DNASE1L3 | 1 | 1 | 1 |
| CA12 | 1 | 1 | 1 |
| ACSS3 | 1 | 1 | 1 |
