# Supplementary material for: LTF induces senescence and degeneration in the meniscus via the NF-κB signaling pathway: A study based on integrated bioinformatics analysis and experimental validation
Source: Front Mol Biosci. 2023 Apr 24;10:1134253. doi: 10.3389/fmolb.2023.1134253 (PMC10164984; doi:10.3389/fmolb.2023.1134253)
Supplement: Supplementary file 5 [file Table3.DOCX]

**Table S3. Details of the co-DEGs.**

| **co-DEGs** | **logFC** | ***P* Value** |
| --- | --- | --- |
| XLOC_l2_004647 | -3.23942 | 2.53E-06 |
| GIMAP1 | 1.76259 | 5.96E-05 |
| ACKR1 | 1.80522 | 0.000768 |
| CSN1S1 | 2.859327 | 6.68E-10 |
| CA12 | -1.61935 | 0.000164 |
| SNORD45A | 1.731173 | 0.000214 |
| XLOC_l2_006926 | -1.3979 | 0.000206 |
| GZMA | 1.550892 | 3.65E-07 |
| PARM1 | 1.107538 | 4.04E-05 |
| RGS5 | 1.771192 | 1.06E-08 |
| CAPN6 | 1.237809 | 0.011554 |
| SPARCL1 | 2.188305 | 9.09E-07 |
| TSPAN7 | 1.824609 | 1.32E-07 |
| DNASE1L3 | 2.352694 | 0.000572 |
| TFPI | 1.48442 | 1.63E-07 |
| CRIPAK | -1.34072 | 3.06E-06 |
| LOC101927237 | 1.505902 | 9.47E-08 |
| NR2F2 | 1.364011 | 5.18E-06 |
| PECAM1 | 1.369668 | 0.000106 |
| ACSS3 | 1.31931 | 8.95E-05 |
| SNORA12 | 1.633044 | 2.18E-06 |
| IGF2 | 1.078659 | 0.007116 |
| SNORA23 | 1.795827 | 2.76E-05 |
| AXDND1 | -1.82196 | 0.000353 |
| PLA2G2A | 1.87872 | 5.21E-07 |
| ERN2 | -1.36754 | 1.19E-05 |
| CFD | 2.111333 | 2.85E-09 |
| CALCRL | 1.305427 | 5.6E-08 |
| CAMP | 1.276371 | 0.007431 |
| DEFA3 | 2.007798 | 8.51E-07 |
| DEFA4 | 1.474462 | 1.87E-06 |
| TMEM176A | 1.422695 | 1.01E-07 |
| SNORA18 | 1.002052 | 0.000505 |
| KIAA1522 | -1.03342 | 1.96E-07 |
| RAD54L | -1.0538 | 0.000119 |
| CCL3 | 1.267096 | 3.49E-05 |
| CYP1B1 | 1.369035 | 1.97E-06 |
| CTHRC1 | -1.02969 | 1.75E-05 |
| ITM2A | 1.078536 | 0.000196 |
| SNORD32A | 1.024458 | 0.000282 |
| HEY1 | 1.182363 | 6.86E-06 |
| APOE | 1.452113 | 3.39E-05 |
| GPR34 | 1.360351 | 3.96E-05 |
| S1PR1 | 1.019862 | 8.54E-07 |
| XLOC_l2_015590 | 1.094845 | 2.96E-05 |
| KLF2 | 1.01068 | 7.68E-05 |
| SNORD33 | 1.038544 | 0.001083 |
| NPY1R | 1.14157 | 0.000281 |
| LTF | 1.292296 | 1.78E-06 |
| TMEM176B | 1.087537 | 1.5E-06 |
| CACNA1A | -1.15157 | 1.51E-06 |
| LOC102724332 | 2.147086 | 0.000022 |

**Abbreviation**: Co-DEGs, common differentially expressed genes.
