# Supplementary material for: LTF induces senescence and degeneration in the meniscus via the NF-κB signaling pathway: A study based on integrated bioinformatics analysis and experimental validation
Source: Front Mol Biosci. 2023 Apr 24;10:1134253. doi: 10.3389/fmolb.2023.1134253 (PMC10164984; doi:10.3389/fmolb.2023.1134253)
Supplement: Supplementary file 6 [file Table4.DOCX]

**Table S4. Detailed results of the KEGG and GO enrichment analysis of the co-DEGs.**

| **Group** | **ID** | **Description** | **Count** | ***P* Value** | **Genes** |
| --- | --- | --- | --- | --- | --- |
| KEGG | hsa04080 | Neuroactive ligand-receptor interaction | 4 | 0.0006899 | S1PR1/NPY1R/GZMA/CALCRL |
| KEGG | hsa05144 | Malaria | 2 | 0.0016484 | PECAM1/ACKR1 |
| KEGG | hsa05150 | Staphylococcus aureus infection | 2 | 0.0030785 | DEFA4/DEFA3 |
| KEGG | hsa04270 | Vascular smooth muscle contraction | 2 | 0.0108327 | PLA2G2A/CALCRL |
| KEGG | hsa04068 | FoxO signaling pathway | 2 | 0.0108327 | S1PR1/KLF2 |
| KEGG | hsa05418 | Fluid shear stress and atherosclerosis | 2 | 0.0119366 | PECAM1/KLF2 |
| KEGG | hsa04621 | NOD-like receptor signaling pathway | 2 | 0.0189304 | DEFA4/DEFA3 |
| KEGG | hsa05202 | Transcriptional misregulation in cancer | 2 | 0.0205341 | DEFA3/TSPAN7 |
| KEGG | hsa00910 | Nitrogen metabolism | 1 | 0.0208701 | CA12 |
| KEGG | hsa00592 | alpha-Linolenic acid metabolism | 1 | 0.0300084 | PLA2G2A |
| KEGG | hsa04014 | Ras signaling pathway | 2 | 0.0307868 | PLA2G2A/IGF2 |
| KEGG | hsa00591 | Linoleic acid metabolism | 1 | 0.0345462 | PLA2G2A |
| KEGG | hsa00640 | Propanoate metabolism | 1 | 0.0401892 | ACSS3 |
| KEGG | hsa05033 | Nicotine addiction | 1 | 0.0469183 | CACNA1A |
| KEGG | hsa04010 | MAPK signaling pathway | 2 | 0.0474046 | IGF2/CACNA1A |
| KEGG | hsa03440 | Homologous recombination | 1 | 0.0480353 | RAD54L |
| KEGG | hsa04975 | Fat digestion and absorption | 1 | 0.0480353 | PLA2G2A |
| KEGG | hsa00380 | Tryptophan metabolism | 1 | 0.049151 | CYP1B1 |
| GO | GO:0005576 | extracellular region | 13 | 1.32E-07 | IGF2/CCL3/DNASE1L3/GZMA/CSN1S1/SPARCL1/PLA2G2A/DEFA4/DEFA3/LTF/TFPI/CTHRC1/APOE |
| GO | GO:0005615 | extracellular space | 12 | 1.93E-07 | IGF2/CCL3/APOE/PECAM1/CSN1S1/SPARCL1/PLA2G2A/DEFA4/DEFA3/LTF/TFPI/CTHRC1 |
| GO | GO:0051673 | membrane disruption in other organism | 3 | 2.46E-07 | DEFA4/DEFA3/LTF |
| GO | GO:0019730 | antimicrobial humoral response | 4 | 6.66E-07 | PLA2G2A/DEFA4/DEFA3/LTF |
| GO | GO:0002227 | innate immune response in mucosa | 3 | 2.62E-06 | DEFA4/DEFA3/LTF |
| GO | GO:0001525 | angiogenesis | 5 | 9.68E-06 | PECAM1/S1PR1/CALCRL/CYP1B1/HEY1 |
| GO | GO:0031640 | killing of cells of other organism | 3 | 1.23E-05 | DEFA4/DEFA3/LTF |
| GO | GO:0019731 | antibacterial humoral response | 3 | 1.92E-05 | DEFA4/DEFA3/LTF |
| GO | GO:0019732 | antifungal humoral response | 2 | 2.81E-05 | DEFA4/LTF |
| GO | GO:2001199 | negative regulation of dendritic cell differentiation | 2 | 3.74E-05 | TMEM176A/TMEM176B |
| GO | GO:0007186 | G protein-coupled receptor signaling pathway | 8 | 4.80E-05 | CCL3/APOE/CALCRL/S1PR1/NPY1R/ACKR1/RGS5/GPR34 |
| GO | GO:0061844 | antimicrobial humoral immune response mediated by antimicrobial peptide | 3 | 6.23E-05 | DEFA4/DEFA3/LTF |
| GO | GO:0044267 | cellular protein metabolic process | 4 | 9.35E-05 | IGF2/APOE/LTF/SPARCL1 |
| GO | GO:0006816 | calcium ion transport | 3 | 0.0001074 | CCL3/CACNA1A/CALCRL |
| GO | GO:0033690 | positive regulation of osteoblast proliferation | 2 | 0.0001209 | LTF/CTHRC1 |
| GO | GO:0042803 | protein homodimerization activity | 6 | 0.0001221 | PECAM1/APOE/GZMA/NR2F2/DEFA4/DEFA3 |
| GO | GO:0001540 | amyloid-beta binding | 3 | 0.0001292 | CACNA1A/ITM2A/APOE |
| GO | GO:0071347 | cellular response to interleukin-1 | 3 | 0.0001292 | CCL3/KLF2/TFPI |
| GO | GO:0050829 | defense response to Gram-negative bacterium | 3 | 0.0001339 | DEFA4/DEFA3/LTF |
| GO | GO:0030195 | negative regulation of blood coagulation | 2 | 0.0001394 | APOE/TFPI |
| GO | GO:0005515 | protein binding | 26 | 0.0001671 | CCL3/SPARCL1/PARM1/PECAM1/APOE/RGS5/CACNA1A/S1PR1/CAMP/NR2F2/CAPN6/KLF2/IGF2/CALCRL/DNASE1L3/TMEM176A/TSPAN7/TMEM176B/GIMAP1/HEY1/GZMA/CFD/NPY1R/ITM2A/CSN1S1/LTF |
| GO | GO:0034374 | low-density lipoprotein particle remodeling | 2 | 0.0002027 | PLA2G2A/APOE |
| GO | GO:0050830 | defense response to Gram-positive bacterium | 3 | 0.0002243 | PLA2G2A/DEFA4/DEFA3 |
| GO | GO:0019835 | cytolysis | 2 | 0.0002514 | PLA2G2A/GZMA |
| GO | GO:0032355 | response to estradiol | 3 | 0.0002813 | CSN1S1/TFPI/NR2F2 |
| GO | GO:0048514 | blood vessel morphogenesis | 2 | 0.0003052 | CYP1B1/NR2F2 |
| GO | GO:0010596 | negative regulation of endothelial cell migration | 2 | 0.0003641 | APOE/NR2F2 |
| GO | GO:0050832 | defense response to fungus | 2 | 0.0005713 | DEFA4/DEFA3 |
| GO | GO:0043539 | protein serine/threonine kinase activator activity | 2 | 0.0006504 | IGF2/LTF |
| GO | GO:0001937 | negative regulation of endothelial cell proliferation | 2 | 0.0006918 | APOE/NR2F2 |
| GO | GO:0043542 | endothelial cell migration | 2 | 0.0006918 | PECAM1/CYP1B1 |
| GO | GO:0071222 | cellular response to lipopolysaccharide | 3 | 0.0009008 | DEFA4/DEFA3/TFPI |
| GO | GO:0005783 | endoplasmic reticulum | 6 | 0.0011884 | APOE/CALCRL/DNASE1L3/PLA2G2A/TFPI/GIMAP1 |
| GO | GO:0071407 | cellular response to organic cyclic compound | 2 | 0.0014017 | CCL3/CYP1B1 |
| GO | GO:0048856 | anatomical structure development | 2 | 0.0014017 | SPARCL1/NR2F2 |
| GO | GO:0071902 | positive regulation of protein serine/threonine kinase activity | 2 | 0.0017131 | IGF2/LTF |
| GO | GO:0070374 | positive regulation of ERK1 and ERK2 cascade | 3 | 0.001929 | PLA2G2A/CCL3/APOE |
| GO | GO:0005886 | plasma membrane | 13 | 0.0019641 | PECAM1/APOE/CALCRL/CACNA1A/S1PR1/NPY1R/ACKR1/CA12/PLA2G2A/RGS5/ITM2A/TFPI/PARM1 |
| GO | GO:0006006 | glucose metabolic process | 2 | 0.0020542 | IGF2/NPY1R |
| GO | GO:0048661 | positive regulation of smooth muscle cell proliferation | 2 | 0.0022731 | S1PR1/CALCRL |
| GO | GO:0045669 | positive regulation of osteoblast differentiation | 2 | 0.0024249 | LTF/CTHRC1 |
| GO | GO:0007193 | adenylate cyclase-inhibiting G protein-coupled receptor signaling pathway | 2 | 0.0024249 | S1PR1/NPY1R |
| GO | GO:0030335 | positive regulation of cell migration | 3 | 0.0024964 | CCL3/S1PR1/PECAM1 |
| GO | GO:0035580 | specific granule lumen | 2 | 0.0025813 | DEFA4/LTF |
| GO | GO:0045121 | membrane raft | 3 | 0.002815 | PECAM1/CFD/S1PR1 |
| GO | GO:0007187 | G protein-coupled receptor signaling pathway, coupled to cyclic nucleotide second messenger | 2 | 0.0028247 | NPY1R/CALCRL |
| GO | GO:0070301 | cellular response to hydrogen peroxide | 2 | 0.0029927 | CYP1B1/KLF2 |
| GO | GO:0070098 | chemokine-mediated signaling pathway | 2 | 0.0029927 | CCL3/ACKR1 |
| GO | GO:0005769 | early endosome | 3 | 0.0039168 | APOE/ACKR1/PARM1 |
| GO | GO:0045893 | positive regulation of transcription, DNA-templated | 4 | 0.0041499 | NR2F2/KLF2/APOE/HEY1 |
| GO | GO:0050729 | positive regulation of inflammatory response | 2 | 0.0042962 | PLA2G2A/CCL3 |
| GO | GO:0019722 | calcium-mediated signaling | 2 | 0.0043977 | CCL3/CACNA1A |
| GO | GO:0009636 | response to toxic substance | 2 | 0.0043977 | CCL3/CYP1B1 |
| GO | GO:0050731 | positive regulation of peptidyl-tyrosine phosphorylation | 2 | 0.0048148 | IGF2/PECAM1 |
| GO | GO:0030141 | secretory granule | 2 | 0.0054734 | PLA2G2A/LTF |
| GO | GO:0043547 | positive regulation of GTPase activity | 3 | 0.005747 | CCL3/S1PR1/RGS5 |
| GO | GO:0050728 | negative regulation of inflammatory response | 2 | 0.0058173 | APOE/CALCRL |
| GO | GO:0009952 | anterior/posterior pattern specification | 2 | 0.0059341 | NR2F2/HEY1 |
| GO | GO:0006874 | cellular calcium ion homeostasis | 2 | 0.0061709 | CCL3/APOE |
| GO | GO:0005796 | Golgi lumen | 2 | 0.0065341 | DEFA4/DEFA3 |
| GO | GO:0060669 | embryonic placenta morphogenesis | 1 | 0.0070046 | IGF2 |
| GO | GO:0030263 | apoptotic chromosome condensation | 1 | 0.0070046 | ERN2 |
| GO | GO:0048386 | positive regulation of retinoic acid receptor signaling pathway | 1 | 0.0070046 | KLF2 |
| GO | GO:0071621 | granulocyte chemotaxis | 1 | 0.0070046 | CCL3 |
| GO | GO:0043932 | ossification involved in bone remodeling | 1 | 0.0070046 | CTHRC1 |
| GO | GO:0034363 | intermediate-density lipoprotein particle | 1 | 0.0070046 | APOE |
| GO | GO:0004530 | deoxyribonuclease I activity | 1 | 0.0070046 | DNASE1L3 |
| GO | GO:0046473 | phosphatidic acid metabolic process | 1 | 0.0070046 | PLA2G2A |
| GO | GO:0032269 | negative regulation of cellular protein metabolic process | 1 | 0.0070046 | APOE |
| GO | GO:0045541 | negative regulation of cholesterol biosynthetic process | 1 | 0.0070046 | APOE |
| GO | GO:0017038 | protein import | 1 | 0.0070046 | APOE |
| GO | GO:0051246 | regulation of protein metabolic process | 1 | 0.0070046 | APOE |
| GO | GO:0042159 | lipoprotein catabolic process | 1 | 0.0070046 | APOE |
| GO | GO:0001602 | pancreatic polypeptide receptor activity | 1 | 0.0070046 | NPY1R |
| GO | GO:0042158 | lipoprotein biosynthetic process | 1 | 0.0070046 | APOE |
| GO | GO:2001205 | negative regulation of osteoclast development | 1 | 0.0070046 | LTF |
| GO | GO:2000467 | positive regulation of glycogen (starch) synthase activity | 1 | 0.0070046 | IGF2 |
| GO | GO:0051147 | regulation of muscle cell differentiation | 1 | 0.0070046 | IGF2 |
| GO | GO:1905907 | negative regulation of amyloid fibril formation | 1 | 0.0070046 | APOE |
| GO | GO:1902991 | regulation of amyloid precursor protein catabolic process | 1 | 0.0070046 | APOE |
| GO | GO:1902732 | positive regulation of chondrocyte proliferation | 1 | 0.0070046 | LTF |
| GO | GO:0060509 | type I pneumocyte differentiation | 1 | 0.0070046 | KLF2 |
| GO | GO:2000117 | negative regulation of cysteine-type endopeptidase activity | 1 | 0.0070046 | LTF |
| GO | GO:0031726 | CCR1 chemokine receptor binding | 1 | 0.0070046 | CCL3 |
| GO | GO:0035696 | monocyte extravasation | 1 | 0.0070046 | PECAM1 |
| GO | GO:0001649 | osteoblast differentiation | 2 | 0.0071606 | IGF2/CCL3 |
| GO | GO:0005543 | phospholipid binding | 2 | 0.007549 | PLA2G2A/APOE |
| GO | GO:0007189 | adenylate cyclase-activating G protein-coupled receptor signaling pathway | 2 | 0.0080815 | S1PR1/CALCRL |
| GO | GO:0060228 | phosphatidylcholine-sterol O-acyltransferase activator activity | 1 | 0.0081674 | APOE |
| GO | GO:0106256 | hydroperoxy icosatetraenoate dehydratase activity | 1 | 0.0081674 | CYP1B1 |
| GO | GO:0051354 | negative regulation of oxidoreductase activity | 1 | 0.0081674 | GZMA |
| GO | GO:0048525 | negative regulation of viral process | 1 | 0.0081674 | LTF |
| GO | GO:0072672 | neutrophil extravasation | 1 | 0.0081674 | PECAM1 |
| GO | GO:0006308 | DNA catabolic process | 1 | 0.0081674 | DNASE1L3 |
| GO | GO:0002283 | neutrophil activation involved in immune response | 1 | 0.0081674 | DNASE1L3 |
| GO | GO:2000503 | positive regulation of natural killer cell chemotaxis | 1 | 0.0081674 | CCL3 |
| GO | GO:0061304 | retinal blood vessel morphogenesis | 1 | 0.0081674 | CYP1B1 |
| GO | GO:1902065 | response to L-glutamate | 1 | 0.0081674 | CACNA1A |
| GO | GO:0043615 | astrocyte cell migration | 1 | 0.0081674 | CCL3 |
| GO | GO:0023052 | signaling | 1 | 0.0081674 | CCL3 |
| GO | GO:0034447 | very-low-density lipoprotein particle clearance | 1 | 0.0081674 | APOE |
| GO | GO:0019065 | receptor-mediated endocytosis of virus by host cell | 1 | 0.0081674 | CFD |
| GO | GO:0031584 | activation of phospholipase D activity | 1 | 0.0081674 | CACNA1A |
| GO | GO:0098978 | glutamatergic synapse | 3 | 0.0084597 | CACNA1A/APOE/SPARCL1 |
| GO | GO:0016021 | integral component of membrane | 10 | 0.0085877 | CACNA1A/S1PR1/ACKR1/ERN2/ITM2A/TMEM176A/TMEM176B/CA12/GIMAP1/PARM1 |
| GO | GO:0005509 | calcium ion binding | 4 | 0.0088875 | PLA2G2A/CACNA1A/DNASE1L3/SPARCL1 |
| GO | GO:0006935 | chemotaxis | 2 | 0.008911 | CCL3/S1PR1 |
| GO | GO:0061384 | heart trabecula morphogenesis | 1 | 0.0093288 | S1PR1 |
| GO | GO:0004536 | deoxyribonuclease activity | 1 | 0.0093288 | DNASE1L3 |
| GO | GO:0016308 | 1-phosphatidylinositol-4-phosphate 5-kinase activity | 1 | 0.0093288 | CFD |
| GO | GO:1904141 | positive regulation of microglial cell migration | 1 | 0.0093288 | CCL3 |
| GO | GO:0031730 | CCR5 chemokine receptor binding | 1 | 0.0093288 | CCL3 |
| GO | GO:0016004 | phospholipase activator activity | 1 | 0.0093288 | CCL3 |
| GO | GO:1900223 | positive regulation of amyloid-beta clearance | 1 | 0.0093288 | APOE |
| GO | GO:1901385 | regulation of voltage-gated calcium channel activity | 1 | 0.0093288 | CACNA1A |
| GO | GO:0071813 | lipoprotein particle binding | 1 | 0.0093288 | APOE |
| GO | GO:0032489 | regulation of Cdc42 protein signal transduction | 1 | 0.0093288 | APOE |
| GO | GO:0009404 | toxin metabolic process | 1 | 0.0093288 | CYP1B1 |
| GO | GO:0042985 | negative regulation of amyloid precursor protein biosynthetic process | 1 | 0.0093288 | ITM2A |
| GO | GO:0071499 | cellular response to laminar fluid shear stress | 1 | 0.0093288 | KLF2 |
| GO | GO:0036003 | positive regulation of transcription from RNA polymerase II promoter in response to stress | 1 | 0.0093288 | KLF2 |
| GO | GO:0062023 | collagen-containing extracellular matrix | 3 | 0.0095281 | APOE/CTHRC1/SPARCL1 |
| GO | GO:0002576 | platelet degranulation | 2 | 0.0096301 | IGF2/PECAM1 |
| GO | GO:0071356 | cellular response to tumor necrosis factor | 2 | 0.009777 | CCL3/KLF2 |
| GO | GO:0005506 | iron ion binding | 2 | 0.0100736 | CYP1B1/LTF |
| GO | GO:0006954 | inflammatory response | 3 | 0.0103064 | PLA2G2A/CCL3/ACKR1 |
| GO | GO:0101020 | estrogen 16-alpha-hydroxylase activity | 1 | 0.0104889 | CYP1B1 |
| GO | GO:0072678 | T cell migration | 1 | 0.0104889 | S1PR1 |
| GO | GO:0031017 | exocrine pancreas development | 1 | 0.0104889 | IGF2 |
| GO | GO:0014808 | release of sequestered calcium ion into cytosol by sarcoplasmic reticulum | 1 | 0.0104889 | CCL3 |
| GO | GO:0038036 | sphingosine-1-phosphate receptor activity | 1 | 0.0104889 | S1PR1 |
| GO | GO:0035912 | dorsal aorta morphogenesis | 1 | 0.0104889 | HEY1 |
| GO | GO:0001955 | blood vessel maturation | 1 | 0.0104889 | S1PR1 |
| GO | GO:1903980 | positive regulation of microglial cell activation | 1 | 0.0104889 | CCL3 |
| GO | GO:0010544 | negative regulation of platelet activation | 1 | 0.0104889 | APOE |
| GO | GO:0032288 | myelin assembly | 1 | 0.0104889 | CFD |
| GO | GO:0034382 | chylomicron remnant clearance | 1 | 0.0104889 | APOE |
| GO | GO:0044794 | positive regulation by host of viral process | 1 | 0.0104889 | APOE |
| GO | GO:0004930 | G protein-coupled receptor activity | 4 | 0.011045 | S1PR1/GPR34/ACKR1/CALCRL |
| GO | GO:0009887 | animal organ morphogenesis | 2 | 0.0111432 | IGF2/TMEM176B |
| GO | GO:0010873 | positive regulation of cholesterol esterification | 1 | 0.0116477 | APOE |
| GO | GO:0003084 | positive regulation of systemic arterial blood pressure | 1 | 0.0116477 | NR2F2 |
| GO | GO:0034145 | positive regulation of toll-like receptor 4 signaling pathway | 1 | 0.0116477 | LTF |
| GO | GO:0003208 | cardiac ventricle morphogenesis | 1 | 0.0116477 | HEY1 |
| GO | GO:0032680 | regulation of tumor necrosis factor production | 1 | 0.0116477 | LTF |
| GO | GO:0008037 | cell recognition | 1 | 0.0116477 | PECAM1 |
| GO | GO:0045762 | positive regulation of adenylate cyclase activity | 1 | 0.0116477 | CACNA1A |
| GO | GO:0034372 | very-low-density lipoprotein particle remodeling | 1 | 0.0116477 | APOE |
| GO | GO:0070723 | response to cholesterol | 1 | 0.0116477 | CCL3 |
| GO | GO:0046951 | ketone body biosynthetic process | 1 | 0.0116477 | ACSS3 |
| GO | GO:0034371 | chylomicron remodeling | 1 | 0.0116477 | APOE |
| GO | GO:0090382 | phagosome maturation | 1 | 0.0116477 | CFD |
| GO | GO:0070168 | negative regulation of biomineral tissue development | 1 | 0.0116477 | HEY1 |
| GO | GO:0004983 | neuropeptide Y receptor activity | 1 | 0.0116477 | NPY1R |
| GO | GO:0007010 | cytoskeleton organization | 2 | 0.011776 | CCL3/APOE |
| GO | GO:0046983 | protein dimerization activity | 2 | 0.0120982 | APOE/HEY1 |
| GO | GO:0045124 | regulation of bone resorption | 1 | 0.0128051 | S1PR1 |
| GO | GO:0003376 | sphingosine-1-phosphate receptor signaling pathway | 1 | 0.0128051 | S1PR1 |
| GO | GO:0047760 | butyrate-CoA ligase activity | 1 | 0.0128051 | ACSS3 |
| GO | GO:0097267 | omega-hydroxylase P450 pathway | 1 | 0.0128051 | CYP1B1 |
| GO | GO:1903671 | negative regulation of sprouting angiogenesis | 1 | 0.0128051 | KLF2 |
| GO | GO:0008331 | high voltage-gated calcium channel activity | 1 | 0.0128051 | CACNA1A |
| GO | GO:0033629 | negative regulation of cell adhesion mediated by integrin | 1 | 0.0128051 | CYP1B1 |
| GO | GO:0035372 | protein localization to microtubule | 1 | 0.0128051 | CAMP |
| GO | GO:0051247 | positive regulation of protein metabolic process | 1 | 0.0128051 | KLF2 |
| GO | GO:0050795 | regulation of behavior | 1 | 0.0128051 | CCL3 |
| GO | GO:0001775 | cell activation | 1 | 0.0128051 | CCL3 |
| GO | GO:0002021 | response to dietary excess | 1 | 0.0128051 | APOE |
| GO | GO:0034384 | high-density lipoprotein particle clearance | 1 | 0.0128051 | APOE |
| GO | GO:0004888 | transmembrane signaling receptor activity | 2 | 0.0130878 | PECAM1/ACKR1 |
| GO | GO:0043231 | intracellular membrane-bounded organelle | 4 | 0.0136891 | CFD/CYP1B1/S1PR1/PARM1 |
| GO | GO:0061314 | Notch signaling involved in heart development | 1 | 0.0139612 | HEY1 |
| GO | GO:0042982 | amyloid precursor protein metabolic process | 1 | 0.0139612 | APOE |
| GO | GO:0001893 | maternal placenta development | 1 | 0.0139612 | NR2F2 |
| GO | GO:0006707 | cholesterol catabolic process | 1 | 0.0139612 | APOE |
| GO | GO:0034501 | protein localization to kinetochore | 1 | 0.0139612 | CAMP |
| GO | GO:0042582 | azurophil granule | 1 | 0.0139612 | DEFA4 |
| GO | GO:0055064 | chloride ion homeostasis | 1 | 0.0139612 | CA12 |
| GO | GO:0045446 | endothelial cell differentiation | 1 | 0.0139612 | S1PR1 |
| GO | GO:0034378 | chylomicron assembly | 1 | 0.0139612 | APOE |
| GO | GO:0010818 | T cell chemotaxis | 1 | 0.0139612 | CCL3 |
| GO | GO:0019068 | virion assembly | 1 | 0.0139612 | APOE |
| GO | GO:0051315 | attachment of mitotic spindle microtubules to kinetochore | 1 | 0.0139612 | CAMP |
| GO | GO:0031665 | negative regulation of lipopolysaccharide-mediated signaling pathway | 1 | 0.0139612 | LTF |
| GO | GO:0000737 | DNA catabolic process, endonucleolytic | 1 | 0.0139612 | DNASE1L3 |
| GO | GO:0099149 | regulation of postsynaptic neurotransmitter receptor internalization | 1 | 0.015116 | CACNA1A |
| GO | GO:0044327 | dendritic spine head | 1 | 0.015116 | CACNA1A |
| GO | GO:0060674 | placenta blood vessel development | 1 | 0.015116 | NR2F2 |
| GO | GO:0071257 | cellular response to electrical stimulus | 1 | 0.015116 | CACNA1A |
| GO | GO:0033700 | phospholipid efflux | 1 | 0.015116 | APOE |
| GO | GO:0032780 | negative regulation of ATPase activity | 1 | 0.015116 | LTF |
| GO | GO:0050927 | positive regulation of positive chemotaxis | 1 | 0.015116 | S1PR1 |
| GO | GO:0043922 | negative regulation by host of viral transcription | 1 | 0.015116 | CCL3 |
| GO | GO:0045028 | G protein-coupled purinergic nucleotide receptor activity | 1 | 0.015116 | GPR34 |
| GO | GO:0072359 | circulatory system development | 1 | 0.015116 | HEY1 |
| GO | GO:1900272 | negative regulation of long-term synaptic potentiation | 1 | 0.015116 | APOE |
| GO | GO:0071498 | cellular response to fluid shear stress | 1 | 0.015116 | KLF2 |
| GO | GO:0005737 | cytoplasm | 11 | 0.0160447 | RGS5/CCL3/APOE/CALCRL/CACNA1A/S1PR1/CAMP/CAPN6/LTF/CTHRC1/HEY1 |
| GO | GO:0006898 | receptor-mediated endocytosis | 2 | 0.0160755 | CFD/APOE |
| GO | GO:0008201 | heparin binding | 2 | 0.0160755 | APOE/LTF |
| GO | GO:0035641 | locomotory exploration behavior | 1 | 0.0162695 | APOE |
| GO | GO:0071383 | cellular response to steroid hormone stimulus | 1 | 0.0162695 | TFPI |
| GO | GO:0060317 | cardiac epithelial to mesenchymal transition | 1 | 0.0162695 | HEY1 |
| GO | GO:0000302 | response to reactive oxygen species | 1 | 0.0162695 | APOE |
| GO | GO:0042581 | specific granule | 1 | 0.0162695 | LTF |
| GO | GO:0006809 | nitric oxide biosynthetic process | 1 | 0.0162695 | CYP1B1 |
| GO | GO:0060411 | cardiac septum morphogenesis | 1 | 0.0162695 | HEY1 |
| GO | GO:2000678 | negative regulation of transcription regulatory region DNA binding | 1 | 0.0162695 | HEY1 |
| GO | GO:0034380 | high-density lipoprotein particle assembly | 1 | 0.0162695 | APOE |
| GO | GO:0042627 | chylomicron | 1 | 0.0162695 | APOE |
| GO | GO:0000122 | negative regulation of transcription by RNA polymerase II | 4 | 0.016382 | IGF2/NR2F2/KLF2/HEY1 |
| GO | GO:0051897 | positive regulation of protein kinase B signaling | 2 | 0.0166304 | IGF2/CCL3 |
| GO | GO:0004252 | serine-type endopeptidase activity | 2 | 0.0171934 | LTF/GZMA |
| GO | GO:0030516 | regulation of axon extension | 1 | 0.0174216 | APOE |
| GO | GO:0060707 | trophoblast giant cell differentiation | 1 | 0.0174216 | NR2F2 |
| GO | GO:0035589 | G protein-coupled purinergic nucleotide receptor signaling pathway | 1 | 0.0174216 | GPR34 |
| GO | GO:0036092 | phosphatidylinositol-3-phosphate biosynthetic process | 1 | 0.0174216 | CFD |
| GO | GO:0090181 | regulation of cholesterol metabolic process | 1 | 0.0174216 | APOE |
| GO | GO:0008631 | intrinsic apoptotic signaling pathway in response to oxidative stress | 1 | 0.0174216 | CYP1B1 |
| GO | GO:2000785 | regulation of autophagosome assembly | 1 | 0.0174216 | CFD |
| GO | GO:0051146 | striated muscle cell differentiation | 1 | 0.0174216 | IGF2 |
| GO | GO:1902430 | negative regulation of amyloid-beta formation | 1 | 0.0174216 | APOE |
| GO | GO:0034362 | low-density lipoprotein particle | 1 | 0.0174216 | APOE |
| GO | GO:0001886 | endothelial cell morphogenesis | 1 | 0.0174216 | PECAM1 |
| GO | GO:0048246 | macrophage chemotaxis | 1 | 0.0174216 | CCL3 |
| GO | GO:1904645 | response to amyloid-beta | 1 | 0.0174216 | CACNA1A |
| GO | GO:0040029 | regulation of gene expression, epigenetic | 1 | 0.0174216 | KLF2 |
| GO | GO:0090090 | negative regulation of canonical Wnt signaling pathway | 2 | 0.0185382 | APOE/CTHRC1 |
| GO | GO:1901653 | cellular response to peptide | 1 | 0.0185724 | KLF2 |
| GO | GO:0043249 | erythrocyte maturation | 1 | 0.0185724 | KLF2 |
| GO | GO:0003184 | pulmonary valve morphogenesis | 1 | 0.0185724 | HEY1 |
| GO | GO:0042574 | retinal metabolic process | 1 | 0.0185724 | CYP1B1 |
| GO | GO:0060347 | heart trabecula formation | 1 | 0.0185724 | HEY1 |
| GO | GO:0051044 | positive regulation of membrane protein ectodomain proteolysis | 1 | 0.0185724 | APOE |
| GO | GO:0071682 | endocytic vesicle lumen | 1 | 0.0185724 | APOE |
| GO | GO:0001934 | positive regulation of protein phosphorylation | 2 | 0.0189304 | IGF2/PECAM1 |
| GO | GO:0043312 | neutrophil degranulation | 3 | 0.0191695 | PECAM1/DEFA4/LTF |
| GO | GO:0045725 | positive regulation of glycogen biosynthetic process | 1 | 0.0197219 | IGF2 |
| GO | GO:0004089 | carbonate dehydratase activity | 1 | 0.0197219 | CA12 |
| GO | GO:0006349 | regulation of gene expression by genetic imprinting | 1 | 0.0197219 | IGF2 |
| GO | GO:0043395 | heparan sulfate proteoglycan binding | 1 | 0.0197219 | APOE |
| GO | GO:0031090 | organelle membrane | 1 | 0.0197219 | TFPI |
| GO | GO:0047498 | calcium-dependent phospholipase A2 activity | 1 | 0.0197219 | PLA2G2A |
| GO | GO:1905564 | positive regulation of vascular endothelial cell proliferation | 1 | 0.0197219 | IGF2 |
| GO | GO:0004698 | calcium-dependent protein kinase C activity | 1 | 0.0197219 | CCL3 |
| GO | GO:0015909 | long-chain fatty acid transport | 1 | 0.0197219 | APOE |
| GO | GO:0016075 | rRNA catabolic process | 1 | 0.0197219 | ERN2 |
| GO | GO:0006309 | apoptotic DNA fragmentation | 1 | 0.0197219 | DNASE1L3 |
| GO | GO:0046470 | phosphatidylcholine metabolic process | 1 | 0.0197219 | PLA2G2A |
| GO | GO:0005159 | insulin-like growth factor receptor binding | 1 | 0.0197219 | IGF2 |
| GO | GO:0055089 | fatty acid homeostasis | 1 | 0.0197219 | APOE |
| GO | GO:0030502 | negative regulation of bone mineralization | 1 | 0.0197219 | CCL3 |
| GO | GO:0046889 | positive regulation of lipid biosynthetic process | 1 | 0.0197219 | APOE |
| GO | GO:0005911 | cell-cell junction | 2 | 0.0199262 | PECAM1/CFD |
| GO | GO:0090280 | positive regulation of calcium ion import | 1 | 0.0208701 | CCL3 |
| GO | GO:0048168 | regulation of neuronal synaptic plasticity | 1 | 0.0208701 | APOE |
| GO | GO:0031092 | platelet alpha granule membrane | 1 | 0.0208701 | PECAM1 |
| GO | GO:0004198 | calcium-dependent cysteine-type endopeptidase activity | 1 | 0.0208701 | CAPN6 |
| GO | GO:0048245 | eosinophil chemotaxis | 1 | 0.0208701 | CCL3 |
| GO | GO:0060122 | inner ear receptor cell stereocilium organization | 1 | 0.0208701 | CTHRC1 |
| GO | GO:0043691 | reverse cholesterol transport | 1 | 0.0208701 | APOE |
| GO | GO:0090501 | RNA phosphodiester bond hydrolysis | 1 | 0.0208701 | ERN2 |
| GO | GO:0043083 | synaptic cleft | 1 | 0.0208701 | APOE |
| GO | GO:0050850 | positive regulation of calcium-mediated signaling | 1 | 0.0208701 | CCL3 |
| GO | GO:0010744 | positive regulation of macrophage derived foam cell differentiation | 1 | 0.022017 | PLA2G2A |
| GO | GO:0003203 | endocardial cushion morphogenesis | 1 | 0.022017 | HEY1 |
| GO | GO:0034375 | high-density lipoprotein particle remodeling | 1 | 0.022017 | APOE |
| GO | GO:0036149 | phosphatidylinositol acyl-chain remodeling | 1 | 0.022017 | PLA2G2A |
| GO | GO:0016209 | antioxidant activity | 1 | 0.022017 | APOE |
| GO | GO:0061136 | regulation of proteasomal protein catabolic process | 1 | 0.022017 | APOE |
| GO | GO:0036148 | phosphatidylglycerol acyl-chain remodeling | 1 | 0.022017 | PLA2G2A |
| GO | GO:0061028 | establishment of endothelial barrier | 1 | 0.022017 | PECAM1 |
| GO | GO:2000377 | regulation of reactive oxygen species metabolic process | 1 | 0.022017 | CYP1B1 |
| GO | GO:0005887 | integral component of plasma membrane | 5 | 0.0224146 | PECAM1/NPY1R/GPR34/TSPAN7/CALCRL |
| GO | GO:0007263 | nitric oxide mediated signal transduction | 1 | 0.0231625 | APOE |
| GO | GO:0043254 | regulation of protein-containing complex assembly | 1 | 0.0231625 | APOE |
| GO | GO:0046907 | intracellular transport | 1 | 0.0231625 | APOE |
| GO | GO:0019226 | transmission of nerve impulse | 1 | 0.0231625 | S1PR1 |
| GO | GO:0001701 | in utero embryonic development | 2 | 0.023905 | IGF2/KLF2 |
| GO | GO:0001892 | embryonic placenta development | 1 | 0.0243068 | IGF2 |
| GO | GO:0050709 | negative regulation of protein secretion | 1 | 0.0243068 | APOE |
| GO | GO:0019373 | epoxygenase P450 pathway | 1 | 0.0243068 | CYP1B1 |
| GO | GO:0019222 | regulation of metabolic process | 1 | 0.0243068 | S1PR1 |
| GO | GO:0010875 | positive regulation of cholesterol efflux | 1 | 0.0243068 | APOE |
| GO | GO:0120020 | cholesterol transfer activity | 1 | 0.0243068 | APOE |
| GO | GO:0034361 | very-low-density lipoprotein particle | 1 | 0.0243068 | APOE |
| GO | GO:0030500 | regulation of bone mineralization | 1 | 0.0243068 | S1PR1 |
| GO | GO:0030520 | intracellular estrogen receptor signaling pathway | 1 | 0.0243068 | DEFA3 |
| GO | GO:0042311 | vasodilation | 1 | 0.0243068 | APOE |
| GO | GO:0008210 | estrogen metabolic process | 1 | 0.0243068 | CYP1B1 |
| GO | GO:0016836 | hydro-lyase activity | 1 | 0.0243068 | CA12 |
| GO | GO:0005789 | endoplasmic reticulum membrane | 4 | 0.0245066 | PLA2G2A/CYP1B1/ERN2/GIMAP1 |
| GO | GO:0045892 | negative regulation of transcription, DNA-templated | 3 | 0.0252079 | NR2F2/ERN2/HEY1 |
| GO | GO:0019934 | cGMP-mediated signaling | 1 | 0.0254497 | APOE |
| GO | GO:0045088 | regulation of innate immune response | 1 | 0.0254497 | APOE |
| GO | GO:0016712 | oxidoreductase activity, acting on paired donors, with incorporation or reduction of molecular oxygen, reduced flavin or flavoprotein as one donor, and incorporation of one atom of oxygen | 1 | 0.0254497 | CYP1B1 |
| GO | GO:0060716 | labyrinthine layer blood vessel development | 1 | 0.0254497 | HEY1 |
| GO | GO:0051000 | positive regulation of nitric-oxide synthase activity | 1 | 0.0254497 | APOE |
| GO | GO:0036150 | phosphatidylserine acyl-chain remodeling | 1 | 0.0254497 | PLA2G2A |
| GO | GO:0004540 | ribonuclease activity | 1 | 0.0254497 | ERN2 |
| GO | GO:0050750 | low-density lipoprotein particle receptor binding | 1 | 0.0254497 | APOE |
| GO | GO:0045807 | positive regulation of endocytosis | 1 | 0.0254497 | APOE |
| GO | GO:0043537 | negative regulation of blood vessel endothelial cell migration | 1 | 0.0254497 | APOE |
| GO | GO:0010629 | negative regulation of gene expression | 2 | 0.02567 | CCL3/APOE |
| GO | GO:0010467 | gene expression | 1 | 0.0265914 | APOE |
| GO | GO:0019957 | C-C chemokine binding | 1 | 0.0265914 | ACKR1 |
| GO | GO:0051899 | membrane depolarization | 1 | 0.0265914 | CACNA1A |
| GO | GO:0034364 | high-density lipoprotein particle | 1 | 0.0265914 | APOE |
| GO | GO:0004672 | protein kinase activity | 2 | 0.0270278 | CCL3/ERN2 |
| GO | GO:0032438 | melanosome organization | 1 | 0.0277317 | CFD |
| GO | GO:0060999 | positive regulation of dendritic spine development | 1 | 0.0277317 | APOE |
| GO | GO:0005158 | insulin receptor binding | 1 | 0.0277317 | IGF2 |
| GO | GO:0070509 | calcium ion import | 1 | 0.0277317 | CACNA1A |
| GO | GO:0016125 | sterol metabolic process | 1 | 0.0277317 | CYP1B1 |
| GO | GO:0048844 | artery morphogenesis | 1 | 0.0277317 | APOE |
| GO | GO:0030595 | leukocyte chemotaxis | 1 | 0.0277317 | S1PR1 |
| GO | GO:0033344 | cholesterol efflux | 1 | 0.0277317 | APOE |
| GO | GO:0010008 | endosome membrane | 2 | 0.0279488 | CFD/PARM1 |
| GO | GO:0050482 | arachidonic acid secretion | 1 | 0.0288707 | PLA2G2A |
| GO | GO:0090103 | cochlea morphogenesis | 1 | 0.0288707 | CTHRC1 |
| GO | GO:0042104 | positive regulation of activated T cell proliferation | 1 | 0.0288707 | IGF2 |
| GO | GO:0046628 | positive regulation of insulin receptor signaling pathway | 1 | 0.0288707 | IGF2 |
| GO | GO:0070330 | aromatase activity | 1 | 0.0300084 | CYP1B1 |
| GO | GO:0044291 | cell-cell contact zone | 1 | 0.0300084 | PECAM1 |
| GO | GO:0001972 | retinoic acid binding | 1 | 0.0300084 | NR2F2 |
| GO | GO:0036152 | phosphatidylethanolamine acyl-chain remodeling | 1 | 0.0300084 | PLA2G2A |
| GO | GO:0045671 | negative regulation of osteoclast differentiation | 1 | 0.0300084 | CCL3 |
| GO | GO:0099560 | synaptic membrane adhesion | 1 | 0.0300084 | SPARCL1 |
| GO | GO:0007271 | synaptic transmission, cholinergic | 1 | 0.0300084 | APOE |
| GO | GO:0007420 | brain development | 2 | 0.0305461 | CACNA1A/S1PR1 |
| GO | GO:0016477 | cell migration | 2 | 0.0307868 | S1PR1/CTHRC1 |
| GO | GO:0102567 | phospholipase A2 activity (consuming 1,2-dipalmitoylphosphatidylcholine) | 1 | 0.0311448 | PLA2G2A |
| GO | GO:0051497 | negative regulation of stress fiber assembly | 1 | 0.0311448 | S1PR1 |
| GO | GO:0030522 | intracellular receptor signaling pathway | 1 | 0.0311448 | NR2F2 |
| GO | GO:0006259 | DNA metabolic process | 1 | 0.0311448 | DNASE1L3 |
| GO | GO:0102568 | phospholipase A2 activity consuming 1,2-dioleoylphosphatidylethanolamine) | 1 | 0.0311448 | PLA2G2A |
| GO | GO:0051928 | positive regulation of calcium ion transport | 1 | 0.0322799 | CCL3 |
| GO | GO:0040014 | regulation of multicellular organism growth | 1 | 0.0322799 | NPY1R |
| GO | GO:0005891 | voltage-gated calcium channel complex | 1 | 0.0322799 | CACNA1A |
| GO | GO:0060349 | bone morphogenesis | 1 | 0.0322799 | LTF |
| GO | GO:0005319 | lipid transporter activity | 1 | 0.0322799 | APOE |
| GO | GO:0000793 | condensed chromosome | 1 | 0.0322799 | CAMP |
| GO | GO:0030154 | cell differentiation | 3 | 0.0329885 | TMEM176B/NR2F2/HEY1 |
| GO | GO:0032809 | neuronal cell body membrane | 1 | 0.0334137 | CACNA1A |
| GO | GO:0048020 | CCR chemokine receptor binding | 1 | 0.0334137 | CCL3 |
| GO | GO:0050840 | extracellular matrix binding | 1 | 0.0334137 | SPARCL1 |
| GO | GO:0004623 | phospholipase A2 activity | 1 | 0.0334137 | PLA2G2A |
| GO | GO:0045840 | positive regulation of mitotic nuclear division | 1 | 0.0334137 | IGF2 |
| GO | GO:0070328 | triglyceride homeostasis | 1 | 0.0334137 | APOE |
| GO | GO:0010575 | positive regulation of vascular endothelial growth factor production | 1 | 0.0334137 | CYP1B1 |
| GO | GO:0036151 | phosphatidylcholine acyl-chain remodeling | 1 | 0.0334137 | PLA2G2A |
| GO | GO:0051603 | proteolysis involved in cellular protein catabolic process | 1 | 0.0334137 | GZMA |
| GO | GO:0003700 | DNA-binding transcription factor activity | 3 | 0.0345366 | NR2F2/KLF2/HEY1 |
| GO | GO:0019369 | arachidonic acid metabolic process | 1 | 0.0345462 | CYP1B1 |
| GO | GO:0046427 | positive regulation of receptor signaling pathway via JAK-STAT | 1 | 0.0345462 | CYP1B1 |
| GO | GO:0045736 | negative regulation of cyclin-dependent protein serine/threonine kinase activity | 1 | 0.0345462 | NR2F2 |
| GO | GO:0051930 | regulation of sensory perception of pain | 1 | 0.0345462 | CCL3 |
| GO | GO:0007631 | feeding behavior | 1 | 0.0345462 | NPY1R |
| GO | GO:0007159 | leukocyte cell-cell adhesion | 1 | 0.0345462 | PECAM1 |
| GO | GO:0017147 | Wnt-protein binding | 1 | 0.0356774 | CTHRC1 |
| GO | GO:0032590 | dendrite membrane | 1 | 0.0356774 | CACNA1A |
| GO | GO:0051493 | regulation of cytoskeleton organization | 1 | 0.0356774 | CAPN6 |
| GO | GO:0043392 | negative regulation of DNA binding | 1 | 0.0356774 | GZMA |
| GO | GO:0000139 | Golgi membrane | 3 | 0.0358311 | CFD/GIMAP1/PARM1 |
| GO | GO:0090543 | Flemming body | 1 | 0.0368073 | CAMP |
| GO | GO:0035633 | maintenance of blood-brain barrier | 1 | 0.0368073 | PECAM1 |
| GO | GO:0090314 | positive regulation of protein targeting to membrane | 1 | 0.0368073 | CACNA1A |
| GO | GO:0001578 | microtubule bundle formation | 1 | 0.0379359 | CAPN6 |
| GO | GO:0019825 | oxygen binding | 1 | 0.0379359 | CYP1B1 |
| GO | GO:0048247 | lymphocyte chemotaxis | 1 | 0.0379359 | CCL3 |
| GO | GO:0030669 | clathrin-coated endocytic vesicle membrane | 1 | 0.0379359 | APOE |
| GO | GO:0042056 | chemoattractant activity | 1 | 0.0390632 | CCL3 |
| GO | GO:0051482 | positive regulation of cytosolic calcium ion concentration involved in phospholipase C-activating G protein-coupled signaling pathway | 1 | 0.0390632 | S1PR1 |
| GO | GO:0004519 | endonuclease activity | 1 | 0.0390632 | ERN2 |
| GO | GO:0060173 | limb development | 1 | 0.0390632 | NR2F2 |
| GO | GO:0032839 | dendrite cytoplasm | 1 | 0.0390632 | CACNA1A |
| GO | GO:0030032 | lamellipodium assembly | 1 | 0.0390632 | S1PR1 |
| GO | GO:0040018 | positive regulation of multicellular organism growth | 1 | 0.0390632 | IGF2 |
| GO | GO:0006641 | triglyceride metabolic process | 1 | 0.0390632 | APOE |
| GO | GO:0004521 | endoribonuclease activity | 1 | 0.0390632 | ERN2 |
| GO | GO:0050767 | regulation of neurogenesis | 1 | 0.0390632 | HEY1 |
| GO | GO:0001530 | lipopolysaccharide binding | 1 | 0.0390632 | LTF |
| GO | GO:0001817 | regulation of cytokine production | 1 | 0.0390632 | LTF |
| GO | GO:0031663 | lipopolysaccharide-mediated signaling pathway | 1 | 0.0401892 | CCL3 |
| GO | GO:0007616 | long-term memory | 1 | 0.0401892 | APOE |
| GO | GO:0004869 | cysteine-type endopeptidase inhibitor activity | 1 | 0.0401892 | LTF |
| GO | GO:0060412 | ventricular septum morphogenesis | 1 | 0.0401892 | HEY1 |
| GO | GO:0034504 | protein localization to nucleus | 1 | 0.0401892 | CFD |
| GO | GO:0070059 | intrinsic apoptotic signaling pathway in response to endoplasmic reticulum stress | 1 | 0.0401892 | ERN2 |
| GO | GO:0051973 | positive regulation of telomerase activity | 1 | 0.0401892 | PARM1 |
| GO | GO:0004497 | monooxygenase activity | 1 | 0.0424374 | CYP1B1 |
| GO | GO:0098742 | cell-cell adhesion via plasma-membrane adhesion molecules | 1 | 0.0424374 | PECAM1 |
| GO | GO:0006730 | one-carbon metabolic process | 1 | 0.0424374 | CA12 |
| GO | GO:0008219 | cell death | 1 | 0.0424374 | CACNA1A |
| GO | GO:0048018 | receptor ligand activity | 1 | 0.0424374 | IGF2 |
| GO | GO:0031226 | intrinsic component of plasma membrane | 1 | 0.0435595 | S1PR1 |
| GO | GO:0019221 | cytokine-mediated signaling pathway | 2 | 0.0437522 | CCL3/S1PR1 |
| GO | GO:0005245 | voltage-gated calcium channel activity | 1 | 0.0446804 | CACNA1A |
| GO | GO:0043491 | protein kinase B signaling | 1 | 0.0446804 | CCL3 |
| GO | GO:0005109 | frizzled binding | 1 | 0.0446804 | CTHRC1 |
| GO | GO:0045746 | negative regulation of Notch signaling pathway | 1 | 0.0446804 | HEY1 |
| GO | GO:0007166 | cell surface receptor signaling pathway | 2 | 0.0448642 | PECAM1/CALCRL |
| GO | GO:0009968 | negative regulation of signal transduction | 1 | 0.0458 | RGS5 |
| GO | GO:0009566 | fertilization | 1 | 0.0458 | NR2F2 |
| GO | GO:0043407 | negative regulation of MAP kinase activity | 1 | 0.0458 | APOE |
| GO | GO:0045429 | positive regulation of nitric oxide biosynthetic process | 1 | 0.0458 | KLF2 |
| GO | GO:0045944 | positive regulation of transcription by RNA polymerase II | 4 | 0.0467304 | IGF2/S1PR1/KLF2/HEY1 |
| GO | GO:0005768 | endosome | 2 | 0.0468355 | S1PR1/CALCRL |
| GO | GO:0007257 | activation of JUN kinase activity | 1 | 0.0469183 | ERN2 |
| GO | GO:0032715 | negative regulation of interleukin-6 production | 1 | 0.0469183 | KLF2 |
| GO | GO:0004866 | endopeptidase inhibitor activity | 1 | 0.0469183 | TFPI |
| GO | GO:0001772 | immunological synapse | 1 | 0.0469183 | GZMA |
| GO | GO:0005788 | endoplasmic reticulum lumen | 2 | 0.04769 | APOE/SPARCL1 |
| GO | GO:1904646 | cellular response to amyloid-beta | 1 | 0.0480353 | CACNA1A |
| GO | GO:0045071 | negative regulation of viral genome replication | 1 | 0.0480353 | LTF |
| GO | GO:0015701 | bicarbonate transport | 1 | 0.0480353 | CA12 |
| GO | GO:0001895 | retina homeostasis | 1 | 0.0480353 | LTF |
| GO | GO:0048471 | perinuclear region of cytoplasm | 3 | 0.0485942 | PLA2G2A/CAPN6/CFD |
| GO | GO:0031623 | receptor internalization | 1 | 0.049151 | CALCRL |
| GO | GO:0032570 | response to progesterone | 1 | 0.049151 | CSN1S1 |
| GO | GO:0004879 | nuclear receptor activity | 1 | 0.049151 | NR2F2 |

**Abbreviation**: GO, gene ontology; KEGG, Kyoto Encyclopedia of Genes and Genomes; Co-DEGs, common differentially expressed genes.
