# Supplementary material for: LTF induces senescence and degeneration in the meniscus via the NF-κB signaling pathway: A study based on integrated bioinformatics analysis and experimental validation
Source: Front Mol Biosci. 2023 Apr 24;10:1134253. doi: 10.3389/fmolb.2023.1134253 (PMC10164984; doi:10.3389/fmolb.2023.1134253)
Supplement: Supplementary file 7 [file Table6.DOCX]

**Table S6. Detailed results of the KEGG and GO enrichment analysis of the shared hub genes.**

| **Group** | **ID** | **Description** | **Count** | ***P* Value** | **Genes** |
| --- | --- | --- | --- | --- | --- |
| KEGG | hsa05144 | Malaria | 2 | 5.92E-05 | PECAM1/ACKR1 |
| KEGG | hsa04068 | FoxO signaling pathway | 2 | 0.0004099 | S1PR1/KLF2 |
| KEGG | hsa05418 | Fluid shear stress and atherosclerosis | 2 | 0.0004537 | PECAM1/KLF2 |
| KEGG | hsa04080 | Neuroactive ligand-receptor interaction | 2 | 0.0025869 | S1PR1/GZMA |
| KEGG | hsa04979 | Cholesterol metabolism | 1 | 0.011634 | APOE |
| KEGG | hsa05132 | Salmonella infection | 1 | 0.0190976 | CCL3 |
| KEGG | hsa05323 | Rheumatoid arthritis | 1 | 0.0208994 | CCL3 |
| KEGG | hsa04061 | Viral protein interaction with cytokine and cytokine receptor | 1 | 0.0229229 | CCL3 |
| KEGG | hsa05142 | Chagas disease (American trypanosomiasis) | 1 | 0.0235965 | CCL3 |
| KEGG | hsa04620 | Toll-like receptor signaling pathway | 1 | 0.023821 | CCL3 |
| KEGG | hsa04670 | Leukocyte transendothelial migration | 1 | 0.0256151 | PECAM1 |
| KEGG | hsa04071 | Sphingolipid signaling pathway | 1 | 0.0271825 | S1PR1 |
| KEGG | hsa04371 | Apelin signaling pathway | 1 | 0.0312027 | KLF2 |
| KEGG | hsa04514 | Cell adhesion molecules (CAMs) | 1 | 0.0332073 | PECAM1 |
| KEGG | hsa05010 | Alzheimer disease | 1 | 0.0387562 | APOE |
| KEGG | hsa04062 | Chemokine signaling pathway | 1 | 0.0429544 | CCL3 |
| GO | GO:0044267 | cellular protein metabolic process | 3 | 1.09E-05 | APOE/LTF/SPARCL1 |
| GO | GO:0005615 | extracellular space | 5 | 1.14E-05 | SPARCL1/CCL3/APOE/LTF/PECAM1 |
| GO | GO:0030335 | positive regulation of cell migration | 3 | 1.63E-05 | CCL3/S1PR1/PECAM1 |
| GO | GO:0005576 | extracellular region | 5 | 2.47E-05 | SPARCL1/CCL3/APOE/LTF/GZMA |
| GO | GO:0007186 | G protein-coupled receptor signaling pathway | 4 | 7.83E-05 | CCL3/S1PR1/APOE/ACKR1 |
| GO | GO:0070098 | chemokine-mediated signaling pathway | 2 | 0.0001088 | CCL3/ACKR1 |
| GO | GO:0071347 | cellular response to interleukin-1 | 2 | 0.0001537 | CCL3/KLF2 |
| GO | GO:0006874 | cellular calcium ion homeostasis | 2 | 0.0002286 | CCL3/APOE |
| GO | GO:0006935 | chemotaxis | 2 | 0.0003345 | CCL3/S1PR1 |
| GO | GO:0071356 | cellular response to tumor necrosis factor | 2 | 0.0003684 | CCL3/KLF2 |
| GO | GO:0042803 | protein homodimerization activity | 3 | 0.0003735 | PECAM1/APOE/GZMA |
| GO | GO:0005515 | protein binding | 8 | 0.0004355 | CCL3/APOE/GZMA/PECAM1/S1PR1/SPARCL1/KLF2/LTF |
| GO | GO:0007010 | cytoskeleton organization | 2 | 0.0004473 | CCL3/APOE |
| GO | GO:0004888 | transmembrane signaling receptor activity | 2 | 0.0004996 | PECAM1/ACKR1 |
| GO | GO:0008201 | heparin binding | 2 | 0.0006201 | APOE/LTF |
| GO | GO:0004252 | serine-type endopeptidase activity | 2 | 0.0006657 | LTF/GZMA |
| GO | GO:0070374 | positive regulation of ERK1 and ERK2 cascade | 2 | 0.000991 | CCL3/APOE |
| GO | GO:0010629 | negative regulation of gene expression | 2 | 0.0010194 | CCL3/APOE |
| GO | GO:0045121 | membrane raft | 2 | 0.0012921 | PECAM1/S1PR1 |
| GO | GO:0001525 | angiogenesis | 2 | 0.0013028 | PECAM1/S1PR1 |
| GO | GO:0048386 | positive regulation of retinoic acid receptor signaling pathway | 1 | 0.001375 | KLF2 |
| GO | GO:0071621 | granulocyte chemotaxis | 1 | 0.001375 | CCL3 |
| GO | GO:0034363 | intermediate-density lipoprotein particle | 1 | 0.001375 | APOE |
| GO | GO:0032269 | negative regulation of cellular protein metabolic process | 1 | 0.001375 | APOE |
| GO | GO:0045541 | negative regulation of cholesterol biosynthetic process | 1 | 0.001375 | APOE |
| GO | GO:0019732 | antifungal humoral response | 1 | 0.001375 | LTF |
| GO | GO:0017038 | protein import | 1 | 0.001375 | APOE |
| GO | GO:0051246 | regulation of protein metabolic process | 1 | 0.001375 | APOE |
| GO | GO:0042159 | lipoprotein catabolic process | 1 | 0.001375 | APOE |
| GO | GO:0042158 | lipoprotein biosynthetic process | 1 | 0.001375 | APOE |
| GO | GO:2001205 | negative regulation of osteoclast development | 1 | 0.001375 | LTF |
| GO | GO:1905907 | negative regulation of amyloid fibril formation | 1 | 0.001375 | APOE |
| GO | GO:1902732 | positive regulation of chondrocyte proliferation | 1 | 0.001375 | LTF |
| GO | GO:0060509 | type I pneumocyte differentiation | 1 | 0.001375 | KLF2 |
| GO | GO:2000117 | negative regulation of cysteine-type endopeptidase activity | 1 | 0.001375 | LTF |
| GO | GO:0031726 | CCR1 chemokine receptor binding | 1 | 0.001375 | CCL3 |
| GO | GO:0035696 | monocyte extravasation | 1 | 0.001375 | PECAM1 |
| GO | GO:1902991 | regulation of amyloid precursor protein catabolic process | 1 | 0.001375 | APOE |
| GO | GO:0051354 | negative regulation of oxidoreductase activity | 1 | 0.001604 | GZMA |
| GO | GO:0048525 | negative regulation of viral process | 1 | 0.001604 | LTF |
| GO | GO:0072672 | neutrophil extravasation | 1 | 0.001604 | PECAM1 |
| GO | GO:2000503 | positive regulation of natural killer cell chemotaxis | 1 | 0.001604 | CCL3 |
| GO | GO:0060228 | phosphatidylcholine-sterol O-acyltransferase activator activity | 1 | 0.001604 | APOE |
| GO | GO:0043615 | astrocyte cell migration | 1 | 0.001604 | CCL3 |
| GO | GO:0023052 | signaling | 1 | 0.001604 | CCL3 |
| GO | GO:0034447 | very-low-density lipoprotein particle clearance | 1 | 0.001604 | APOE |
| GO | GO:0005769 | early endosome | 2 | 0.0016318 | APOE/ACKR1 |
| GO | GO:0019221 | cytokine-mediated signaling pathway | 2 | 0.0018159 | CCL3/S1PR1 |
| GO | GO:0061384 | heart trabecula morphogenesis | 1 | 0.0018329 | S1PR1 |
| GO | GO:1904141 | positive regulation of microglial cell migration | 1 | 0.0018329 | CCL3 |
| GO | GO:0031730 | CCR5 chemokine receptor binding | 1 | 0.0018329 | CCL3 |
| GO | GO:0016004 | phospholipase activator activity | 1 | 0.0018329 | CCL3 |
| GO | GO:1900223 | positive regulation of amyloid-beta clearance | 1 | 0.0018329 | APOE |
| GO | GO:0071813 | lipoprotein particle binding | 1 | 0.0018329 | APOE |
| GO | GO:0032489 | regulation of Cdc42 protein signal transduction | 1 | 0.0018329 | APOE |
| GO | GO:0071499 | cellular response to laminar fluid shear stress | 1 | 0.0018329 | KLF2 |
| GO | GO:0036003 | positive regulation of transcription from RNA polymerase II promoter in response to stress | 1 | 0.0018329 | KLF2 |
| GO | GO:0005788 | endoplasmic reticulum lumen | 2 | 0.0019964 | APOE/SPARCL1 |
| GO | GO:0072678 | T cell migration | 1 | 0.0020619 | S1PR1 |
| GO | GO:0051673 | membrane disruption in other organism | 1 | 0.0020619 | LTF |
| GO | GO:0014808 | release of sequestered calcium ion into cytosol by sarcoplasmic reticulum | 1 | 0.0020619 | CCL3 |
| GO | GO:0038036 | sphingosine-1-phosphate receptor activity | 1 | 0.0020619 | S1PR1 |
| GO | GO:0001955 | blood vessel maturation | 1 | 0.0020619 | S1PR1 |
| GO | GO:1903980 | positive regulation of microglial cell activation | 1 | 0.0020619 | CCL3 |
| GO | GO:0010544 | negative regulation of platelet activation | 1 | 0.0020619 | APOE |
| GO | GO:0044794 | positive regulation by host of viral process | 1 | 0.0020619 | APOE |
| GO | GO:0034382 | chylomicron remnant clearance | 1 | 0.0020619 | APOE |
| GO | GO:0043547 | positive regulation of GTPase activity | 2 | 0.0021439 | CCL3/S1PR1 |
| GO | GO:0010873 | positive regulation of cholesterol esterification | 1 | 0.0022907 | APOE |
| GO | GO:0034145 | positive regulation of toll-like receptor 4 signaling pathway | 1 | 0.0022907 | LTF |
| GO | GO:0032680 | regulation of tumor necrosis factor production | 1 | 0.0022907 | LTF |
| GO | GO:0008037 | cell recognition | 1 | 0.0022907 | PECAM1 |
| GO | GO:0034372 | very-low-density lipoprotein particle remodeling | 1 | 0.0022907 | APOE |
| GO | GO:0070723 | response to cholesterol | 1 | 0.0022907 | CCL3 |
| GO | GO:0034371 | chylomicron remodeling | 1 | 0.0022907 | APOE |
| GO | GO:0045124 | regulation of bone resorption | 1 | 0.0025195 | S1PR1 |
| GO | GO:0003376 | sphingosine-1-phosphate receptor signaling pathway | 1 | 0.0025195 | S1PR1 |
| GO | GO:0051247 | positive regulation of protein metabolic process | 1 | 0.0025195 | KLF2 |
| GO | GO:0050795 | regulation of behavior | 1 | 0.0025195 | CCL3 |
| GO | GO:0001775 | cell activation | 1 | 0.0025195 | CCL3 |
| GO | GO:0002021 | response to dietary excess | 1 | 0.0025195 | APOE |
| GO | GO:1903671 | negative regulation of sprouting angiogenesis | 1 | 0.0025195 | KLF2 |
| GO | GO:0034384 | high-density lipoprotein particle clearance | 1 | 0.0025195 | APOE |
| GO | GO:0043687 | post-translational protein modification | 2 | 0.002647 | APOE/SPARCL1 |
| GO | GO:0045446 | endothelial cell differentiation | 1 | 0.0027483 | S1PR1 |
| GO | GO:0034378 | chylomicron assembly | 1 | 0.0027483 | APOE |
| GO | GO:0042982 | amyloid precursor protein metabolic process | 1 | 0.0027483 | APOE |
| GO | GO:0010818 | T cell chemotaxis | 1 | 0.0027483 | CCL3 |
| GO | GO:0006707 | cholesterol catabolic process | 1 | 0.0027483 | APOE |
| GO | GO:0019068 | virion assembly | 1 | 0.0027483 | APOE |
| GO | GO:0031665 | negative regulation of lipopolysaccharide-mediated signaling pathway | 1 | 0.0027483 | LTF |
| GO | GO:0098978 | glutamatergic synapse | 2 | 0.0028311 | APOE/SPARCL1 |
| GO | GO:1900272 | negative regulation of long-term synaptic potentiation | 1 | 0.002977 | APOE |
| GO | GO:0033700 | phospholipid efflux | 1 | 0.002977 | APOE |
| GO | GO:0033690 | positive regulation of osteoblast proliferation | 1 | 0.002977 | LTF |
| GO | GO:0032780 | negative regulation of ATPase activity | 1 | 0.002977 | LTF |
| GO | GO:0050927 | positive regulation of positive chemotaxis | 1 | 0.002977 | S1PR1 |
| GO | GO:0043922 | negative regulation by host of viral transcription | 1 | 0.002977 | CCL3 |
| GO | GO:0071498 | cellular response to fluid shear stress | 1 | 0.002977 | KLF2 |
| GO | GO:0062023 | collagen-containing extracellular matrix | 2 | 0.0030858 | APOE/SPARCL1 |
| GO | GO:0035641 | locomotory exploration behavior | 1 | 0.0032057 | APOE |
| GO | GO:0000302 | response to reactive oxygen species | 1 | 0.0032057 | APOE |
| GO | GO:0042581 | specific granule | 1 | 0.0032057 | LTF |
| GO | GO:0030195 | negative regulation of blood coagulation | 1 | 0.0032057 | APOE |
| GO | GO:0034380 | high-density lipoprotein particle assembly | 1 | 0.0032057 | APOE |
| GO | GO:0042627 | chylomicron | 1 | 0.0032057 | APOE |
| GO | GO:0006954 | inflammatory response | 2 | 0.0032669 | CCL3/ACKR1 |
| GO | GO:0030516 | regulation of axon extension | 1 | 0.0034343 | APOE |
| GO | GO:0090181 | regulation of cholesterol metabolic process | 1 | 0.0034343 | APOE |
| GO | GO:0034362 | low-density lipoprotein particle | 1 | 0.0034343 | APOE |
| GO | GO:0001886 | endothelial cell morphogenesis | 1 | 0.0034343 | PECAM1 |
| GO | GO:0048246 | macrophage chemotaxis | 1 | 0.0034343 | CCL3 |
| GO | GO:1902430 | negative regulation of amyloid-beta formation | 1 | 0.0034343 | APOE |
| GO | GO:0040029 | regulation of gene expression, epigenetic | 1 | 0.0034343 | KLF2 |
| GO | GO:0009897 | external side of plasma membrane | 2 | 0.003453 | PECAM1/S1PR1 |
| GO | GO:1901653 | cellular response to peptide | 1 | 0.0036629 | KLF2 |
| GO | GO:0043249 | erythrocyte maturation | 1 | 0.0036629 | KLF2 |
| GO | GO:0071682 | endocytic vesicle lumen | 1 | 0.0036629 | APOE |
| GO | GO:0051044 | positive regulation of membrane protein ectodomain proteolysis | 1 | 0.0036629 | APOE |
| GO | GO:0043395 | heparan sulfate proteoglycan binding | 1 | 0.0038914 | APOE |
| GO | GO:0015909 | long-chain fatty acid transport | 1 | 0.0038914 | APOE |
| GO | GO:0034374 | low-density lipoprotein particle remodeling | 1 | 0.0038914 | APOE |
| GO | GO:0004698 | calcium-dependent protein kinase C activity | 1 | 0.0038914 | CCL3 |
| GO | GO:0046889 | positive regulation of lipid biosynthetic process | 1 | 0.0038914 | APOE |
| GO | GO:0055089 | fatty acid homeostasis | 1 | 0.0038914 | APOE |
| GO | GO:0030502 | negative regulation of bone mineralization | 1 | 0.0038914 | CCL3 |
| GO | GO:0090280 | positive regulation of calcium ion import | 1 | 0.0041199 | CCL3 |
| GO | GO:0048168 | regulation of neuronal synaptic plasticity | 1 | 0.0041199 | APOE |
| GO | GO:0031092 | platelet alpha granule membrane | 1 | 0.0041199 | PECAM1 |
| GO | GO:0048245 | eosinophil chemotaxis | 1 | 0.0041199 | CCL3 |
| GO | GO:0043691 | reverse cholesterol transport | 1 | 0.0041199 | APOE |
| GO | GO:0043083 | synaptic cleft | 1 | 0.0041199 | APOE |
| GO | GO:0050850 | positive regulation of calcium-mediated signaling | 1 | 0.0041199 | CCL3 |
| GO | GO:0019835 | cytolysis | 1 | 0.0043484 | GZMA |
| GO | GO:0061028 | establishment of endothelial barrier | 1 | 0.0043484 | PECAM1 |
| GO | GO:0016209 | antioxidant activity | 1 | 0.0043484 | APOE |
| GO | GO:0061136 | regulation of proteasomal protein catabolic process | 1 | 0.0043484 | APOE |
| GO | GO:0034375 | high-density lipoprotein particle remodeling | 1 | 0.0043484 | APOE |
| GO | GO:0007263 | nitric oxide mediated signal transduction | 1 | 0.0045768 | APOE |
| GO | GO:0043254 | regulation of protein-containing complex assembly | 1 | 0.0045768 | APOE |
| GO | GO:0046907 | intracellular transport | 1 | 0.0045768 | APOE |
| GO | GO:0019226 | transmission of nerve impulse | 1 | 0.0045768 | S1PR1 |
| GO | GO:0019222 | regulation of metabolic process | 1 | 0.0048051 | S1PR1 |
| GO | GO:0010875 | positive regulation of cholesterol efflux | 1 | 0.0048051 | APOE |
| GO | GO:0120020 | cholesterol transfer activity | 1 | 0.0048051 | APOE |
| GO | GO:0034361 | very-low-density lipoprotein particle | 1 | 0.0048051 | APOE |
| GO | GO:0002227 | innate immune response in mucosa | 1 | 0.0048051 | LTF |
| GO | GO:0050709 | negative regulation of protein secretion | 1 | 0.0048051 | APOE |
| GO | GO:0030500 | regulation of bone mineralization | 1 | 0.0048051 | S1PR1 |
| GO | GO:0042311 | vasodilation | 1 | 0.0048051 | APOE |
| GO | GO:0045088 | regulation of innate immune response | 1 | 0.0050334 | APOE |
| GO | GO:0019934 | cGMP-mediated signaling | 1 | 0.0050334 | APOE |
| GO | GO:0050750 | low-density lipoprotein particle receptor binding | 1 | 0.0050334 | APOE |
| GO | GO:0051000 | positive regulation of nitric-oxide synthase activity | 1 | 0.0050334 | APOE |
| GO | GO:0045807 | positive regulation of endocytosis | 1 | 0.0050334 | APOE |
| GO | GO:0043537 | negative regulation of blood vessel endothelial cell migration | 1 | 0.0050334 | APOE |
| GO | GO:0043312 | neutrophil degranulation | 2 | 0.0051576 | PECAM1/LTF |
| GO | GO:0010596 | negative regulation of endothelial cell migration | 1 | 0.0052617 | APOE |
| GO | GO:0010467 | gene expression | 1 | 0.0052617 | APOE |
| GO | GO:0019957 | C-C chemokine binding | 1 | 0.0052617 | ACKR1 |
| GO | GO:0034364 | high-density lipoprotein particle | 1 | 0.0052617 | APOE |
| GO | GO:0033344 | cholesterol efflux | 1 | 0.0054899 | APOE |
| GO | GO:0060999 | positive regulation of dendritic spine development | 1 | 0.0054899 | APOE |
| GO | GO:0048844 | artery morphogenesis | 1 | 0.0054899 | APOE |
| GO | GO:0030595 | leukocyte chemotaxis | 1 | 0.0054899 | S1PR1 |
| GO | GO:0044291 | cell-cell contact zone | 1 | 0.0059462 | PECAM1 |
| GO | GO:0045671 | negative regulation of osteoclast differentiation | 1 | 0.0059462 | CCL3 |
| GO | GO:0099560 | synaptic membrane adhesion | 1 | 0.0059462 | SPARCL1 |
| GO | GO:0007271 | synaptic transmission, cholinergic | 1 | 0.0059462 | APOE |
| GO | GO:0051497 | negative regulation of stress fiber assembly | 1 | 0.0061742 | S1PR1 |
| GO | GO:0051928 | positive regulation of calcium ion transport | 1 | 0.0064023 | CCL3 |
| GO | GO:0060349 | bone morphogenesis | 1 | 0.0064023 | LTF |
| GO | GO:0005319 | lipid transporter activity | 1 | 0.0064023 | APOE |
| GO | GO:0048020 | CCR chemokine receptor binding | 1 | 0.0066302 | CCL3 |
| GO | GO:0050840 | extracellular matrix binding | 1 | 0.0066302 | SPARCL1 |
| GO | GO:0070328 | triglyceride homeostasis | 1 | 0.0066302 | APOE |
| GO | GO:0051603 | proteolysis involved in cellular protein catabolic process | 1 | 0.0066302 | GZMA |
| GO | GO:0045893 | positive regulation of transcription, DNA-templated | 2 | 0.006797 | APOE/KLF2 |
| GO | GO:0051930 | regulation of sensory perception of pain | 1 | 0.0068582 | CCL3 |
| GO | GO:0007159 | leukocyte cell-cell adhesion | 1 | 0.0068582 | PECAM1 |
| GO | GO:0043539 | protein serine/threonine kinase activator activity | 1 | 0.007086 | LTF |
| GO | GO:0043392 | negative regulation of DNA binding | 1 | 0.007086 | GZMA |
| GO | GO:0001937 | negative regulation of endothelial cell proliferation | 1 | 0.0073139 | APOE |
| GO | GO:0043542 | endothelial cell migration | 1 | 0.0073139 | PECAM1 |
| GO | GO:0035633 | maintenance of blood-brain barrier | 1 | 0.0073139 | PECAM1 |
| GO | GO:0048247 | lymphocyte chemotaxis | 1 | 0.0075417 | CCL3 |
| GO | GO:0030669 | clathrin-coated endocytic vesicle membrane | 1 | 0.0075417 | APOE |
| GO | GO:0042056 | chemoattractant activity | 1 | 0.0077694 | CCL3 |
| GO | GO:0030032 | lamellipodium assembly | 1 | 0.0077694 | S1PR1 |
| GO | GO:0006641 | triglyceride metabolic process | 1 | 0.0077694 | APOE |
| GO | GO:0001530 | lipopolysaccharide binding | 1 | 0.0077694 | LTF |
| GO | GO:0051482 | positive regulation of cytosolic calcium ion concentration involved in phospholipase C-activating G protein-coupled signaling pathway | 1 | 0.0077694 | S1PR1 |
| GO | GO:0001817 | regulation of cytokine production | 1 | 0.0077694 | LTF |
| GO | GO:0007616 | long-term memory | 1 | 0.0079971 | APOE |
| GO | GO:0004869 | cysteine-type endopeptidase inhibitor activity | 1 | 0.0079971 | LTF |
| GO | GO:0031663 | lipopolysaccharide-mediated signaling pathway | 1 | 0.0079971 | CCL3 |
| GO | GO:0031640 | killing of cells of other organism | 1 | 0.0082248 | LTF |
| GO | GO:0098742 | cell-cell adhesion via plasma-membrane adhesion molecules | 1 | 0.0084524 | PECAM1 |
| GO | GO:0032991 | protein-containing complex | 2 | 0.008461 | PECAM1/LTF |
| GO | GO:0031226 | intrinsic component of plasma membrane | 1 | 0.0086799 | S1PR1 |
| GO | GO:0043491 | protein kinase B signaling | 1 | 0.0089074 | CCL3 |
| GO | GO:0043407 | negative regulation of MAP kinase activity | 1 | 0.0091349 | APOE |
| GO | GO:0045429 | positive regulation of nitric oxide biosynthetic process | 1 | 0.0091349 | KLF2 |
| GO | GO:0032715 | negative regulation of interleukin-6 production | 1 | 0.0093623 | KLF2 |
| GO | GO:0001772 | immunological synapse | 1 | 0.0093623 | GZMA |
| GO | GO:0019731 | antibacterial humoral response | 1 | 0.0095897 | LTF |
| GO | GO:0045071 | negative regulation of viral genome replication | 1 | 0.0095897 | LTF |
| GO | GO:0001895 | retina homeostasis | 1 | 0.0095897 | LTF |
| GO | GO:0070062 | extracellular exosome | 3 | 0.00991 | PECAM1/APOE/LTF |
| GO | GO:0120009 | intermembrane lipid transfer | 1 | 0.0100443 | APOE |
| GO | GO:0002548 | monocyte chemotaxis | 1 | 0.0100443 | CCL3 |
| GO | GO:0050918 | positive chemotaxis | 1 | 0.0100443 | CCL3 |
| GO | GO:0071407 | cellular response to organic cyclic compound | 1 | 0.0104987 | CCL3 |
| GO | GO:0048156 | tau protein binding | 1 | 0.0104987 | APOE |
| GO | GO:0048856 | anatomical structure development | 1 | 0.0104987 | SPARCL1 |
| GO | GO:0008009 | chemokine activity | 1 | 0.01118 | CCL3 |
| GO | GO:0030155 | regulation of cell adhesion | 1 | 0.011407 | S1PR1 |
| GO | GO:0032760 | positive regulation of tumor necrosis factor production | 1 | 0.011634 | CCL3 |
| GO | GO:0071902 | positive regulation of protein serine/threonine kinase activity | 1 | 0.011634 | LTF |
| GO | GO:0004930 | G protein-coupled receptor activity | 2 | 0.0117328 | S1PR1/ACKR1 |
| GO | GO:0043525 | positive regulation of neuron apoptotic process | 1 | 0.0120877 | CCL3 |
| GO | GO:0019730 | antimicrobial humoral response | 1 | 0.0125413 | LTF |
| GO | GO:1904724 | tertiary granule lumen | 1 | 0.012768 | LTF |
| GO | GO:1903561 | extracellular vesicle | 1 | 0.012768 | APOE |
| GO | GO:0007266 | Rho protein signal transduction | 1 | 0.0129947 | PECAM1 |
| GO | GO:0032731 | positive regulation of interleukin-1 beta production | 1 | 0.0132214 | CCL3 |
| GO | GO:0031532 | actin cytoskeleton reorganization | 1 | 0.0132214 | S1PR1 |
| GO | GO:0006959 | humoral immune response | 1 | 0.0134479 | LTF |
| GO | GO:0048661 | positive regulation of smooth muscle cell proliferation | 1 | 0.0134479 | S1PR1 |
| GO | GO:0070830 | bicellular tight junction assembly | 1 | 0.0136745 | PECAM1 |
| GO | GO:0045669 | positive regulation of osteoblast differentiation | 1 | 0.013901 | LTF |
| GO | GO:0006909 | phagocytosis | 1 | 0.013901 | PECAM1 |
| GO | GO:0007193 | adenylate cyclase-inhibiting G protein-coupled receptor signaling pathway | 1 | 0.013901 | S1PR1 |
| GO | GO:0010977 | negative regulation of neuron projection development | 1 | 0.0141274 | APOE |
| GO | GO:0061844 | antimicrobial humoral immune response mediated by antimicrobial peptide | 1 | 0.0143538 | LTF |
| GO | GO:0035580 | specific granule lumen | 1 | 0.0143538 | LTF |
| GO | GO:0001523 | retinoid metabolic process | 1 | 0.0145802 | APOE |
| GO | GO:0005886 | plasma membrane | 4 | 0.0148372 | PECAM1/S1PR1/APOE/ACKR1 |
| GO | GO:0005737 | cytoplasm | 4 | 0.0148933 | CCL3/S1PR1/APOE/LTF |
| GO | GO:0005518 | collagen binding | 1 | 0.0154851 | SPARCL1 |
| GO | GO:0070301 | cellular response to hydrogen peroxide | 1 | 0.0154851 | KLF2 |
| GO | GO:0001664 | G protein-coupled receptor binding | 1 | 0.0154851 | S1PR1 |
| GO | GO:0042531 | positive regulation of tyrosine phosphorylation of STAT protein | 1 | 0.0157113 | PECAM1 |
| GO | GO:0006952 | defense response | 1 | 0.017067 | ACKR1 |
| GO | GO:0008203 | cholesterol metabolic process | 1 | 0.0172928 | APOE |
| GO | GO:0006816 | calcium ion transport | 1 | 0.0172928 | CCL3 |
| GO | GO:0098869 | cellular oxidant detoxification | 1 | 0.0175186 | APOE |
| GO | GO:0030593 | neutrophil chemotaxis | 1 | 0.0177443 | CCL3 |
| GO | GO:0001503 | ossification | 1 | 0.0177443 | LTF |
| GO | GO:0001540 | amyloid-beta binding | 1 | 0.0184211 | APOE |
| GO | GO:0050829 | defense response to Gram-negative bacterium | 1 | 0.0186467 | LTF |
| GO | GO:0000902 | cell morphogenesis | 1 | 0.0186467 | KLF2 |
| GO | GO:0050729 | positive regulation of inflammatory response | 1 | 0.0186467 | CCL3 |
| GO | GO:0019722 | calcium-mediated signaling | 1 | 0.0188721 | CCL3 |
| GO | GO:0009636 | response to toxic substance | 1 | 0.0188721 | CCL3 |
| GO | GO:0042632 | cholesterol homeostasis | 1 | 0.0193229 | APOE |
| GO | GO:0035264 | multicellular organism growth | 1 | 0.0193229 | KLF2 |
| GO | GO:0050731 | positive regulation of peptidyl-tyrosine phosphorylation | 1 | 0.0197736 | PECAM1 |
| GO | GO:0042060 | wound healing | 1 | 0.0204492 | PECAM1 |
| GO | GO:0016301 | kinase activity | 1 | 0.0204492 | CCL3 |
| GO | GO:0030141 | secretory granule | 1 | 0.0211244 | LTF |
| GO | GO:0007165 | signal transduction | 2 | 0.0213137 | PECAM1/SPARCL1 |
| GO | GO:0006887 | exocytosis | 1 | 0.0213493 | CCL3 |
| GO | GO:0071346 | cellular response to interferon-gamma | 1 | 0.0217992 | CCL3 |
| GO | GO:0050728 | negative regulation of inflammatory response | 1 | 0.0217992 | APOE |
| GO | GO:0005634 | nucleus | 4 | 0.0224585 | KLF2/APOE/LTF/GZMA |
| GO | GO:0030667 | secretory granule membrane | 1 | 0.0226982 | PECAM1 |
| GO | GO:0001649 | osteoblast differentiation | 1 | 0.0242698 | CCL3 |
| GO | GO:0005543 | phospholipid binding | 1 | 0.0249426 | APOE |
| GO | GO:0010976 | positive regulation of neuron projection development | 1 | 0.025391 | APOE |
| GO | GO:0007189 | adenylate cyclase-activating G protein-coupled receptor signaling pathway | 1 | 0.0258391 | S1PR1 |
| GO | GO:0009617 | response to bacterium | 1 | 0.0262871 | GZMA |
| GO | GO:0031175 | neuron projection development | 1 | 0.026511 | APOE |
| GO | GO:0045944 | positive regulation of transcription by RNA polymerase II | 2 | 0.0274079 | S1PR1/KLF2 |
| GO | GO:0002576 | platelet degranulation | 1 | 0.0283007 | PECAM1 |
| GO | GO:0055037 | recycling endosome | 1 | 0.0289711 | ACKR1 |
| GO | GO:0005506 | iron ion binding | 1 | 0.0289711 | LTF |
| GO | GO:0003677 | DNA binding | 2 | 0.029643 | LTF/KLF2 |
| GO | GO:0043524 | negative regulation of neuron apoptotic process | 1 | 0.0314256 | APOE |
| GO | GO:0030182 | neuron differentiation | 1 | 0.0316485 | S1PR1 |
| GO | GO:0046983 | protein dimerization activity | 1 | 0.0318713 | APOE |
| GO | GO:0072562 | blood microparticle | 1 | 0.0320941 | APOE |
| GO | GO:0098609 | cell-cell adhesion | 1 | 0.0323168 | PECAM1 |
| GO | GO:0008360 | regulation of cell shape | 1 | 0.034097 | CCL3 |
| GO | GO:0008289 | lipid binding | 1 | 0.0343193 | APOE |
| GO | GO:0051092 | positive regulation of NF-kappaB transcription factor activity | 1 | 0.0360962 | LTF |
| GO | GO:0007156 | homophilic cell adhesion via plasma membrane adhesion molecules | 1 | 0.0367618 | PECAM1 |
| GO | GO:0006898 | receptor-mediated endocytosis | 1 | 0.0369836 | APOE |
| GO | GO:0051897 | positive regulation of protein kinase B signaling | 1 | 0.0376487 | CCL3 |
| GO | GO:0005198 | structural molecule activity | 1 | 0.0380918 | APOE |
| GO | GO:0090090 | negative regulation of canonical Wnt signaling pathway | 1 | 0.0398625 | APOE |
| GO | GO:0001934 | positive regulation of protein phosphorylation | 1 | 0.0403048 | PECAM1 |
| GO | GO:0005911 | cell-cell junction | 1 | 0.0414096 | PECAM1 |
| GO | GO:0042802 | identical protein binding | 2 | 0.0417248 | CCL3/APOE |
| GO | GO:0043123 | positive regulation of I-kappaB kinase/NF-kappaB signaling | 1 | 0.0418512 | LTF |
| GO | GO:0050900 | leukocyte migration | 1 | 0.0433953 | PECAM1 |
| GO | GO:0001701 | in utero embryonic development | 1 | 0.0455975 | KLF2 |
| GO | GO:0038023 | signaling receptor activity | 1 | 0.0482341 | ACKR1 |
| GO | GO:0004672 | protein kinase activity | 1 | 0.0486729 | CCL3 |

**Abbreviation**: GO, gene ontology; KEGG, Kyoto Encyclopedia of Genes and Genomes.
